# The long-term effects of genomic selection: 1. Response to selection, additive genetic variance, and genetic architecture

**DOI:** 10.1186/s12711-022-00709-7

**Published:** 2022-03-07

**Authors:** Yvonne C. J. Wientjes, Piter Bijma, Mario P. L. Calus, Bas J. Zwaan, Zulma G. Vitezica, Joost van den Heuvel

**Affiliations:** 1 Animal Breeding and Genomics, Wageningen University & Research, 6700 AH Wageningen, The Netherlands; 2 Laboratory of Genetics, Wageningen University & Research, 6700 AH Wageningen, The Netherlands; 3 INRAE, INP, UMR 1388 GenPhySE, 31326 Castanet-Tolosan, France

## Abstract

**Background:**

Genomic selection has revolutionized genetic improvement in animals and plants, but little is known about its long-term effects. Here, we investigated the long-term effects of genomic selection on response to selection, genetic variance, and the genetic architecture of traits using stochastic simulations. We defined the genetic architecture as the set of causal loci underlying each trait, their allele frequencies, and their statistical additive effects. We simulated a livestock population under 50 generations of phenotypic, pedigree, or genomic selection for a single trait, controlled by either only additive, additive and dominance, or additive, dominance, and epistatic effects. The simulated epistasis was based on yeast data.

**Results:**

Short-term response was always greatest with genomic selection, while response after 50 generations was greater with phenotypic selection than with genomic selection when epistasis was present, and was always greater than with pedigree selection. This was mainly because loss of genetic variance and of segregating loci was much greater with genomic and pedigree selection than with phenotypic selection. Compared to pedigree selection, selection response was always greater with genomic selection. Pedigree and genomic selection lost a similar amount of genetic variance after 50 generations of selection, but genomic selection maintained more segregating loci, which on average had lower minor allele frequencies than with pedigree selection. Based on this result, genomic selection is expected to better maintain genetic gain after 50 generations than pedigree selection. The amount of change in the genetic architecture of traits was considerable across generations and was similar for genomic and pedigree selection, but slightly less for phenotypic selection. Presence of epistasis resulted in smaller changes in allele frequencies and less fixation of causal loci, but resulted in substantial changes in statistical additive effects across generations.

**Conclusions:**

Our results show that genomic selection outperforms pedigree selection in terms of long-term genetic gain, but results in a similar reduction of genetic variance. The genetic architecture of traits changed considerably across generations, especially under selection and when non-additive effects were present. In conclusion, non-additive effects had a substantial impact on the accuracy of selection and long-term response to selection, especially when selection was accurate.

**Supplementary Information:**

The online version contains supplementary material available at 10.1186/s12711-022-00709-7﻿.

## Background

Animal breeding has substantially increased the performance of livestock populations over the last century [1, 2]. This has been achieved by selecting the genetically best performing individuals to produce the next generation based on own performance and/or performances of relatives. In spite of the strong selection, these pedigree-based selection methods have proven to be sustainable, as genetic variation and rates of genetic gain have been stable for many generations in several animal and plant species, both in commercial breeding programs and in experimental selection lines [3–6].

Recently, genomic selection has revolutionized animal breeding [7, 8]. Within genomic selection, genotypes on several thousands of DNA markers covering the genome, along with recorded phenotypes, are used to identify the genetically best animals. In some breeding programs, genomic selection has doubled the annual rate of genetic gain compared to pedigree-based selection [9, 10]. Arguably, genomic selection enables selection for lowly-heritable traits [11, 12] and for traits that are difficult or expensive to measure [13–15], for which pedigree-based selection is generally not very effective. These properties have resulted in the rapid implementation of genomic selection in animal breeding programs worldwide [8, 16–18].

The accuracy of selection and, thereby, the genetic gain from genomic selection are affected by the genetic architecture of traits [19–21], i.e. the set of causal loci that underlie each trait, their allele frequencies, and their statistical additive effects. The genetic architecture is largely unknown for most traits, including those under selection in breeding programmes, but is known to evolve over time as a result of new mutations and changes in allele frequencies due to selection and drift [1, 2, 22–26]. When interactions are present within (dominance) or between (epistasis) loci, the statistical additive effects (also known as allele substitution effects) depend on the allele frequencies at the locus itself, as well as on those of interacting loci. This means that functional dominance and epistatic effects contribute to additive genetic variation, depending on allele frequencies [27–29]. Although interactions between loci are known and common [30–33], not much is known about their interaction network or how those interactions contribute to genetic variance components or how those contributions change over generations as a result of drift or selection. To date, the network of genetic interactions has been most intensively studied in yeast, where 90% of the loci associated with a trait were found to be involved in at least one interaction, with only few interactions for most loci, and many interactions for only a few loci [34–36]. Boone et al*.* [36] and Mackay [37] argue that it is likely that this network of genetic interactions is similar in other species, including livestock and humans.

We hypothesize that genomic selection accelerates changes in the genetic architecture of traits across generations, which can affect long-term genetic gain. The reason for this is not only that genomic selection is more effective, but also because the distribution of the selection pressure across the genome is different with genomic selection. Classical selection methods based on pedigree relationships distribute selection pressure relatively evenly across the genome [38]. This is in contrast to genomic selection, which puts less weight on loci with rare alleles [38, 39]. Thus, genomic selection methods select more strongly genomic regions that surround loci with a large contribution to the additive genetic variance and may significantly increase changes in allele frequencies at those loci [40]. Therefore, although genomic selection may substantially accelerate the rate of genetic gain in the short-term, we expect that by ignoring regions with a smaller contribution to additive genetic variance, genomic selection increases the risk of losing rare favourable alleles or may fail to increase the frequency of such alleles [41–43]. Loss of rare favourable alleles reduces genetic variation and genetic gain in the long term [38] and also limits the potential for future selection on other traits. However, currently, these expectations have not been investigated in detail or tested in breeding populations.

Therefore, the aim of this study was to investigate the long-term effects of genomic selection on the genetic architecture of traits. Using simulation, we compared genomic selection to phenotypic and pedigree-based selection. We investigated the impact of those selection methods on the rate of genetic gain, the loss in genetic variance, and the change in genetic architecture over 50 generations of selection. The results provide more insight on the long-term evolution of the genetic architecture and genetic variation of traits under different selection methods.

## Methods

### Simulated population

We simulated a livestock population over 50 generations of selection. As a first step, we constructed a historical population in which selection was absent and mating was at random, using the QMSim software [44]. The first 2000 generations (generation − 3050 to − 1050) consisted of 1500 individuals, after which the size of the population gradually decreased to 100 over 500 generations (generation − 1050 to − 550) to generate linkage disequilibrium. This was followed by a gradual increase in population size to 1500 over 500 generations (generation − 550 to − 50). From the last historical generation (generation − 50), 100 females and 100 males were randomly sampled and used as input for our own custom Fortran program, in which they were randomly mated (mating ratio 1:1) with a litter size of 10 (5 females and 5 males). In each of the next 50 discrete generations, 100 females and 100 males were randomly sampled and mated to build-up mutation-drift equilibrium (generation − 50 to 0), using a randomly selected proportion of 0.2. Generation 0 formed the base population for the 50 generations of selection. In the following generations, we used truncation selection to select the best 100 females and 100 males, which were randomly mated using a mating ratio of 1:1 and a litter size of 10 (5 females and 5 males), resulting in a selected proportion of 0.2 for both females and males. Five selection methods were used, as explained below. This process was replicated 20 times.

### Genome

The simulated genome contained 10 chromosomes of 100 cM each. The number of recombination events per chromosome was sampled from a Poisson distribution with on average one recombination per chromosome, uniformly distributed across the chromosome.

In the historical population, 200,000 randomly spaced bi-allelic loci per chromosome were simulated with a recurrent mutation rate of $5 \times 10^{ - 5}$ to maintain at most two alleles at a locus. The population structure and mutation rate resulted in a U-shaped allele frequency distribution of the loci in the historical population. In the last historical generation, 2000 segregating loci were randomly selected to become causal loci. By randomly selecting 200 loci from each of 100 equally-sized bins based on allele frequency, another set of 20,000 segregating loci were selected as genetic markers. This resulted in a uniform distribution of the allele frequency of the markers, reflecting the ascertainment bias of markers that are typically placed on commercial marker chips [45–47].

After the historical population, the number of mutations per individual was sampled from a Poisson distribution with an average of 0.6, which resulted in a mutational variance of ~ 0.001$\sigma_{e}^{2}$ under our simulated additive model (as explained later), as is often observed in real populations [48–50]. A random 4000 loci that did not segregate in the last generation of the historical population were chosen to be subject to mutation. The loci and effects of the mutations were recycled to limit the computational requirement. In each generation, a locus was drawn from the potential loci that did not segregate at that time, while maximizing the time between two mutations at the same locus. As such, each of the 4000 loci was used on average once each 6 to 7 generations. We believe that recycling the same mutations does not impact the results of our study, because the vast majority of the mutations are lost in the first generation due to drift, regardless of their effect.

### Genetic and phenotypic values

Three genetic models were used to simulate phenotypic values; a model with only additive effects (A), a model with additive and dominance effects (AD), and a model with additive, dominance, and epistatic effects (ADE). In the last historical generation, functional (or biological) additive and dominance effects were assigned to all 2000 causal loci and to the 4000 loci for mutations. At the same time, epistatic effects were assigned to 90% of those loci, as was observed for the yeast data [34].

Functional additive effects ($a$) were sampled from a normal distribution with mean 0 and standard deviation 1. Functional dominance effects ($d$) were simulated proportional to the additive effect by first sampling a dominance degree ($dd$) for each locus from a normal distribution with mean 0.2 and standard deviation 0.3 [51–53], and then computing the dominance effect of locus $i$ as $d_{i} = dd_{i} \left| {a_{i} } \right|$. This resulted in mostly positive dominance effects, with a bit of overdominance, as was empirically observed in pigs [51].

**Fig. 1:**
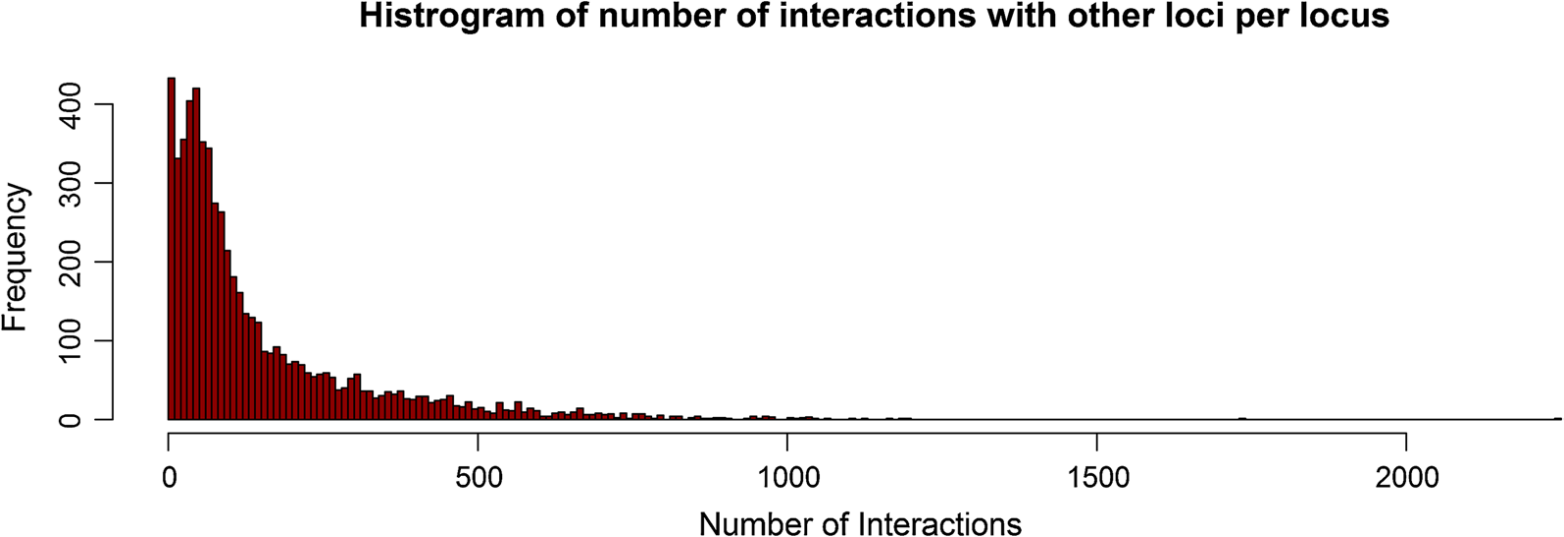
Histogram of the number of interactions per causal locus

Only pairwise epistatic effects were simulated, because higher-order interactions have little effect on the phenotypic variance when the allele frequency distribution is U-shaped [27, 28, 54]. The number of interactions per locus was sampled using the interaction network found between the ~ 6000 genes in yeast [34, 55], with many loci with few interactions and few loci with many interactions (Fig. 1). This was done by creating an interaction matrix from the network in yeast, with elements of 1 when loci interacted and 0 otherwise. From this matrix, columns and corresponding rows were selected for all loci with an interaction. For the interaction between loci $B$ and $C$, nine epistatic degrees ($\varepsilon$) were independently sampled from a normal distribution with mean 0 and standard deviation 0.45, one for each of the nine possible two-locus genotype combinations. The sampled $\varepsilon$ were used to create nine epistatic effects ($e$) for each interaction as $e = \varepsilon \sqrt {\left| {a_{B} a_{C} } \right|}$ (Table 1), resulting in larger epistatic effects for loci with a larger additive effect. This resulted in the creation of all types of epistasis, i.e. additive-by-additive, additive-by-dominance, and dominance-by-dominance. However, by simulating the epistatic effects in this random manner, the simulated epistatic effects also contributed to functional additive or dominance effects (Table 1). When computing functional additive, dominance, and epistatic variance components, we first redistributed the simulated epistatic effects in the correct underlying functional effects. This was achieved by solving for each interaction the nine equations in Table 1 for the eight separate functional additive ($a_{B}$ and $a_{C}$), dominance ($d_{B}$ and $d_{C}$), additive-by-additive ($k$), additive-by-dominance ($l$ and $m$) and dominance-by-dominance ($n$) epistatic effects that were underlying that interaction and adding the additive and dominance effects to the functional additive and dominance effects of the corresponding loci.

**Table 1:** Simulated epistatic model for two-locus interactions

Genotype locus *B*	Genotype locus *C*		
	*CC*	*Cc*	*cc*
*BB*	$e_{00} = \mu + a_{B} + a_{C} + k$	$e_{01} = \mu + a_{B} + d_{C} + m$	$e_{02} = \mu + a_{B} - a_{C} - k$
*Bb*	$e_{10} = \mu + d_{B} + a_{C} + l$	$e_{11} = \mu + d_{B} + d_{C} + n$	$e_{12} = \mu + d_{B} - a_{C} - l$
*bb*	$e_{20} = \mu - a_{B} + a_{C} - k$	$e_{21} = \mu - a_{B} + d_{C} - m$	$e_{22} = \mu - a_{B} - a_{C} + k$

First, nine epistatic effects ($e_{00}$ to $e_{22}$) were simulated randomly, by sampling for each effect an epistatic degree ($\varepsilon$) from a normal distribution and scaling them by the additive effects of the two loci (i.e. $e_{00} = \varepsilon_{00} \sqrt {\left| {a_{B} a_{C} } \right|}$). Then, those nine epistatic effects were used to estimate the separate functional additive ($a_{B}$ and $a_{C}$), dominance ($d_{B}$ and $d_{C}$), additive-by-additive ($k$), additive-by-dominance ($l$ and $m$) and dominance-by-dominance ($n$) epistatic effects that were underlying those epistatic effects

The functional genetic effects were combined with the genotypes of the individuals to calculate total genetic values. For each individual, a residual term was also sampled from a normal distribution with mean zero and standard deviation equal to the square root of 1.5 times the variance of total genetic values in the last historical generation, resulting in a broad sense heritability of 0.4 in that generation.

### Statistical effects

The natural and orthogonal interaction approach (NOIA) [56, 57] was applied in each generation to compute statistical additive and dominance effects based on the functional additive, dominance, and epistatic effects of all causal loci (the 2000 segregating causal loci and the 4000 loci for mutations) and their allele frequencies [52]. For each locus $i,$ the part of the dominance effect that is statistically additive was calculated as $\left( {1 - 2p_{i} } \right)d_{i},$ where $p_{i}$ is the frequency of the focal allele (i.e. allele $B$ for locus $B$ in Table 1). For each interaction between loci $B$ (with alleles $b$ and $B$) and $C$ (with alleles $c$ and $C$), part of the functional epistasis is converted into statistical additive and statistical dominance effects that were computed from three components: (1) a vector ${\mathbf{y}}$ with functional epistatic effects, $${\mathbf{y^{\prime}}} = \left[ {\begin{array}{*{20}c} {e_{00} } & {e_{10} } & {e_{20} } & {e_{01} } & {e_{11} } & {e_{21} } & {e_{02} } & {e_{12} } & {e_{22} } \\ \end{array} } \right],$$ (2) a 9 × 9 diagonal matrix ${\mathbf{D}}$ with the expected frequencies of the two-locus haplotypes, assuming that loci segregate independently, and (3) a 9 × 9 matrix ${\mathbf{W}}$ with the mean and orthogonal contrasts for the two loci, constructed as ${\mathbf{W}} = {\mathbf{W}}_{B} \otimes {\mathbf{W}}_{C},$ with:

$${\mathbf{W}}_{B} = \left[ {\begin{array}{*{20}ll} 1 & {p_{Bb} + 2p_{bb} } & {\frac{{ - 2p_{Bb} p_{bb} }}{{p_{BB} + p_{bb} - \left( {p_{BB} - p_{bb} } \right)^{2} }}} \\ 1 & {p_{Bb} + 2p_{bb} - 1} & {\frac{{4p_{BB} p_{bb} }}{{p_{BB} + p_{bb} - \left( {p_{BB} - p_{bb} } \right)^{2} }}} \\ 1 & {p_{Bb} + 2p_{bb} - 2} & {\frac{{ - 2p_{BB} p_{Bb} }}{{p_{BB} + p_{bb} - \left( {p_{BB} - p_{bb} } \right)^{2} }}} \\ \end{array} } \right],$$

where $p_{BB}$, $p_{Bb}$, and $p_{bb}$ represent the frequencies of the genotypes $BB$, $Bb$, and $bb$ for locus $B$. The statistical effects related to the interaction between loci $B$ and $C$ then follow from:

$$\begin{aligned} {\mathbf{b}}_{BC} & = \left[ {\begin{array}{*{20}ll} \mu & {\alpha_{BC}^{B} } & {\delta_{BC}^{B} } & {\alpha_{BC}^{C} } & {\left( {\alpha \alpha } \right)_{BC} } & {\left( {\delta \alpha } \right)_{BC} } & {\delta_{BC}^{C} } & {\left( {\alpha \delta } \right)_{BC} } & {\left( {\delta \delta } \right)_{BC} } \\ \end{array} } \right]^{\prime } \\ & = \left( {{\mathbf{W^{\prime}DW}}} \right)^{ - 1} {\mathbf{W^{\prime}Dy}}, \\ \end{aligned}$$

where $\mu$ is a general mean, $\alpha^{x}$ is the statistical additive effect related to locus $x$, $\delta^{x}$ is the statistical dominance effect related to locus $x$, and $\alpha \alpha$*,* $\alpha \delta$*,* $\delta \alpha$*,* and $\delta \delta$ are, respectively, the additive-by-additive, additive-by-dominance, dominance-by-additive, and dominance-by-dominance epistatic effects. Note that the NOIA model was run separately for each pair of interacting loci, such that only the functional interaction effects were considered and not the functional additive and dominance effects. Therefore $\alpha_{BC}^{B} = \left( {p_{C} - q_{C} } \right)k + 2p_{C} q_{C} m + \left( {1 - 2p_{B} } \right)\left( {p_{C} - q_{C} } \right)l + 2p_{C} q_{C} \left( {1 - 2p_{B} } \right)n,$ and $\delta_{BC}^{B} = - \left( {1 - 2p_{C} } \right)l + 2p_{C} \left( {1 - p_{C} } \right)n;$ where $k$, $l$, $m$, and $n$ are the additive-by-additive, dominancy-by-additive, additive-by-dominance and dominance-by-dominance functional epistatic effects (Table 1), respectively.

The total statistical additive effect at locus $i$ was calculated as:

$$\alpha_{i} = a_{i} + \left( {1 - 2p_{i} } \right)d_{i} + \sum \alpha_{ij}^{i} ,$$

and the total statistical dominance effect as:

$$\delta_{i} = d_{i} + \sum \delta_{ij}^{i} ,$$

where the summations were taken across all interactions that involved locus $i$.

The statistical additive effect was used to compute the total additive genetic value (i.e. true breeding value) across all loci $i$ of each individual as $A = \sum w_{{a_{i} }} \alpha_{i}$, with:

$$w_{{a_{i} }} = \left\{ {\begin{array}{*{20}ll} {p_{Bb} + 2p_{bb} } \\ {p_{Bb} + 2p_{bb} - 1} \\ {p_{Bb} + 2p_{bb} - 2} \\ \end{array} } \right.\;{\text{for}}\;{\text{genotypes}}\;\left\{ {\begin{array}{*{20}ll} {BB} \\ {Bb} \\ {bb} \\ \end{array} } \right..$$

In the same way, the statistical dominance effect was used to compute the total dominance deviation across all loci $i$ of each individual as $D = \sum w_{{d_{i} }} \delta_{i}$, with:

$$w_{{d_{i} }} = \left\{ {\begin{array}{*{20}ll} {\frac{{ - 2p_{Bb} p_{bb} }}{{p_{BB} + p_{bb} - \left( {p_{BB} - p_{bb} } \right)^{2} }}} \\ {\frac{{4p_{BB} p_{bb} }}{{p_{BB} + p_{bb} - \left( {p_{BB} - p_{bb} } \right)^{2} }}} \\ {\frac{{ - 2p_{BB} p_{Bb} }}{{p_{BB} + p_{bb} - \left( {p_{BB} - p_{bb} } \right)^{2} }}} \\ \end{array} } \right.\;{\text{for}}\;{\text{genotypes}}\;\left\{ {\begin{array}{*{20}ll} {BB} \\ {Bb} \\ {bb} \\ \end{array} } \right..$$

By definition, the variance in $A$ across all individuals is the additive genetic variance, the variance in $D$ across all individuals is the dominance genetic variance, and the variance in total genetic values across all individuals is the total genetic variance. The total genetic variance minus the additive and dominance variance is the epistatic variance.

### Selection methods

Five methods were used to select the sires and dams of the next generation. As a base line for comparison, the first method randomly selected the parents (RANDOM) and was meant to capture the impact of drift alone. The second method selected the individuals with the highest phenotypic values to become the parents of the next generation (MASS). The third method selected individuals with the highest estimated breeding values using a pedigree best linear unbiased prediction (BLUP) model that included own performance information of the selection candidates (PBLUP_OP). The fourth and fifth methods selected individuals with the highest genomic estimated breeding values from a genomic BLUP model that either included own performance information of the selection candidates (GBLUP_OP) or not (GBLUP_NoOP).

Breeding value estimation for the last three methods was performed using the MTG2 software [58]. Each generation, breeding values were estimated simultaneously with estimating the variance components, using the phenotypic information of the previous three generations, and for PBLUP_OP and GBLUP_OP using also phenotypic information of the present generation. The PBLUP method used a relationship matrix based on a pedigree that included all individuals from the present generation and the previous eight generations. The GBLUP methods used a relationship matrix based on marker genotypes of the present generation and the previous three generations, computed using Method 1 of VanRaden [59], with allele frequencies estimated based on the genotype data of those generations. The model for breeding value estimation included a fixed mean, a random additive genetic effect, a random litter effect, and a residual. The random litter effect was included to capture resemblance between full sibs due to non-additive genetic effects, which could otherwise bias the estimated breeding values. Although dominance and epistatic effects were simulated, these were not included in the breeding value estimation model, because additive models are generally used in breeding programs and only the breeding value is transmitted to the offspring.

### Comparing genetic models and selection methods

The three genetic models (A, AD, and ADE) and five selection methods resulted in 15 scenarios that were applied to each of the 20 replicates of the simulated population. The scenarios were compared based on accuracies of selection, phenotypic trend, additive genetic variance, additive genic variance (calculated as the sum of $2p_{i} \left( {1 - p_{i} } \right)\alpha_{i}^{2}$ across all causal loci $i$), expected heterozygosity, average minor allele frequency (MAF), and number of segregating causal loci across the 50 generations of selection. Accuracy of selection within a generation was calculated as the correlation between the true and estimated breeding values among animals in that generation.

One of our main aims was to evaluate how fast the genetic architecture of the trait changed due to selection. The genetic architecture can change because: (1) the subset of loci affecting the trait changes due to new mutations and loci becoming fixed, (2) the allele frequencies of those loci change, which can result in changes in the proportion of the additive genetic variance explained by each locus, or (3) the statistical additive effects of the loci change as a result of changes in allele frequencies and of non-additive effects, which can also change the proportion of additive genetic variance explained by a locus. To quantify changes in genetic architecture, we defined three criteria that each reflected one of those mechanisms, namely: (1) the Jaccard index [60] for the segregating causal loci, (2) the correlation of allele frequencies at those loci between generations, and (3) the correlation of statistical additive effects at those loci between generations. For the first criterion, we calculated the Jaccard index [60] between generation 0 (before selection) and each of the generations after selection as the number of overlapping segregating loci divided by the total number of segregating loci in the two generations. For the second criterion, we calculated the correlation of allele frequencies in generation 0 with those in each subsequent generation, using only the loci that segregated in generation 0 and still segregated in the generation in question. For the third criterion, we calculated the correlation of statistical additive effects in generation 0 with those in a subsequent generation, again including only loci that segregated in both generations.

## Results

### Properties of the simulated population

The distribution of allele frequencies of the segregating causal loci was strongly U-shaped (see Additional file 2: Fig. S1) and comparable to the distribution of allele frequencies of segregating loci that is observed in sequence data of livestock populations [61–64]. In the RANDOM scenario, where no selection was performed, the pattern of allele frequencies remained similar across generations, indicating that the population was approximately in mutation-drift equilibrium. Moreover, the pattern of linkage disequilibrium in the population (see Additional file 2: Fig. S2) was similar to that found in pig and chicken populations [65–67]. This indicates that the effective population size of the simulated population was comparable to that in real livestock populations, which ranges from 40 to 130 [40, 68–70].

With model ADE, epistasis at the functional level was abundant and 49% of the variation in the total genetic value was generated by functional epistatic effects and only 19% by functional additive effects. However, most of the genetic variance at the statistical level was additive (62%) or due to dominance (33%), and only 5% was epistatic variance in generation 0, which is reasonably close to results for litter size in pigs [71]. The broad-sense heritability was set to 0.4 for all genetic models, resulting in a narrow-sense heritability of ~ 0.25 for model ADE. This heritability was considerably lower than the narrow-sense heritability of ~ 0.40 for model A and ~ 0.38 for model AD. Altogether, those parameters indicate that the genetic architecture that was simulated based on model ADE could represent the genetic architecture of a quantitative trait in a livestock population.

### Accuracy of selection

**Fig. 2:**
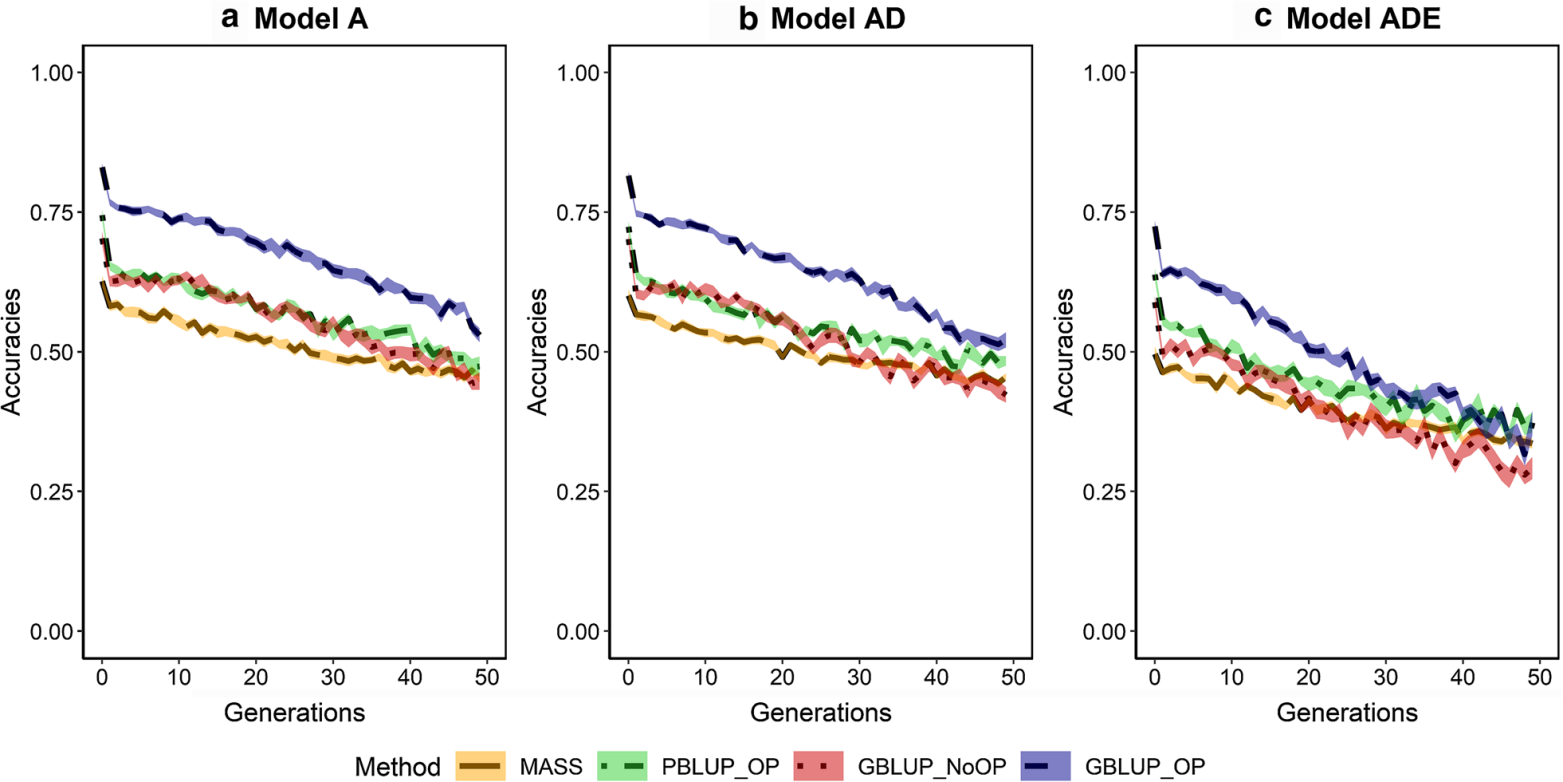
Accuracy of selection across generations for four selection methods and three genetic models. The four selection methods were: MASS selection, PBLUP selection with own performance (PBLUP_OP), GBLUP selection without own performance (GBLUP_NoOP) or with own performance (GBLUP_OP). The three genetic models were a model with only additive effects (A), with additive and dominance effects (AD), or with additive, dominance and epistatic effects (ADE). Results are shown as averages of 20 replicates and the width of the lines represents the average plus and minus one standard error

In the first generation of selection, the accuracy of selection was always highest with genomic selection including own performance (GBLUP_OP) (Fig. 2). The accuracy was ~ 0.83 for models A and AD, and ~ 0.72 for model ADE. The lower accuracy for model ADE is a result of the lower narrow-sense heritability for this model. For all genetic models, the accuracy of the pedigree selection scenario with own performance (PBLUP_OP) in generation 1 was ~ 0.09 lower than with GBLUP_OP, the accuracy of genomic selection without own performance (GBLUP_NoOP) was ~ 0.13 lower than with GBLUP_OP, and the accuracy of MASS was ~ 0.21 lower than with GBLUP_OP. As expected, the accuracy of MASS was equal to the square root of the narrow-sense heritability.

Across generations, the accuracy of selection decreased for all scenarios. The decrease was largest in the first generations as a result of the Bulmer effect [72]. Thereafter, the decrease was slightly larger for the genomic selection scenarios (GBLUP_OP and GBLUP_NoOP) than for PBLUP_OP and MASS. As a result, differences in accuracy between the scenarios were smaller after 50 generations of selection than in the first generation. The accuracy decreased fastest under the ADE genetic model, especially for the genomic selection scenarios. Under this genetic model, the accuracies of PBLUP_OP, MASS and GBLUP_OP were similar after 50 generations of selection.

### Genetic gain

**Fig. 3:**
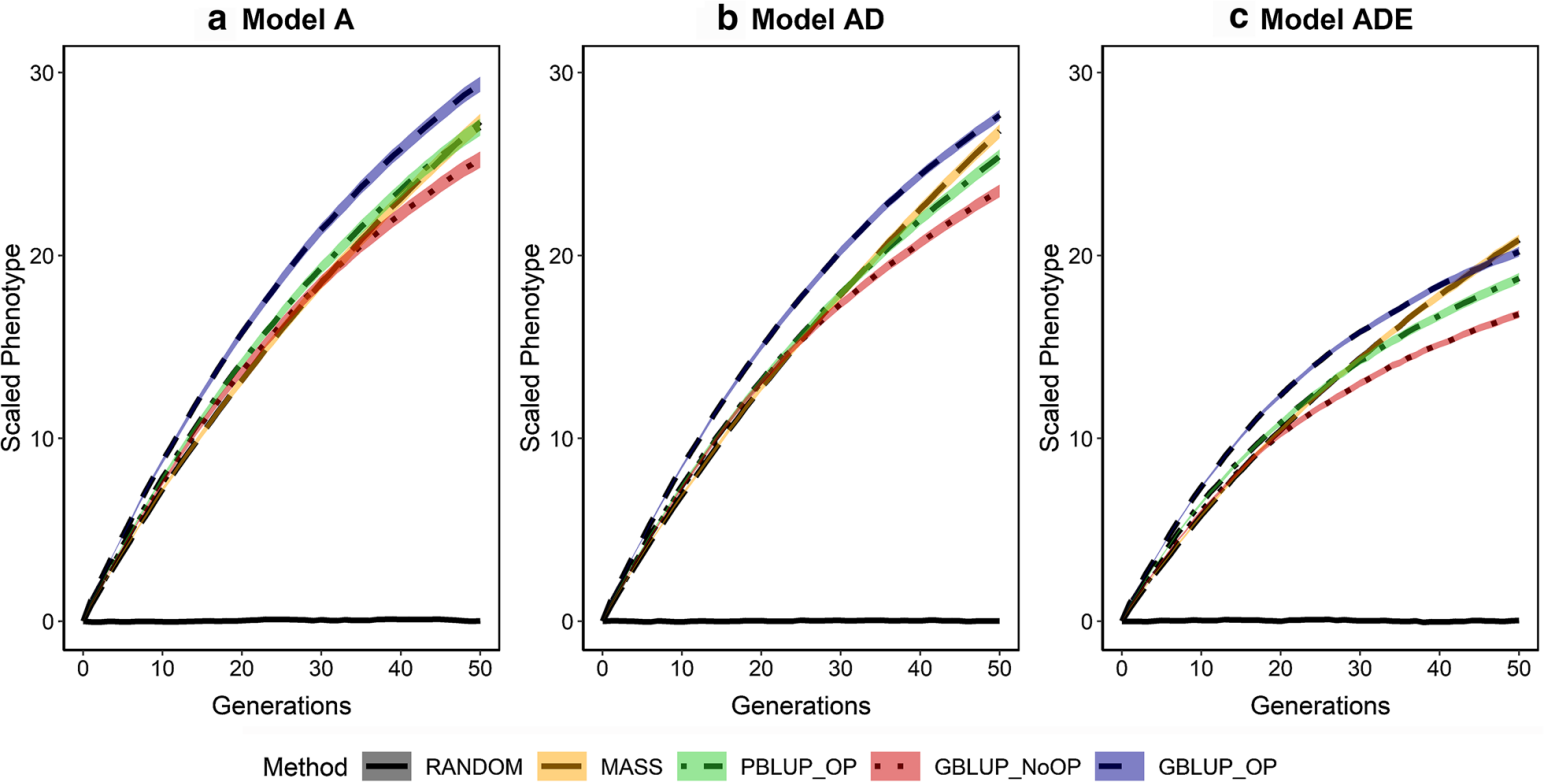
Phenotypic trends for the five selection methods and three genetic models. The phenotypic trend is scaled by the additive genetic standard deviation in the generation before selection in order to make the results comparable across the genetic models. The five selection methods were: RANDOM selection, MASS selection, PBLUP selection with own performance (PBLUP_OP), GBLUP selection without own performance (GBLUP_NoOP) or with own performance (GBLUP_OP). The three genetic models were a model with only additive effects (A), with additive and dominance effects (AD), or with additive, dominance and epistatic effects (ADE). Results are shown as averages of 20 replicates and the width of the lines represents the average plus and minus one standard error

Across generations, the average phenotypic value in the population was constant for the RANDOM scenario and increased with selection (Fig. 3). The rates of genetic gain in the first generations resembled the results for accuracy, with the highest values for GBLUP_OP, followed by PBLUP_OP, GBLUP_NoOP, and finally MASS, and smaller values when non-additive effects were present. The rate of genetic gain decreased over generations, but considerably less for MASS than for the other selection methods. Thus, after 50 generations of selection, cumulative genetic gain was greater for MASS than for PBLUP_OP and GBLUP_NoOP under all genetic models, and MASS also outperformed GBLUP_OP under model ADE.

### Additive genetic and genic variance

**Fig. 4:**
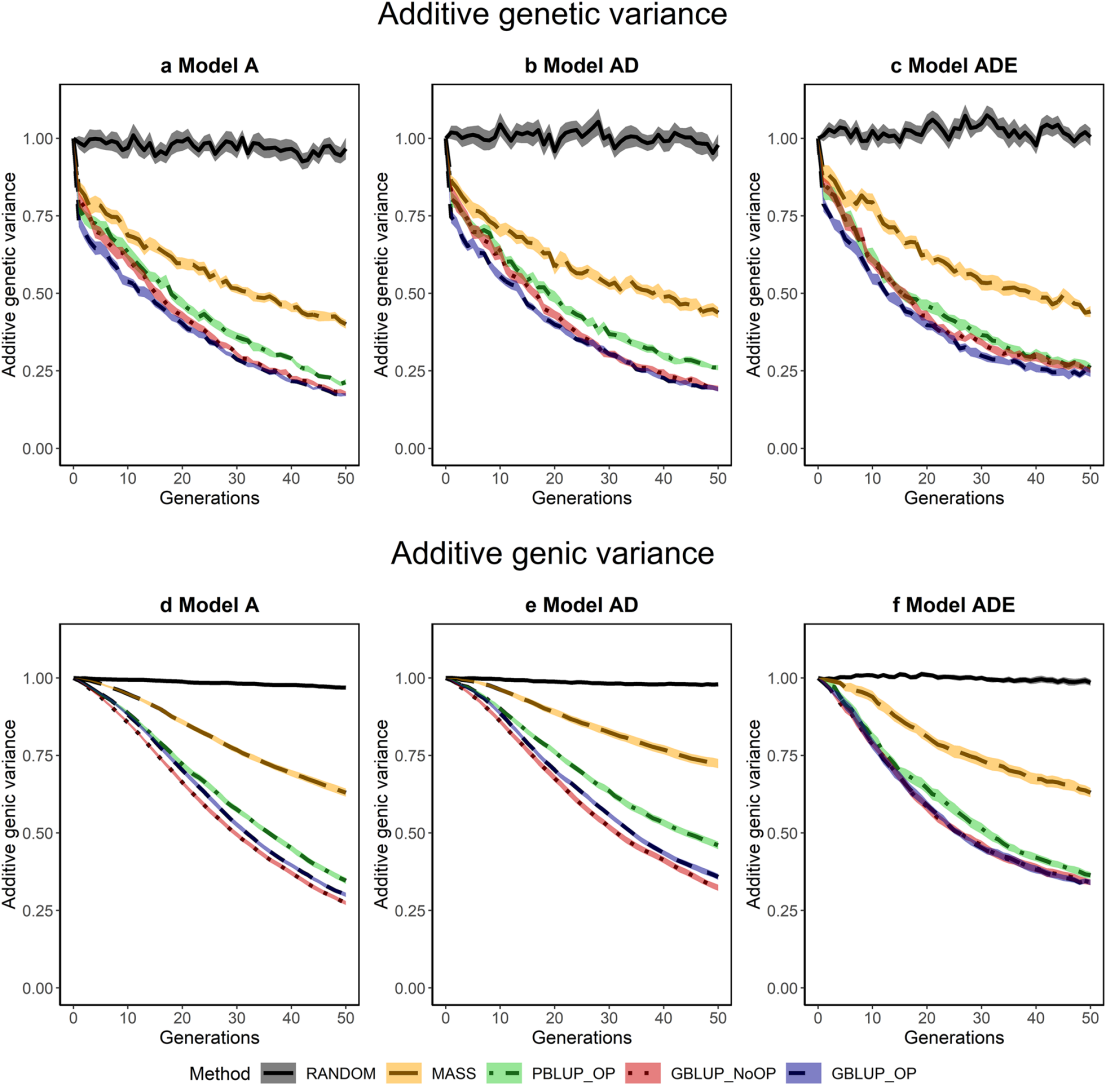
Additive genetic (**a**–**c**) and additive genic (**d**–**f**) variances across generations for the five selection methods and three genetic models. The trend is scaled by the additive genetic or additive genic variance in the generation before selection in order to make the results comparable across the genetic models. The five selection methods were: RANDOM selection, MASS selection, PBLUP selection with own performance (PBLUP_OP), GBLUP selection without own performance (GBLUP_NoOP) or with own performance (GBLUP_OP). The three genetic models were a model with only additive effects (A), with additive and dominance effects (AD), or with additive, dominance and epistatic effects (ADE). Results are shown as averages of 20 replicates and the width of the lines represents the average plus and minus one standard error

The additive genetic and genic variances were approximately constant for the RANDOM scenario and decreased with selection (Fig. 4). As expected, the largest drop in additive genetic variance was observed in the first generations of selection as a result of the Bulmer effect, similar to what was observed for the accuracy of selection; by more than 20% in the first three generations of selection. The total drop in genetic variance after 50 generations of selection was more or less similar for GBLUP_OP and GBLUP_NoOP, for which less than 20% of the initial genetic variance was maintained under genetic models A and AD. Under model ADE, more genetic variance was maintained (~ 24%) for GBLUP_OP and GBLUP_NoOP after 50 generations of selection. Only slightly more genetic variance (~ 25%) was maintained with PBLUP_OP, for which the loss in genetic variance was reasonably similar across the three genetic models. With MASS, the loss in genetic variance was considerably less, with ~ 40% of the variance maintained after 50 generations of selection.

The additive genic variance is not affected by transient effects such as the Bulmer effect [72]. Therefore, the loss in genic variance was smaller than the loss in genetic variance, especially in the first generations (Fig. 4). Except for this difference in the first generations, the trends in additive genic and genetic variance were very similar.

### Number of segregating causal loci

**Fig. 5:**
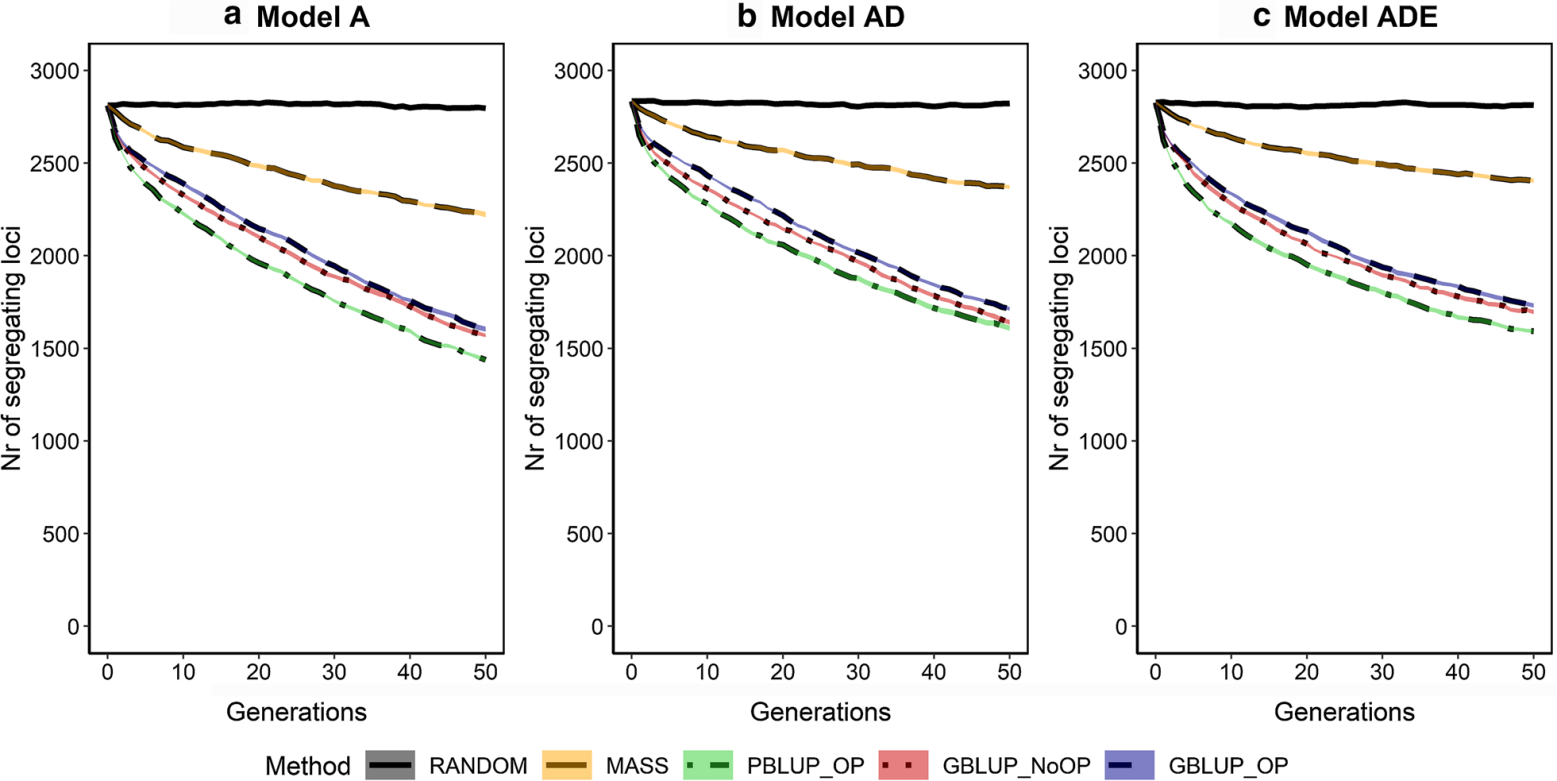
Numbers of segregating causal loci across generations for the five selection methods and three genetic models. The five selection methods were: RANDOM selection, MASS selection, PBLUP selection with own performance (PBLUP_OP), GBLUP selection without own performance (GBLUP_NoOP) or with own performance (GBLUP_OP). The three genetic models were a model with only additive effects (A), with additive and dominance effects (AD), or with additive, dominance and epistatic effects (ADE). Results are shown as averages of 20 replicates and the width of the lines represents the average plus and minus one standard error

The number of segregating causal loci decreased for the scenarios with selection (Fig. 5). For PBLUP_OP, the number of loci decreased fastest, with a reduction by almost 50% after 50 generations of selection. For GBLUP_OP and GBLUP_NoOP, the decrease was slightly smaller; 42% for GBLUP_OP and 40% for GBLUP_NoOP. For MASS, the decrease was substantially smaller, at only 20%. The loss in segregating loci was slightly smaller when non-additive effects were present. Interestingly, the number of segregating loci in generation 50 was smaller for PBLUP_OP than for GBLUP_OP and GBLUP_NoOP, while the additive genic variance was slightly larger for PBLUP_OP.

### Average minor allele frequency at segregating causal loci

**Fig. 6:**
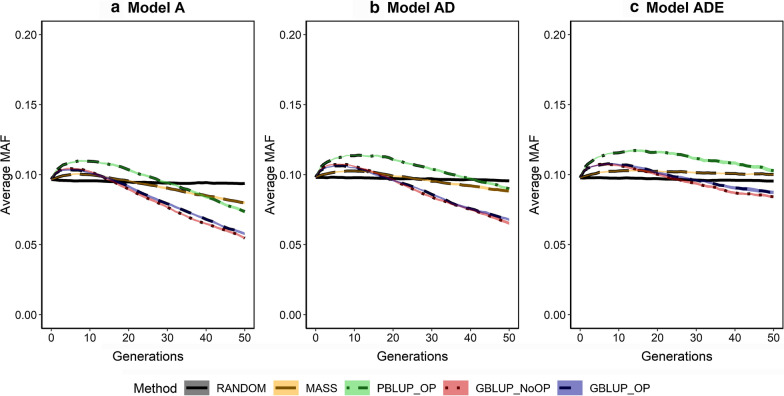
Average minor allele frequencies (MAF) of segregating causal loci across generations for the five selection methods and three genetic models. The five selection methods were: RANDOM selection, MASS selection, PBLUP selection with own performance (PBLUP_OP), GBLUP selection without own performance (GBLUP_NoOP) or with own performance (GBLUP_OP). The three genetic models were a model with only additive effects (A), with additive and dominance effects (AD), or with additive, dominance and epistatic effects (ADE). Results are shown as averages of 20 replicates and the width of the lines represents the average plus and minus one standard error

The additive genic variance depends on the number of segregating causal loci, as well as their MAF. In the first generations of selection, the average MAF of segregating loci increased, especially for PBLUP_OP (Fig. 6). Thereafter, the average MAF decreased and after 50 generations of selection, it was below its initial value, with the smallest values for the GBLUP scenarios. We found that the average MAF of PBLUP_OP and MASS after 50 generations of selection were slightly above the average MAF before selection, but only under the genetic model ADE. The impact of MASS on the average MAF of segregating loci was very small. The higher average MAF for PBLUP_OP can explain the larger additive genic variance for PBLUP_OP than for GBLUP_OP and GBLUP_NoOP, although PBLUP_OP resulted in a smaller number of segregating loci (Fig. 4 vs. Fig. 5).

### Accumulated heterozygosity

**Fig. 7:**
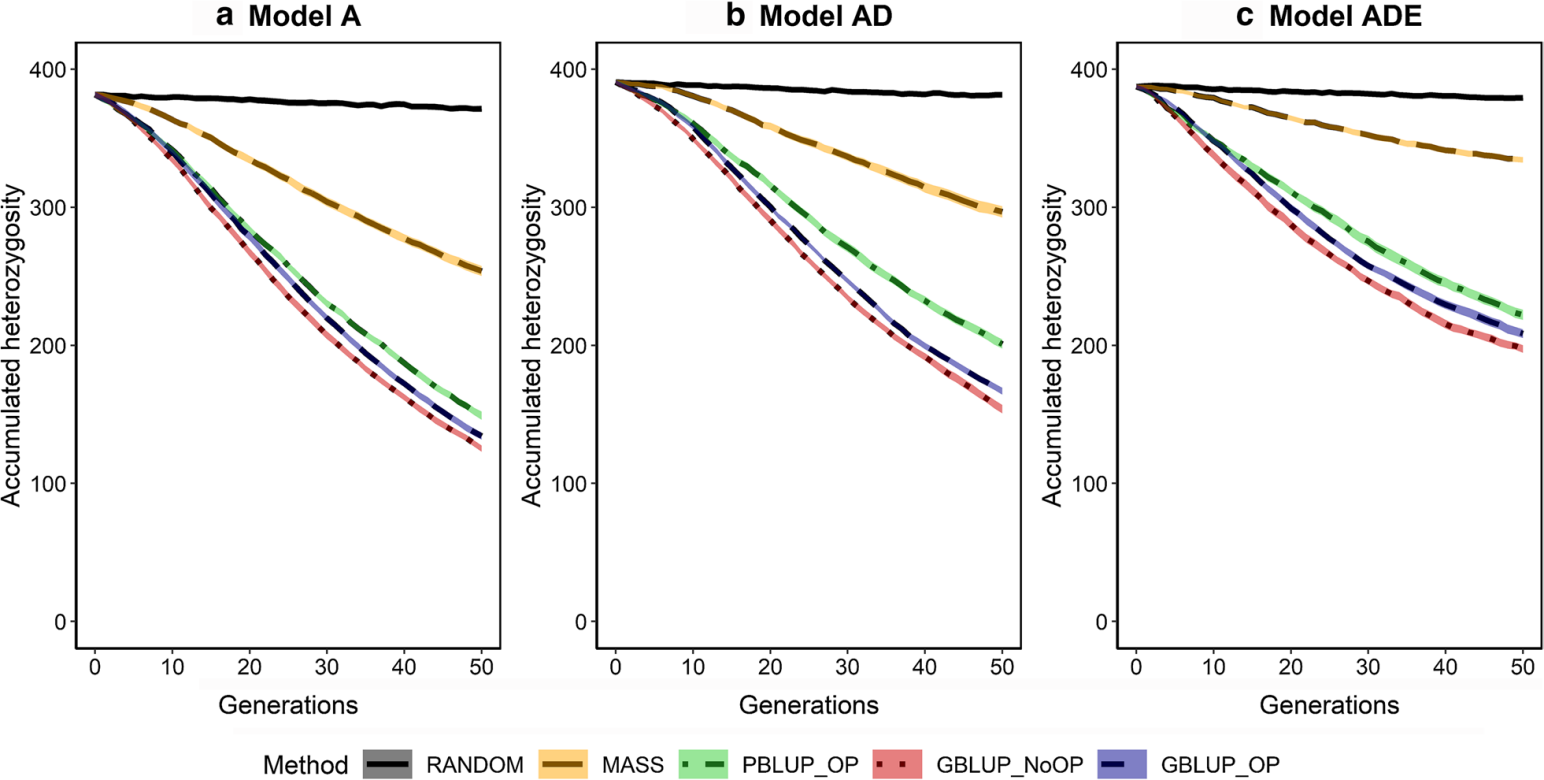
Average accumulated heterozygosity for segregating causal loci across generations for the five selection methods and three genetic models. The five selection methods were: RANDOM selection, MASS selection, PBLUP selection with own performance (PBLUP_OP), GBLUP selection without own performance (GBLUP_NoOP) or with own performance (GBLUP_OP). The three genetic models were a model with only additive effects (A), with additive and dominance effects (AD), or with additive, dominance and epistatic effects (ADE). Results are shown as averages of 20 replicates and the width of the lines represents the average plus and minus one standard error

In a random mating population, the accumulated heterozygosity depends on the number of segregating causal loci (Fig. 5), their average MAF (Fig. 6) and on the variation in MAF among those loci (see Additional file 2: Fig. S3 and Additional file 3). As expected, selection resulted in a decrease in the accumulated heterozygosity (Fig. 7). The reduction in accumulated heterozygosity was similar for GBLUP_OP and GBLUP_NoOP, slightly less for PBLUP_OP, and considerably less for MASS. Moreover, the accumulated heterozygosity decreased more slowly when non-additive effects were present. Thus, the decrease in heterozygosity was smaller for pedigree than for genomic selection, and depended on the genetic model.

### Changes in genetic architecture

**Fig. 8:**
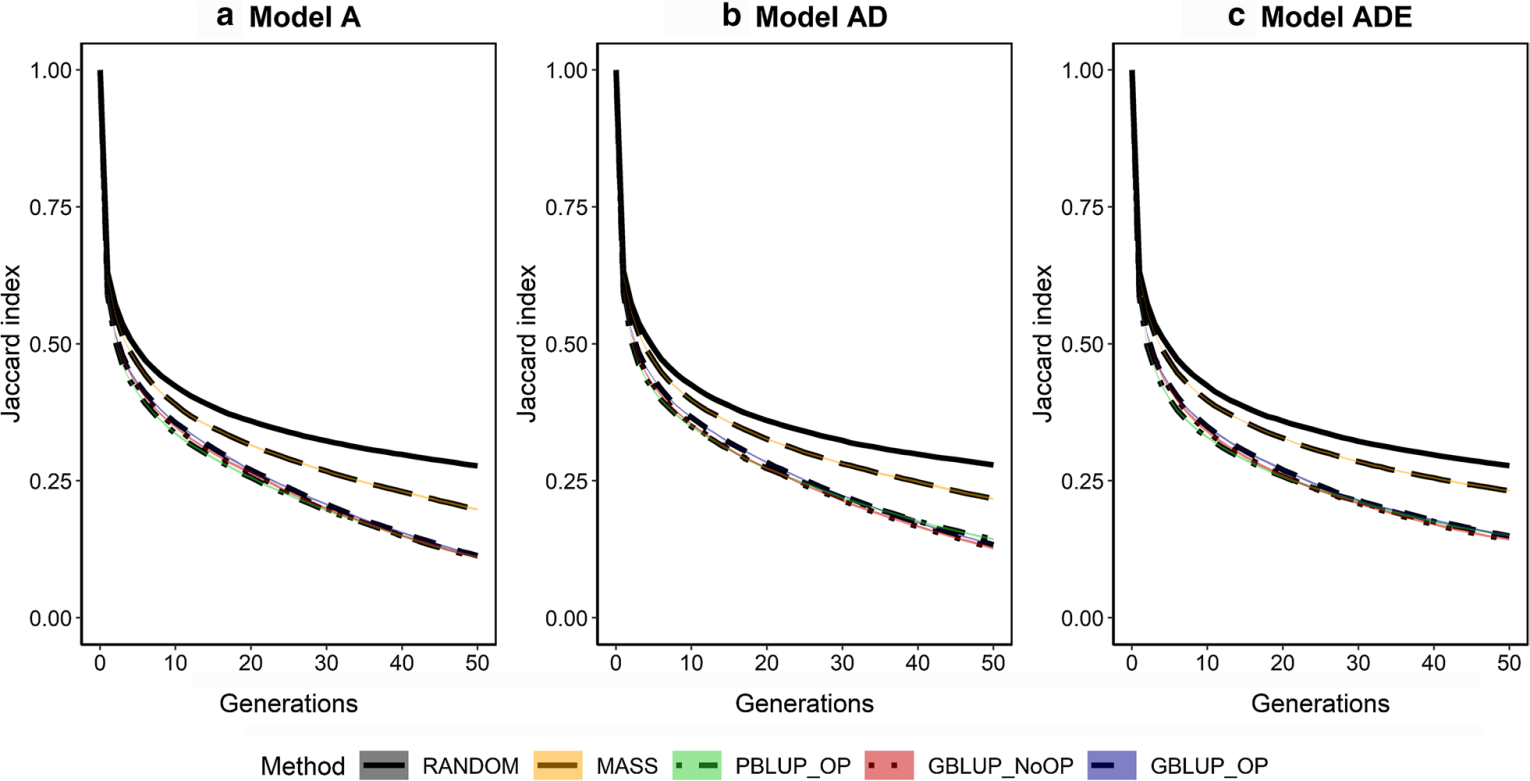
Change in the subset of segregating causal loci across generations for the five selection methods and three genetic models. The change in the subset of segregating causal loci is described by the Jaccard index. The five selection methods were: RANDOM selection, MASS selection, PBLUP selection with own performance (PBLUP_OP), GBLUP selection without own performance (GBLUP_NoOP) or with own performance (GBLUP_OP). The three genetic models were a model with only additive effects (A), with additive and dominance effects (AD), or with additive, dominance and epistatic effects (ADE). Results are shown as averages of 20 replicates and the width of the lines represents the average plus and minus one standard error

**Fig. 9:**
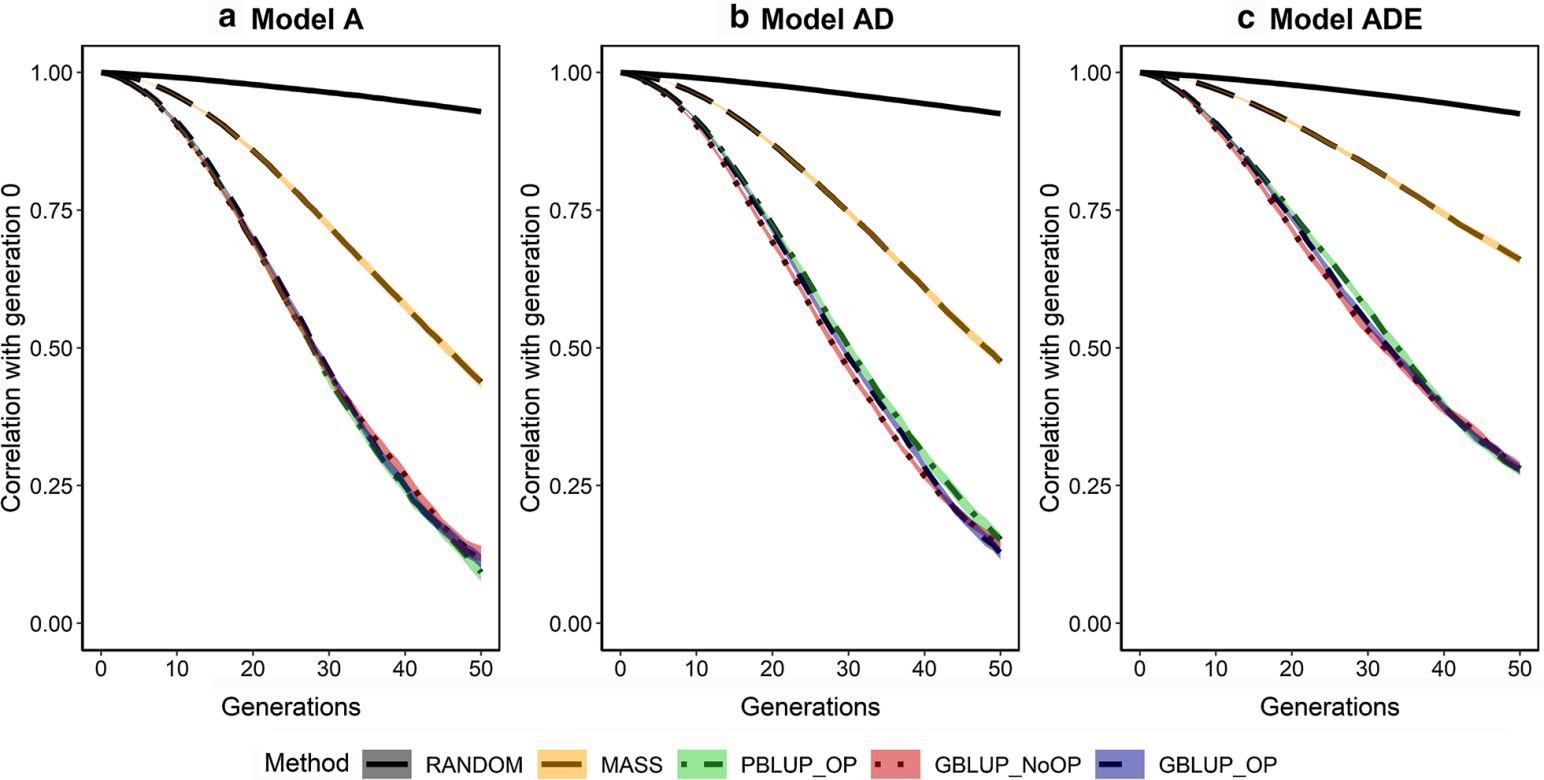
Change in the allele frequencies of segregating causal loci across generations for the five selection methods and three genetic models. The change in allele frequencies is represented by the correlation in allele frequencies between the generation of interest and the generation before selection (generation 0). The five selection methods were: RANDOM selection, MASS selection, PBLUP selection with own performance (PBLUP_OP), GBLUP selection without own performance (GBLUP_NoOP) or with own performance (GBLUP_OP). The three genetic models were a model with only additive effects (A), with additive and dominance effects (AD), or with additive, dominance and epistatic effects (ADE). Results are shown as averages of 20 replicates and the width of the lines represents the average plus and minus one standard error

**Fig. 10:**
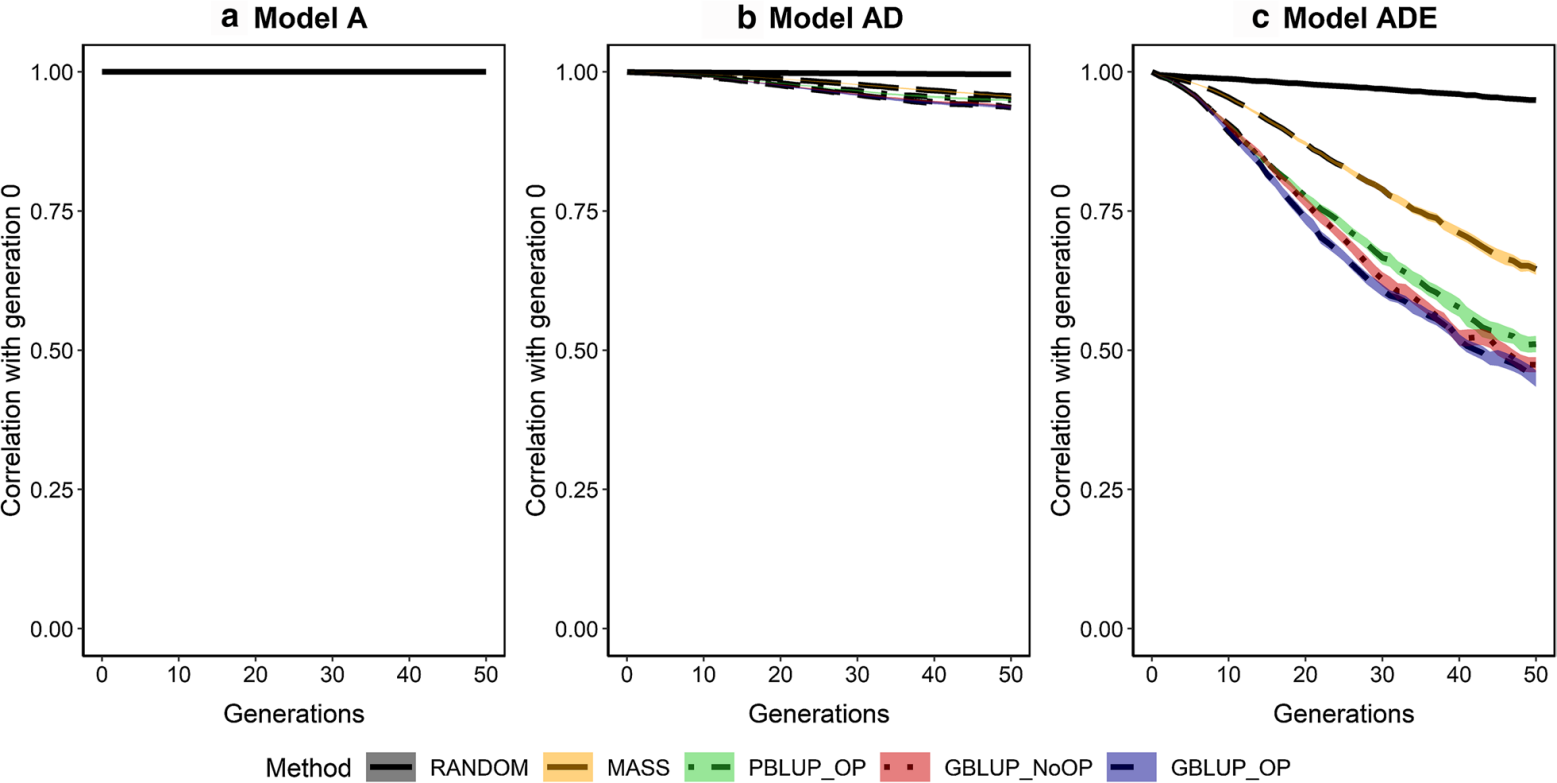
Change in the statistical additive effects of segregating causal loci across generations for the five selection methods and three genetic models. The change in statistical additive effects is represented by the correlation in the effects between the generation of interest and the generation before selection (generation 0). The five selection methods were: RANDOM selection, MASS selection, PBLUP selection with own performance (PBLUP_OP), GBLUP selection without own performance (GBLUP_NoOP) or with own performance (GBLUP_OP). The three genetic models were a model with only additive effects (A), with additive and dominance effects (AD), or with additive, dominance and epistatic effects (ADE). Results are shown as averages of 20 replicates and the width of the lines represents the average plus and minus one standard error

Across generations, the subset of causal loci underlying the trait (Fig. 8) and their allele frequencies (Fig. 9) and statistical additive effects (Fig. 10) changed. The change in the subset of loci was measured by the Jaccard index, which was substantial, especially in the first generation. Note that approximately 600 new mutations occurred in each generation, most of which were lost immediately. As a result, two consecutive generations already differed in nearly 1200 causal loci. The subset of loci that affected the trait changed considerably with drift (RANDOM) but the change was amplified by selection. After 50 generations, the average Jaccard index was ~ 0.27 for RANDOM, ~ 0.21 for MASS, and between 0.10 and 0.15 for PBLUP_OP, GBLUP_NoOP, and GBLUP_OP. The Jaccard index was slightly higher when non-additive genetic effects were present. Altogether, these results indicate that the subset of loci that affect the trait constantly changed across generations due to new mutations and drift, and that this change was amplified by selection.

Selection also strongly amplified the change in allele frequencies at causal loci compared to drift (Fig. 9) and (see Additional file 4: Figs. S1 to S15). Due to drift alone, the correlation between allele frequencies of causal loci that segregated in both generation 0 and generation 50 was ~ 0.93 (RANDOM). The change in allele frequencies as a result of selection was largest under model A, with a correlation between the allele frequencies in generations 0 and 50 of only ~ 0.10 for GBLUP_OP, GBLUP_NoOP, and PBLUP_OP, and of 0.44 for MASS. These correlations were slightly higher under model AD. When epistatic effects were also present, the change in allele frequencies was much smaller, and the correlation was ~ 0.28 after 50 generations of GBLUP_OP, GBLUP_NoOP, and PBLUP_OP, and 0.66 for MASS.

As a result of the change in allele frequencies, statistical additive effects of the loci changed when non-additive effects were present (Fig. 10) and (see Additional file 5: Figs. S1 to S10). These changes were quite limited when only additive and dominance effects were present, with an average correlation of 0.94 between the statistical additive effects in generations 0 and 50 for all selection methods. When epistatic effects were also present, this correlation was much lower, with an average correlation of 0.95 for RANDOM, 0.65 for MASS, 0.51 for PBLUP_OP, 0.47 for GBLUP_NoOP, and 0.45 for GBLUP_OP. Within 10 generations of selection based on GBLUP_OP, GBLUP_NoOP or PBLUP_OP, these correlations had already dropped to ~ 0.90.

## Discussion

We investigated the long-term effects of genomic selection on the rate of genetic gain, additive genetic variance, and the genetic architecture of quantitative traits. Results showed that, across 50 generations of genomic selection (GBLUP), the accuracy of selection, the rate of genetic gain, the amounts of additive genetic and genic variation, and the number of segregating causal loci decreased. The same trends were also observed for phenotypic (MASS) and pedigree (PBLUP) selection, but the reductions in these parameters were slightly smaller for PBLUP and considerably smaller for MASS. The main results of our study are summarized in Table 2, which also refers to the most likely mechanisms that underlie the results, which will be further discussed in the following sections.

**Table 2:** Summary of the long-term effects of genomic selection and associated mechanisms

Long-term effects of genomic selection on	Simulation results	Likely or proven mechanisms
Rate of genetic gain (Fig. 3)	Large drop in rate of genetic gain over generations	Loss in additive genetic variance and reduction in accuracy
	Epistasis increased the drop in rate of genetic gain over generations	Epistasis reduced the informativeness of previous generations for breeding value estimation, and increased the level of inbreeding depression
	Higher rate of genetic gain with genomic selection than with pedigree selection	Accuracy of breeding value estimation is higher with genomic selection than pedigree selection
Loss in additive genetic variance (Fig. 4)	First generations: large drop in additive genetic variance, smaller drop in additive genic variance	Bulmer effect, resulting in transient loss in additive genetic variance due to negative covariances between loci
	Later generations: large drop in additive genetic and genic variance	Reduction in the number of segregating loci, because of fixation of alleles due to selection and losing rare favorable alleles as a result of shrinking estimated effects of rare loci towards zero. Moreover, the average heterozygosity level reduced due to selection
	Epistasis reduced the loss in additive genetic variance	Epistasis resulted in fixing a lower number of loci, because the pressure and direction of selection at a locus can change over generations due to changing statistical additive effects
	Similar loss in additive genetic variance with genomic selection than with pedigree selection	Genomic selection maintained more segregating loci, but each of them with a lower MAF than pedigree selection, probably because pedigree selection results in a stronger family selection
Change in genetic architecture of traits (Figs. 8, 9, 10)	Large change in genetic architecture	Selection changes the allele frequencies of causal loci, thereby changing the subset of segregating causal loci and their statistical additive effects
	Epistasis reduced the change in subset of loci and allele frequencies, but increased the change in statistical additive effects	When epistasis was present, statistical additive effects of causal loci changed across generations, which lowered the change in allele frequency because the pressure and direction of selection at a locus changed across generations
	Subtle differences in change in genetic architecture between pedigree and genomic selection	Genomic selection focused more on a subset of genes that change rapidly, but the average change in allele frequency was similar for genomic and pedigree selection

### Genetic gain

The cumulative genetic gain after 50 generations of GBLUP_OP selection was 8 to 9% higher than with PBLUP_OP, and 16 to 20% higher than with GBLUP_NoOP, mainly as a result of a higher accuracy. Selection resulted in a decrease in the accuracy over generations, which is in agreement with previous research [41, 73]. The drop in accuracy was largest for the model with epistatic effects (as will be further explained later), especially for GBLUP_OP and GBLUP_NoOP. Therefore, the presence of epistatic effects resulted in a larger decrease in rate of genetic gain over generations.

The drop in accuracy over generations was always smaller for MASS than for the other selection methods. Together with the ability of MASS to maintain more genetic variation, this resulted in the highest cumulative genetic gain after 50 generations for MASS when epistasis was present, and almost the highest cumulative gain when epistasis was absent, which agrees with previous research [74, 75].

Based on the causal loci that were segregating after 50 generations of selection, we estimated the theoretical maximum genetic gain that could still be achieved when all these loci would become fixed for the favourable allele, using the statistical additive effects of generation 50 (see Additional file 6: Table S1). This theoretical maximum was highest for RANDOM, followed by MASS, and was on average 7.6% and 6.1% higher for GBLUP with or without own performance than for PBLUP with own performance. This suggests that GBLUP is more sustainable in terms of maintaining future genetic gain than PBLUP.

### Genetic variance

All selection methods resulted in a significant loss in genetic variance (Fig. 4). Part of this loss was transient and a result of the Bulmer effect [72]. The reasonably small difference between the genetic and genic variance indicates that this transient loss of genetic variance was limited (see Additional file 2: Fig. S4). Therefore, most of the loss in genetic variance was permanent and resulted from changes in allele frequencies.

Genic variance is a function of the number of segregating causal loci ($$n$$), their average heterozygosity $$\left( {\overline{{H_{E} }} } \right)$$, the average square of their statistical additive effects $$\left( {\overline{{\alpha^{2}}} } \right),
$$ and the covariance between their heterozygosity and $$\alpha^{2}$$ $$(Cov\left( {H_{E} ,\alpha^{2} } \right))$$ (see Additional file 3). Although the total loss in genic variance was comparable for GBLUP and PBLUP, PBLUP lost more segregating causal loci than GBLUP (see Additional file 6: Table S2). In contrast, the loss in average heterozygosity level at causal loci was greater for GBLUP than for PBLUP, likely because of stronger family selection with PBLUP, which agrees with its higher level of pedigree inbreeding than with GBLUP (see Additional file 6: Table S3).

Besides the drop in the number of segregating causal loci and their average heterozygosity, the genic variance slightly decreased over generations with GBLUP and PBLUP, as a result of a decrease in $\overline{{\alpha^{2} }}$ over generations (see Additional file 6: Table S2). This drop in $\overline{{\alpha^{2} }}$ is likely because loci with a larger statistical additive effect were more likely to become fixed over generations, which was stronger when epistasis was present. The covariance between $H_{E}$ and $\alpha^{2}$ was in general close to zero and contributed only little to the genic variance.

Compared to GBLUP and PBLUP, the loss in genic variance was much smaller for MASS. This was mostly because MASS maintained much more segregating loci, probably because MASS is better able to exploit and maintain rare favorable alleles than GBLUP and PBLUP [41, 76] and because selection pressure on loci is smaller with MASS, which reduces the loss of segregating loci as a result of hitchhiking [77].

The loss in genic variance was slightly smaller when non-additive effects were present. With non-additive effects, the statistical additive effects of loci depend on their allele frequencies [37, 78]. For some loci, the sign of the statistical additive effects even changed over generations when epistasis was present (see Additional file 5: Figs. S1 to S10), which changed the direction of selection on these loci and limited the number of loci that became fixed in the population. This resulted in a larger number of segregating loci (Fig. 5) and a higher level of heterozygosity (Fig. 7) after 50 generations of selection when non-additive effects were present (see Additional file 6: Table S2).

### Genetic architecture

Our initial plan was to quantify the change in genetic architecture by the additive genetic correlation between generations. However, this turned out to be very complex, because this correlation depends on the subset of individuals used. For example, the genetic correlation between generations 1 and 10 depends on whether it is estimated based on individuals from generation 1, from generation 10, or both [52]. Therefore, we decided to focus on three measures that reflect the underlying mechanisms, i.e. the changes in the subset of segregating causal loci, in their allele frequencies, and in their statistical additive effects.

Contrary to our expectations and to earlier results [40, 77], the average change in allele frequencies at segregating loci across generations was similar for GBLUP and PBLUP. The variance of the change in allele frequencies at loci was, however, larger for GBLUP than for PBLUP (see Additional file 6: Table S4). These results confirm that GBLUP focusses more on a subset of the genome that changes rapidly in allele frequencies, while PBLUP spreads the selection pressure more evenly across the genome [38, 39].

We hypothesized that the changes in allele frequencies could result in changes in true breeding values over generations when non-additive effects are present. Therefore, we estimated the correlation between true breeding values of individuals from generation 50 with the true breeding values of the same individuals for performance in generations 49 through 47, where true breeding values for a particular generation were calculated using the statistical additive effects of those generations. The correlation was always higher than 0.99 when only additive or additive and dominance effects were present, but substantially lower than 1 for PBLUP and GBLUP when epistasis was present (Table 3; ~ 0.95 with generation 49, ~ 0.91 with generation 48, and ~ 0.87 with generation 47), with slightly lower values for GBLUP. This indicates that the correlation of true breeding values between generations decreased rapidly, although the correlation of statistical additive effects was very high between subsequent generations (> 0.99, Fig. 10), which is probably because statistical additive effects changed more rapidly for loci that had a high MAF or a large effect (see Additional file 5: Figs. S1 to S10). This phenomenon drastically decreased the informativeness of previous generations for prediction of breeding values, which reduced the accuracy of selection. Thus, recent generations of reference populations are more useful for genomic prediction, not only because they are more closely related to the selection candidates [68, 79, 80], but also because their genetic architecture is more similar to that of the selection candidates. This might explain why it is sometimes beneficial to remove earlier generations from the reference populations [81, 82]. Moreover, the correlation between statistical additive effects cannot be used to investigate the informativeness of previous generations, because a high correlation between statistical additive effects does not necessarily imply that the correlation between true breeding values is high.

**Table 3:** Correlation of true breeding values (TBV) of individuals from generation 50 between performance in generation 50 and each of the three previous generations^a^ for three genetic models (A, AD, ADE) and five selection methods^b^

	Correlation in TBV of generation 50 with		
	Generation 49	Generation 48	Generation 47
Model A			
RANDOM	1.00 (0.000)	1.00 (0.000)	1.00 (0.000)
MASS	1.00 (0.000)	1.00 (0.000)	1.00 (0.000)
PBLUP	1.00 (0.000)	1.00 (0.000)	1.00 (0.000)
GBLUP_NoOP	1.00 (0.000)	1.00 (0.000)	1.00 (0.000)
GBLUP_OP	1.00 (0.000)	1.00 (0.000)	1.00 (0.000)
Model AD			
RANDOM	1.00 (0.000)	1.00 (0.000)	1.00 (0.000)
MASS	1.00 (0.000)	1.00 (0.000)	1.00 (0.000)
PBLUP	1.00 (0.000)	0.99 (0.001)	0.99 (0.001)
GBLUP_NoOP	1.00 (0.000)	0.99 (0.001)	0.99 (0.001)
GBLUP_OP	1.00 (0.000)	0.99 (0.000)	0.99 (0.001)
Model ADE			
RANDOM	0.99 (0.000)	0.99 (0.000)	0.99 (0.000)
MASS	0.98 (0.001)	0.98 (0.002)	0.97 (0.003)
PBLUP	0.95 (0.003)	0.92 (0.006)	0.90 (0.007)
GBLUP_NoOP	0.96 (0.004)	0.91 (0.009)	0.86 (0.012)
GBLUP_OP	0.95 (0.003)	0.91 (0.006)	0.87 (0.010)

^a^True breeding values were estimated for individuals from generation 50, for performance in generation 50 and the three previous generations based on the genotypes of the individuals in generation 50 and the statistical additive effects of each of those generations

^b^Results are shown as averages across the 20 replicates with their corresponding standard errors of the mean between brackets

### Non-additive effects

A large part of the functional dominance and epistatic effects at causal loci can be converted into additive genetic variance [26, 29, 30, 83]. Due to this conversion, it is difficult to draw conclusions about the magnitude of the functional epistasis in a population based on the level of epistatic variance [84, 85]. In our study, for example, the epistatic variance only explained 5% of the genetic variance, although almost 50% of the variation in the total genetic value was generated by functional epistatic effects.

**Fig. 11:**
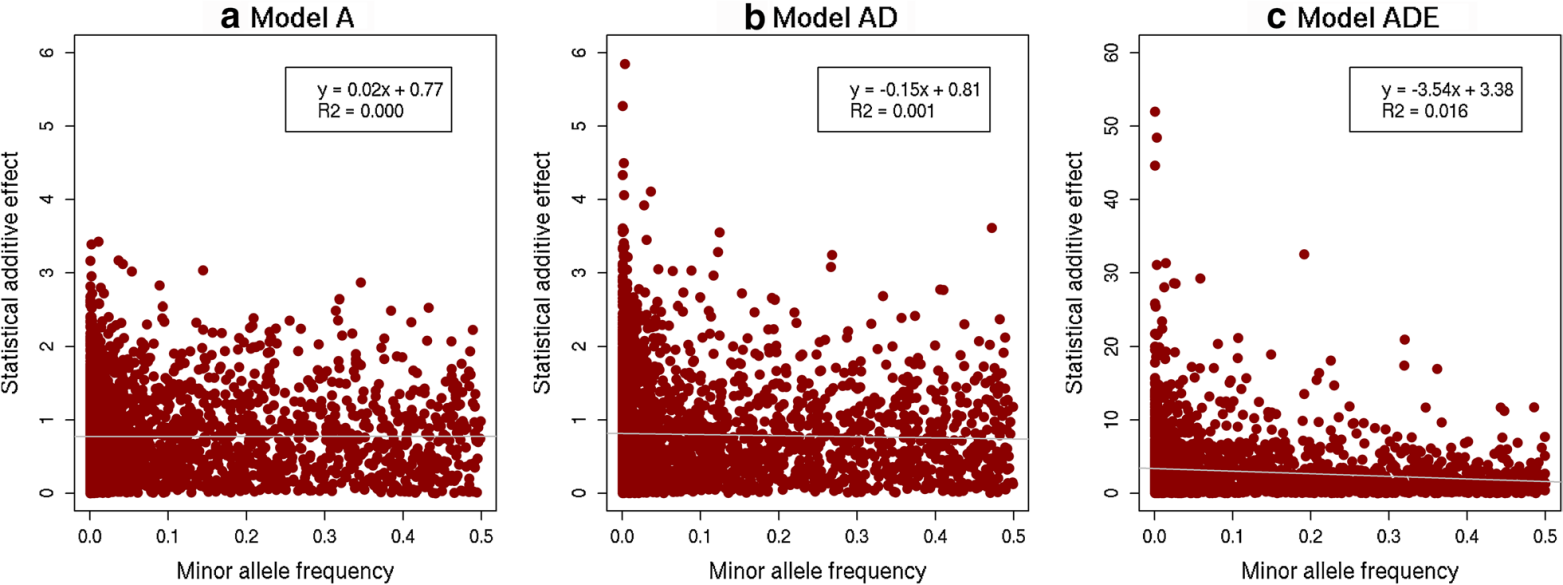
Correlation between the absolute statistical additive effect and the minor allele frequency at causal loci for the three genetic models. The three genetic models were a model with only additive effects (A), with additive and dominance effects (AD), or with additive, dominance and epistatic effects (ADE)

The conversion of non-additive effects into statistical additive effects depends on allele frequencies, with a larger proportion of the non-additive effects converted into statistical additive effects when allele frequencies are closer to 0 or 1 [27–29]. As a result, the MAF of a locus was negatively correlated with the absolute value of its statistical additive effect in our simulations, already before selection, although functional effects were simulated independently of allele frequencies (Fig. 11). A negative correlation between MAF and the size of the estimated additive effects of loci is often observed in empirical studies [86–88], and our results show that the presence of non-additive effects can contribute to explaining this finding and that it is not necessarily a result of selection.

Little is known about the structure and network of epistatic interactions. We only simulated pairwise interactions and mimicked the genetic interaction network observed in yeast, with many loci that have few interactions and few loci that have many interactions. Although studied in less detail than in yeast, similar interaction networks have been reported for *C. elegans* [89], *drosophila* [31] and mice [90], and are also found for proteins [35], which led Boone et al*.* [36] and Mackay [37] to argue that it is likely that the interaction network between genetic loci is similar in other species such as livestock and humans. Thus, we used the knowledge that is available on interaction effects from yeast [34] to make the simulations as realistic as possible. However, we still had to make simplifications such as only including pair-wise interactions and independence of interaction effects between pairs. We expect that the trend in genetic gain, genetic variance, and genetic architecture for the three genetic models is likely correct because it follows expectations, however, the magnitude of the differences between models and selection methods could be affected by those simplifications.

### Genetic evaluation methods

To estimate genomic relationships, we used the allele frequencies for the individuals that were included in the genomic relationship matrix, i.e. the selection candidates and the previous three generations. In an additive analysis model, the use of different allele frequencies affects the estimates of the variance components but not the ranking of the estimated breeding values [91, 92] and, therefore, not the response to selection. The allele frequencies of the markers followed a uniform distribution, as is common for loci that are included on most commercial marker chips [45–47], which is different from the distribution of the causal loci, which was U-shaped. This difference could result in a slight bias in the estimated variance components and breeding values for the GBLUP scenarios [93, 94], which could have had a slightly negative effect on the long-term response for the GBLUP scenarios.

The models used in our study to estimate breeding values included only an additive effect. The availability of genomic data enables inclusion of non-additive effects in the breeding value estimation model [95–97]. We tested the benefit of including a dominance effect in the GBLUP model with own performance for the genetic models AD and ADE. With the AD genetic model, inclusion of a dominance effect in the GBLUP model had a negligible effect on genetic gain and loss of genetic variance (see Additional file 2: Figs. S5 and S6). With the ADE genetic model, inclusion of a dominance effect in GBLUP resulted in slightly higher long-term response to selection, which became apparent after about 20 generations. This is likely related to the much larger dominance variance in the ADE model compared to the AD model, which probably makes it more difficult for the random litter effect to capture all dominance genetic variance, resulting in a slight bias in estimated breeding values when a dominance effect is not included, which accumulates over generations. With the ADE genetic model, the cumulative genetic gain for the GBLUP model with dominance effects was comparable to MASS selection after 50 generations of selection. This result shows that inclusion of a dominance effect in the breeding value estimation model can be beneficial in the long-term when non-additive effects are large.

### Relevance for breeding programs

Our results show a larger loss in genetic variance than generally observed in actual populations, which is often observed in simulation studies e.g. [41, 43, 74, 76]. The loss in genetic variance was mainly due to a reduction in the number of segregating causal loci and in the mean heterozygosity at these loci. These results suggest that the average change in allele frequency was larger in our simulations than in actual populations. Moreover, the large differences between the RANDOM scenario and the selected scenarios suggest that the large changes in allele frequencies were mainly due to selection, rather than drift. The change in allele frequency at a locus due to selection depends on the proportion of genetic variance explained by the locus and on the level of linkage disequilibrium among loci that are under selection. Hence, in our simulations, a typical causal locus may have explained more variation than in actual populations, due to either a larger effect or a higher MAF, and the smaller genome of roughly 1/3 of the size of a typical livestock genome may have increased the effect of linkage. At present, we have insufficient knowledge of the number of loci and the joint distribution of the allele effects and allele frequencies in livestock populations to draw strong conclusions. It is also difficult to predict whether the larger loss in genetic variance than observed in actual populations affects the comparison of the selection methods. To be more in line with reality, a larger genome with more causal loci and lower MAF could be simulated, resulting in a less severe reduction in genetic variance. However, an agreement of the trend in genetic variance between simulations and actual populations still does not prove that the changes in genetic architecture observed in simulation match those of actual populations.

In our simulations, MASS outperformed GBLUP after 50 generations when both dominance and epistatic effects were present. Note, however, that we compared selection responses per generation, while the generation interval may differ between selection methods. Especially for GBLUP without own performance, the generation interval can be substantially reduced in some livestock populations, such as dairy cattle [9, 10]. Moreover, commercial breeding programs typically control the rate of inbreeding, for example by optimal contribution selection [98–101], which limits the loss in long-term genetic gain.

The focus of this study was not on optimization of breeding programs for long-term genetic gain, which is not realistic in practice because of competition between breeding companies, and optimization of long-term gain requires sacrificing short-term gain. Instead, our focus was on the long-term consequences of genomic selection compared to selection strategies that have a longer history in livestock populations, such as MASS and PBLUP, which have been proven to be sustainable for a relatively large number of generations.

## Conclusions

Our results show that short-term response was always greatest with GBLUP, while long-term response was greater for MASS than for GBLUP when epistasis was present, and was always greater for MASS than for PBLUP. This was mainly the result of a much larger loss in genetic variance and number of segregating loci with GBLUP and PBLUP than with MASS. The genetic gain of PBLUP with own performance records was always in between that of GBLUP with and without own performance records. GBLUP and PBLUP showed a similar loss in genetic variance, but the underlying mechanism was different with GBLUP maintaining more loci but with a lower MAF than PBLUP. The maximum genetic gain that could still be obtained after 50 generations was higher for GBLUP selection than for PBLUP, which suggests that GBLUP maintains long-term genetic gain better than PBLUP. Changes in the genetic architecture of the trait, i.e. Jaccard index of segregating causal loci, correlation in allele frequencies, and correlations in statistical additive effects across generations, were strongly amplified by selection but, in contrast to our hypothesis, comparable for GBLUP and PBLUP. Non-additive effects were relatively unimportant in the short-term but had a substantial impact on the accuracy and genetic gain when multiple generations were included in the reference population and selection was accurate.

## Supplementary Information

**Additional file 1.** Programs and seeds to simulate data. This file contains the QMSim input file, Fortran programs and seeds used to select the markers and causal loci, to simulate functional effects and genotypes and phenotypic values of new generations, and the interaction matrix used to simulate epistatic effects.

**Additional file 2: Figure S1.** Allele frequency distribution of segregating causal loci over 50 generations without selection. **Figure S2.** Extent of LD (*r*^2^) in the simulated population before selection as a function of distance in one random replicate. **Figure S3.** Trend in the variation in minor allele frequency (MAF) of segregating causal loci for the five selection methods and three genetic models. The five selection methods were: RANDOM selection, MASS selection, PBLUP selection with own performance (PBLUP_OP), GBLUP selection without own performance (GBLUP_NoOP) or with own performance (GBLUP_OP). The three genetic models were a model with only additive effects (A), with additive and dominance effects (AD), or with additive, dominance and epistatic effects (ADE). Results are shown as averages of 20 replicates and the width of the lines represents the average plus and minus one standard error. **Figure S4.** The difference between additive genic and additive genetic variance which represents a transient loss in genetic variance for the five selection methods and three genetic models. The five selection methods were: RANDOM selection, MASS selection, PBLUP selection with own performance (PBLUP_OP), GBLUP selection without own performance (GBLUP_NoOP) or with own performance (GBLUP_OP). The three genetic models were a model with only additive effects (A), with additive and dominance effects (AD), or with additive, dominance and epistatic effects (ADE). Results are shown as averages of 20 replicates and the width of the lines represents the average plus and minus one standard error. **Figure S5.** Phenotypic trend for the GBLUP model with own performance records and with and without a dominance effect for the genetic models with non-additive effects. The phenotypic trend is scaled by the additive genetic standard deviation in the generation before selection in order to make the results comparable across the genetic models. The two genetic models were a model with additive and dominance effects (AD), or with additive, dominance and epistatic effects (ADE). Results are shown as averages of 20 replicates and the width of the lines represents the average plus and minus one standard error. **Figure S6.** Trend in additive genetic (A, B) and additive genic (C, D) variance for the GBLUP model with own performance and with and without a dominance effect for the genetic models with non-additive effects. The trend is scaled by the additive genetic or additive genic variance in the generation before selection in order to make the results comparable across the genetic models. The two genetic models were a model with additive and dominance effects (AD), or with additive, dominance and epistatic effects (ADE). Results are shown as averages of 20 replicates and the width of the lines represents the average plus and minus one standard error.

**Additional file 3.** Decomposition of additive genetic variance. This file provides a theoretical decomposition of the additive genetic variance.

**Additional file 4: Figure S1.** Scatterplot of allele frequencies of all causal variants in different generations for the genetic model with additive effects (Model A) under RANDOM selection. **Figure S2.** Scatterplot of allele frequencies of all causal variants in different generations for the genetic model with additive effects (Model A) under MASS selection. **Figure S3.** Scatterplot of allele frequencies of all causal variants in different generations for the genetic model with additive effects (Model A) under PBLUP selection with own performance (PBLUP_OP). **Figure S4.** Scatterplot of allele frequencies of all causal variants in different generations for the genetic model with additive effects (Model A) under GBLUP selection without own performance (GBLUP_NoOP). **Figure S5.** Scatterplot of allele frequencies of all causal variants in different generations for the genetic model with additive effects (Model A) under GBLUP selection with own performance (GBLUP_OP). **Figure S6.** Scatterplot of allele frequencies of all causal variants in different generations for the genetic model with additive and dominance effects (Model AD) under RANDOM selection. **Figure S7.** Scatterplot of allele frequencies of all causal variants in different generations for the genetic model with additive and dominance effects (Model AD) under MASS selection. **Figure S8.** Scatterplot of allele frequencies of all causal variants in different generations for the genetic model with additive and dominance effects (Model AD) under PBLUP selection with own performance (PBLUP_OP). **Figure S9.** Scatterplot of allele frequencies of all causal variants in different generations for the genetic model with additive and dominance effects (Model AD) under GBLUP selection without own performance (GBLUP_NoOP). **Figure S10.** Scatterplot of allele frequencies of all causal variants in different generations for the genetic model with additive and dominance effects (Model AD) under GBLUP selection with own performance (GBLUP_OP). **Figure S11.** Scatterplot of allele frequencies of all causal variants in different generations for the genetic model with additive, dominance and epistatic effects (Model ADE) under RANDOM selection. **Figure S12.** Scatterplot of allele frequencies of all causal variants in different generations for the genetic model with additive, dominance and epistatic effects (Model ADE) under MASS selection. **Figure S13.** Scatterplot of allele frequencies of all causal variants in different generations for the genetic model with additive, dominance and epistatic effects (Model ADE) under PBLUP selection with own performance (PBLUP_OP). **Figure S14.** Scatterplot of allele frequencies of all causal variants in different generations for the genetic model with additive, dominance and epistatic effects (Model ADE) under GBLUP selection without own performance (GBLUP_NoOP). **Figure S15.** Scatterplot of allele frequencies of all causal variants in different generations for the genetic model with additive, dominance and epistatic effects (Model ADE) under GBLUP selection with own performance (GBLUP_OP).

**Additional file 5: Figure S1.** Scatterplot of statistical additive effects in different generations for the genetic model with additive and dominance effects (Model AD) under RANDOM selection. **Figure S2.** Scatterplot of statistical additive effects in different generations for the genetic model with additive and dominance effects (Model AD) under MASS selection. **Figure S3.** Scatterplot of statistical additive effects in different generations for the genetic model with additive and dominance effects (Model AD) under PBLUP selection with own performance (PBLUP_OP). **Figure S4.** Scatterplot of statistical additive effects in different generations for the genetic model with additive and dominance effects (Model AD) under GBLUP selection without own performance (GBLUP_NoOP). **Figure S5.** Scatterplot of statistical additive effects in different generations for the genetic model with additive and dominance effects (Model AD) under GBLUP selection with own performance (GBLUP_OP). **Figure S6.** Scatterplot of statistical additive effects in different generations for the genetic model with additive, dominance and epistatic effects (Model ADE) under RANDOM selection. **Figure S7.** Scatterplot of statistical additive effects in different generations for the genetic model with additive, dominance and epistatic effects (Model ADE) under MASS selection. **Figure S8.** Scatterplot of statistical additive effects in different generations for the genetic model with additive, dominance and epistatic effects (Model ADE) under PBLUP selection with own performance (PBLUP_OP). **Figure S9.** Scatterplot of statistical additive effects in different generations for the genetic model with additive, dominance and epistatic effects (Model ADE) under GBLUP selection without own performance (GBLUP_NoOP). **Figure S10.** Scatterplot of statistical additive effects in different generations for the genetic model with additive, dominance and epistatic effects (Model ADE) under GBLUP selection with own performance (GBLUP_OP).

**Additional file 6: Table S1.** Maximum genetic gain^1^ that is still possible after 50 generations of selection for the five selection methods and three genetic models^2^. The five selection methods were: RANDOM selection, MASS selection, PBLUP selection with own performance (PBLUP_OP), GBLUP selection without own performance (GBLUP_NoOP) or with own performance (GBLUP_OP). The three genetic models were a model with only additive effects (A), with additive and dominance effects (AD), or with additive, dominance and epistatic effects (ADE). ^1^The maximum genetic gain in generation 50 is estimated as the genetic gain when all loci would be fixed for the favourable allele, using the statistical additive effects of generation 50 and neglecting mutations. ^2^Results are shown as averages across the 20 replicates with their corresponding standard errors of the mean between brackets. **Table S2.** Percentual change in the components of the genetic variance after 10 and 50 generations of selection for the five selection methods and three genetic models^1^. The five selection methods were: RANDOM selection, MASS selection, PBLUP selection with own performance (PBLUP_OP), GBLUP selection without own performance (GBLUP_NoOP) or with own performance (GBLUP_OP). The three genetic models were a model with only additive effects (A), with additive and dominance effects (AD), or with additive, dominance and epistatic effects (ADE). ^1^Results are shown as averages of 20 replicates with their corresponding standard errors of the mean between brackets. Increases in the value of a component are represented in bold. **Table S3.** Average pedigree inbreeding coefficient after 50 generations of selection for the five selection methods and three genetic models^1^. The five selection methods were: RANDOM selection, MASS selection, PBLUP selection with own performance (PBLUP_OP), GBLUP selection without own performance (GBLUP_NoOP) or with own performance (GBLUP_OP). The three genetic models were a model with only additive effects (A), with additive and dominance effects (AD), or with additive, dominance and epistatic effects (ADE). ^1^Results are shown as averages of 20 replicates with their corresponding standard errors of the mean between brackets. **Table S4.** Average and variance of change in allele frequency of causal loci^1^ across 50 generations of selection for the five selection methods and three genetic models^2^. The five selection methods were: RANDOM selection, MASS selection, PBLUP selection with own performance (PBLUP_OP), GBLUP selection without own performance (GBLUP_NoOP) or with own performance (GBLUP_OP). The three genetic models were a model with only additive effects (A), with additive and dominance effects (AD), or with additive, dominance and epistatic effects (ADE). ^1^Causal loci included only the causal loci segregating in generation 0. ^2^Results are shown as averages of 20 replicates with their corresponding standard errors of the mean between brackets.

## Data Availability

All scripts used to generate the data during this study are included in Additional file 1. This file contains the QMSim input file, Fortran programs and seeds used to select the markers and causal loci, to simulate functional effects and genotypes and phenotypic values of new generations, and the interaction matrix used to simulate epistatic effects.
