# Supplementary material for: The long-term effects of genomic selection: 1. Response to selection, additive genetic variance, and genetic architecture
Source: Genet Sel Evol. 2022 Mar 7;54:19. doi: 10.1186/s12711-022-00709-7 (PMC8900405; doi:10.1186/s12711-022-00709-7)
Supplement: Supplementary file 2 — Additional file 2: Figure S1. Allele frequency distribution of segregating causal loci over 50 generations without selection. Figure S2. Extent of LD (r2) in the simulated population before selection as a function of distance in one random replicate. Figure S3. Trend in the variation in minor allele frequency (MAF) of segregating causal loci for the five selection methods and three genetic models. The five selection methods were: RANDOM selection, MASS selection, PBLUP selection with own performance (PBLUP_OP), GBLUP selection without own performance (GBLUP_NoOP) or with own performance (GBLUP_OP). The three genetic models were a model with only additive effects (A), with additive and dominance effects (AD), or with additive, dominance and epistatic effects (ADE). Results are shown as averages of 20 replicates and the width of the lines represents the average plus and minus one standard error. Figure S4. The difference between additive genic and additive genetic variance which represents a transient loss in genetic variance for the five selection methods and three genetic models. The five selection methods were: RANDOM selection, MASS selection, PBLUP selection with own performance (PBLUP_OP), GBLUP selection without own performance (GBLUP_NoOP) or with own performance (GBLUP_OP). The three genetic models were a model with only additive effects (A), with additive and dominance effects (AD), or with additive, dominance and epistatic effects (ADE). Results are shown as averages of 20 replicates and the width of the lines represents the average plus and minus one standard error. Figure S5. Phenotypic trend for the GBLUP model with own performance records and with and without a dominance effect for the genetic models with non-additive effects. The phenotypic trend is scaled by the additive genetic standard deviation in the generation before selection in order to make the results comparable across the genetic models. The two genetic models were a model with additive and [file 12711_2022_709_MOESM2_ESM.docx]

**Additional file 2: Additional figures**


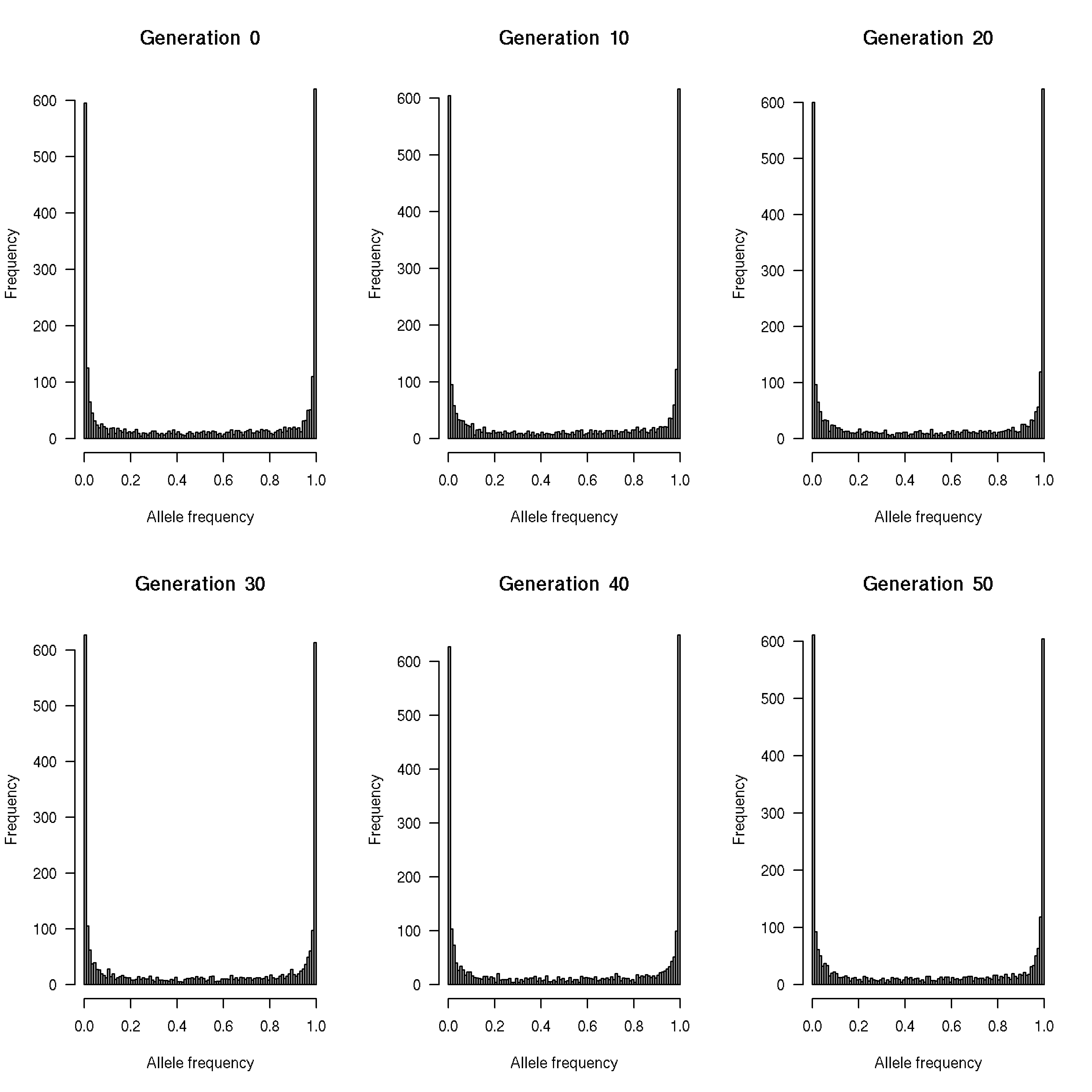


**FIGURE S1**

Allele frequency distribution of segregating causal loci over 50 generations without selection.


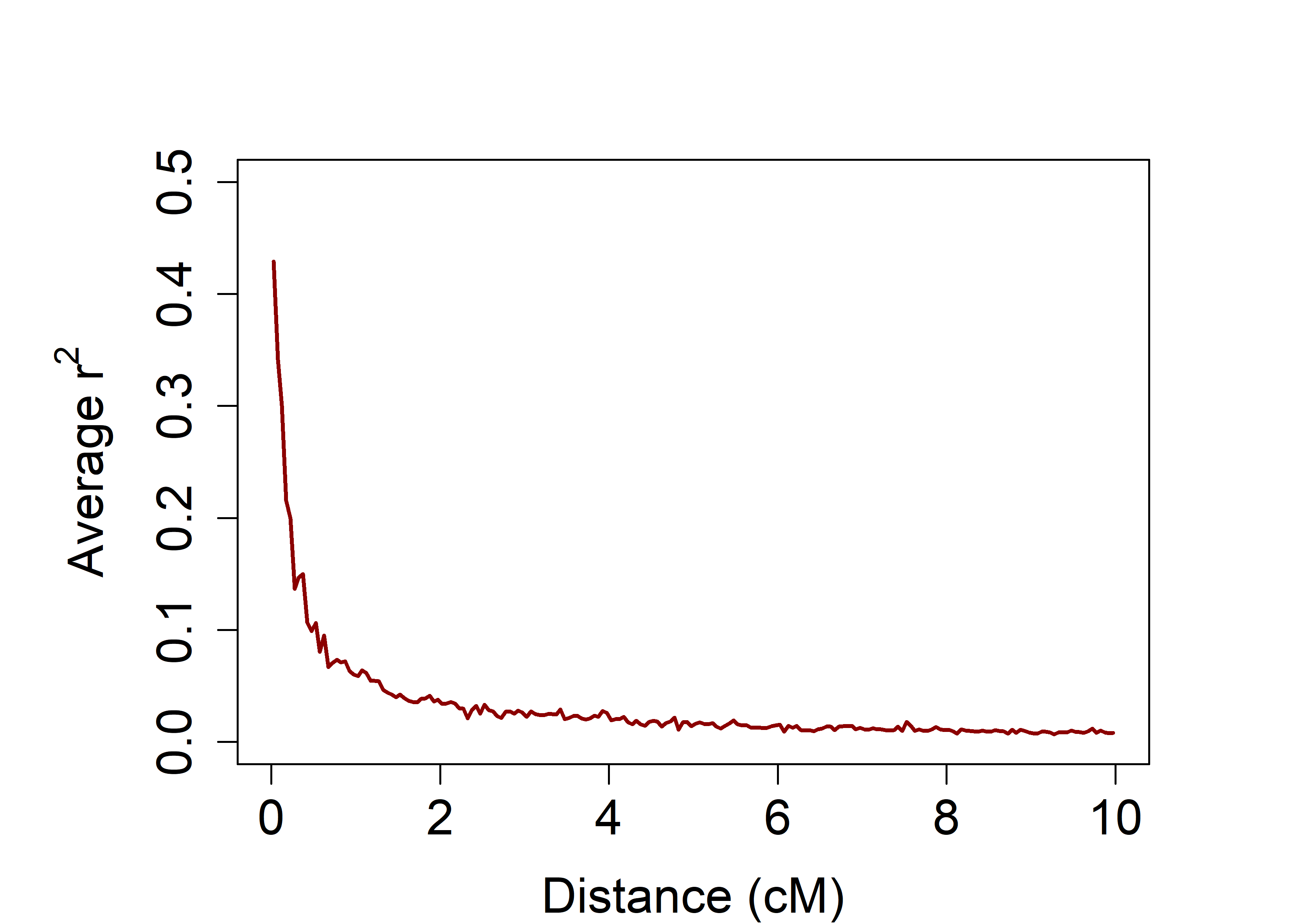


**FIGURE S2**

Extent of LD (*r^2^*) in the simulated population before selection as a function of distance in one random replicate.


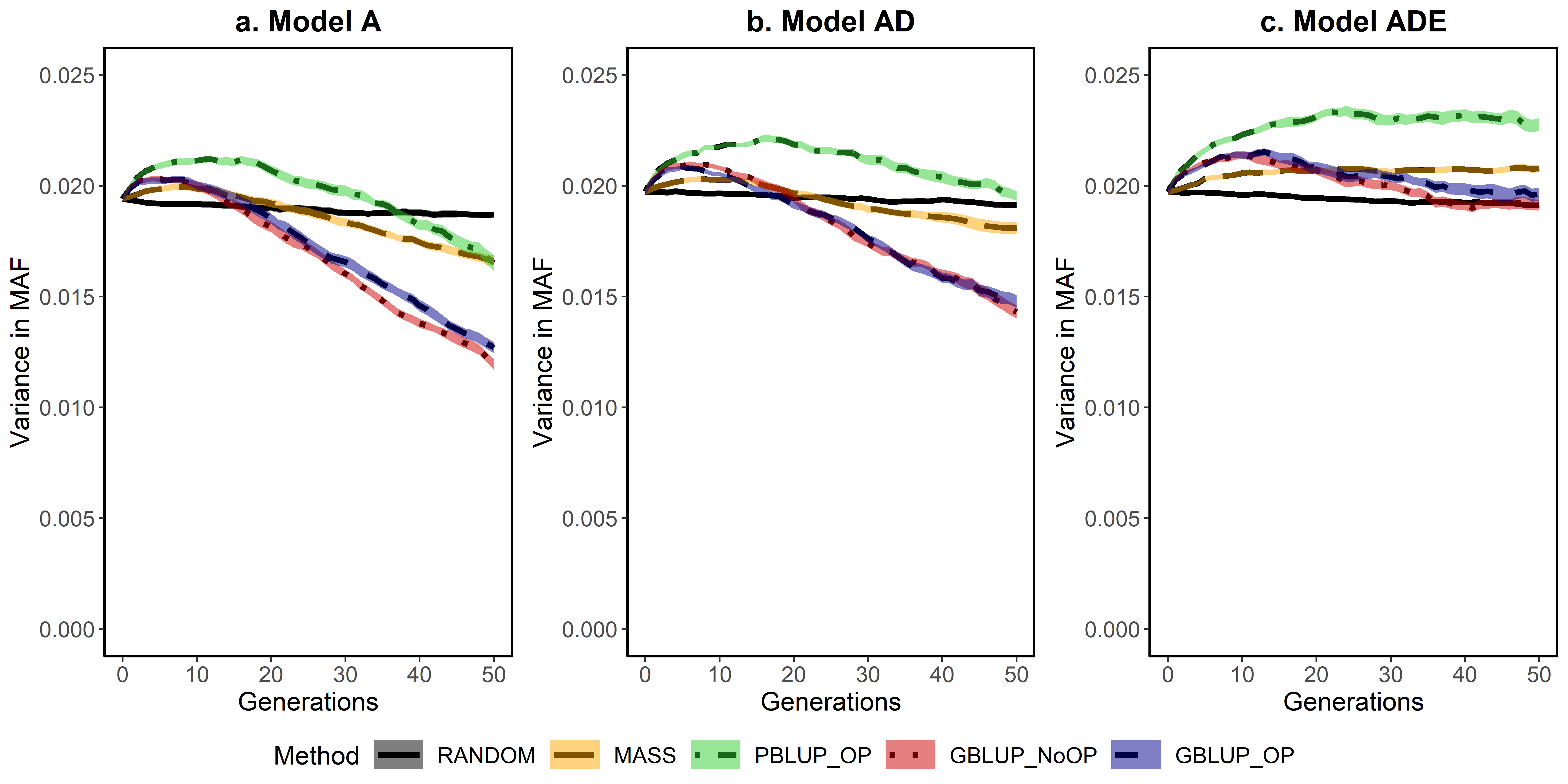


**FIGURE S3**

Trend in the variation in minor allele frequency (MAF) of segregating causal loci for the five selection methods and three genetic models. The five selection methods were: RANDOM selection, MASS selection, PBLUP selection with own performance (PBLUP_OP), GBLUP selection without own performance (GBLUP_NoOP) or with own performance (GBLUP_OP). The three genetic models were a model with only additive effects (A), with additive and dominance effects (AD), or with additive, dominance and epistatic effects (ADE). Results are shown as averages of 20 replicates and the width of the lines represents the average plus and minus one standard error.


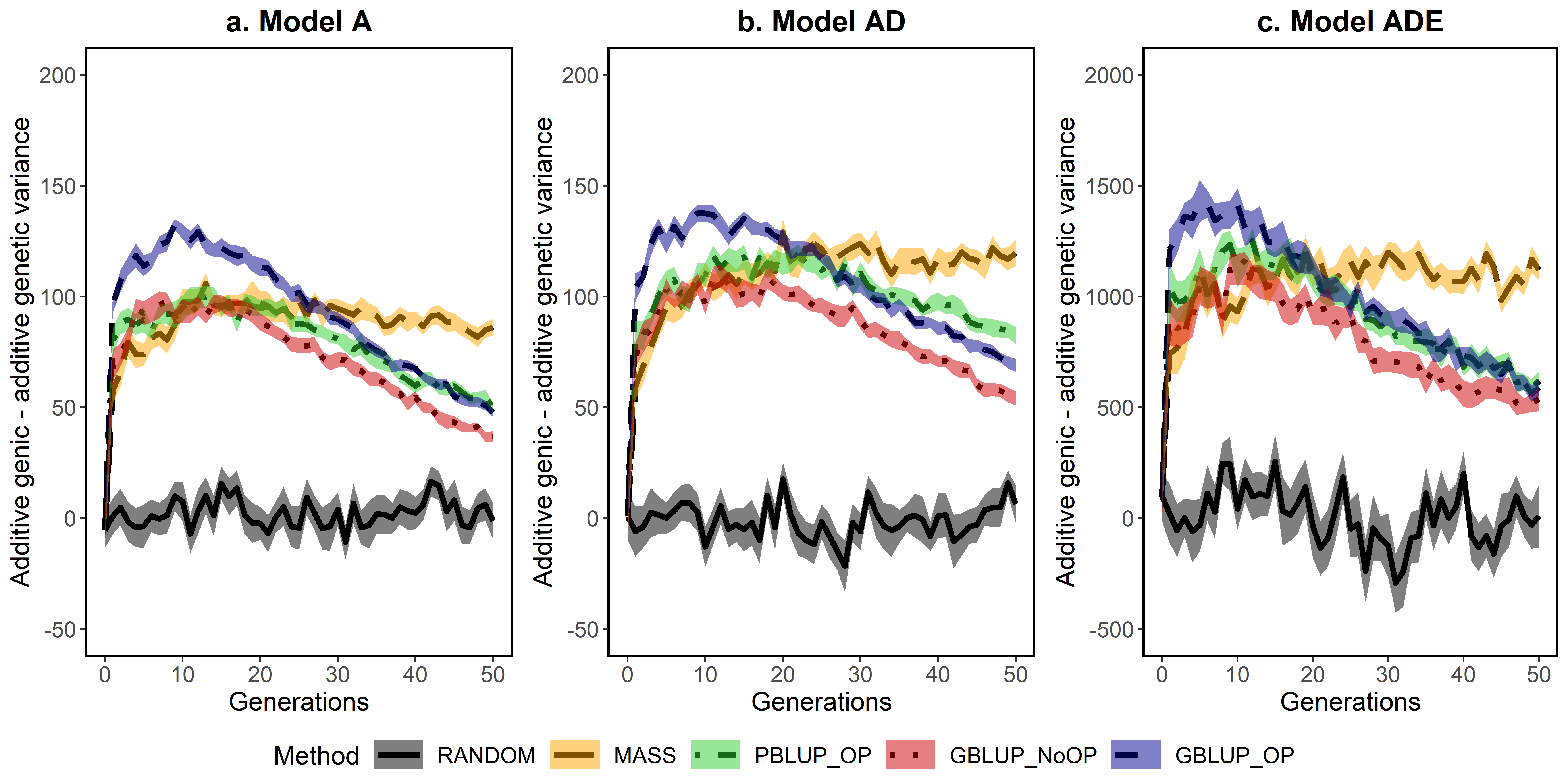


**FIGURE S4**

The difference between additive genic and additive genetic variance which represents a transient loss in genetic variance for the five selection methods and three genetic models. The five selection methods were: RANDOM selection, MASS selection, PBLUP selection with own performance (PBLUP_OP), GBLUP selection without own performance (GBLUP_NoOP) or with own performance (GBLUP_OP). The three genetic models were a model with only additive effects (A), with additive and dominance effects (AD), or with additive, dominance and epistatic effects (ADE). Results are shown as averages of 20 replicates and the width of the lines represents the average plus and minus one standard error.


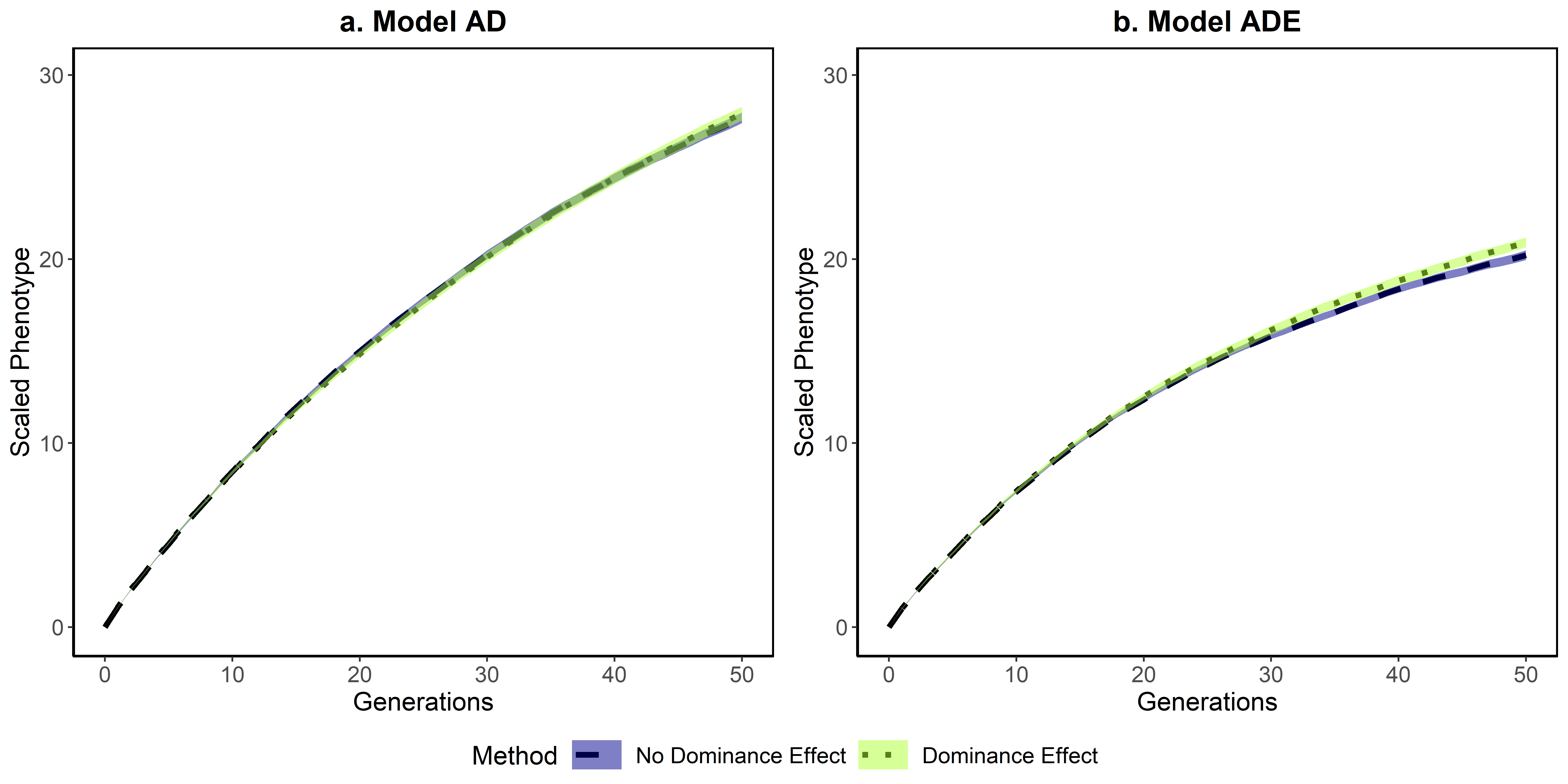


**FIGURE S5**

Phenotypic trend for the GBLUP model with own performance records and with and without a dominance effect for the genetic models with non-additive effects. The phenotypic trend is scaled by the additive genetic standard deviation in the generation before selection in order to make the results comparable across the genetic models. The two genetic models were a model with additive and dominance effects (AD), or with additive, dominance and epistatic effects (ADE). Results are shown as averages of 20 replicates and the width of the lines represents the average plus and minus one standard error.


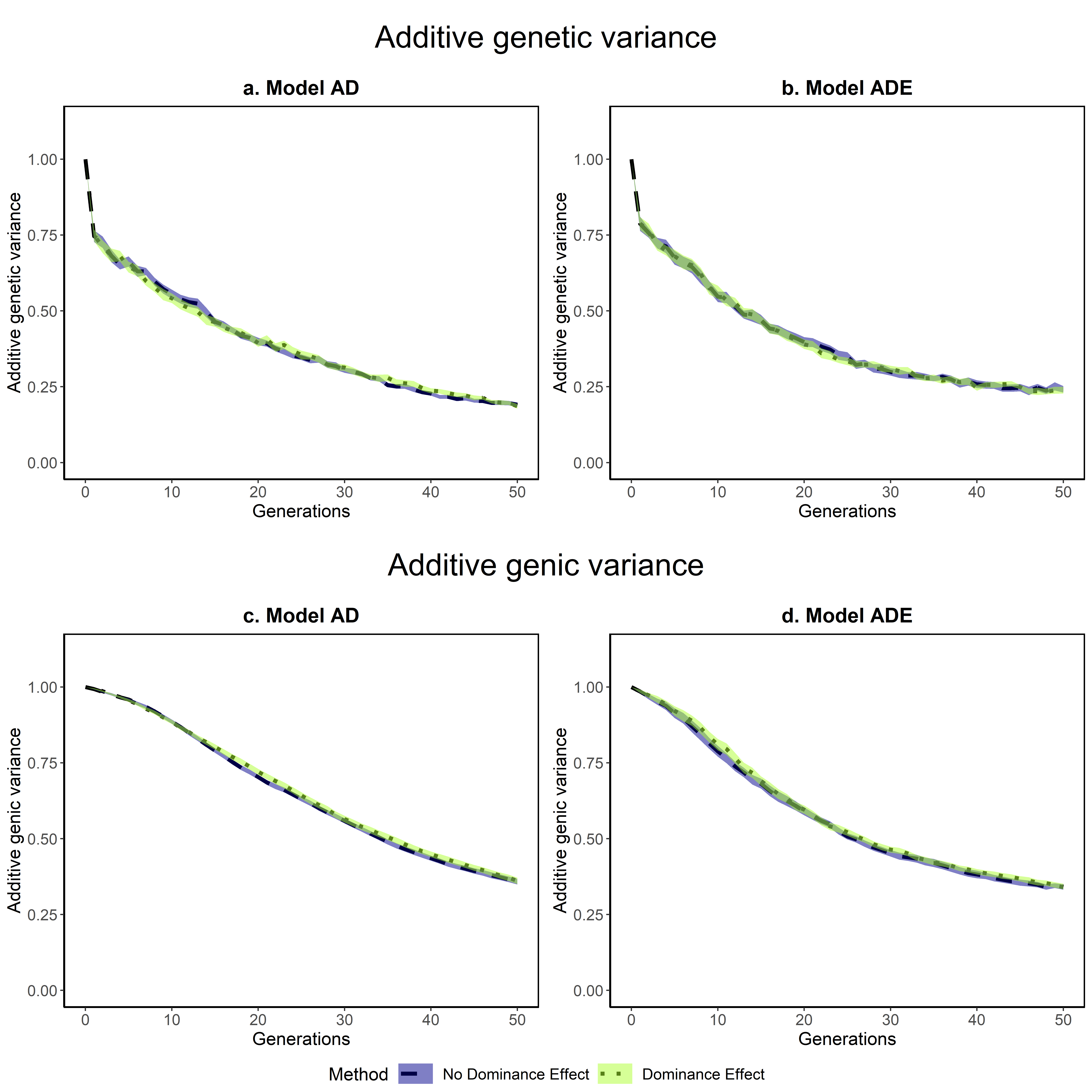


**FIGURE S6**

Trend in additive genetic (a, b) and additive genic (c, d) variance for the GBLUP model with own performance and with and without a dominance effect for the genetic models with non-additive effects. The trend is scaled by the additive genetic or additive genic variance in the generation before selection in order to make the results comparable across the genetic models. The two genetic models were a model with additive and dominance effects (AD), or with additive, dominance and epistatic effects (ADE). Results are shown as averages of 20 replicates and the width of the lines represents the average plus and minus one standard error.
