# Supplementary material for: The long-term effects of genomic selection: 1. Response to selection, additive genetic variance, and genetic architecture
Source: Genet Sel Evol. 2022 Mar 7;54:19. doi: 10.1186/s12711-022-00709-7 (PMC8900405; doi:10.1186/s12711-022-00709-7)
Supplement: Supplementary file 3 — Additional file 3. Decomposition of additive genetic variance. This file provides a theoretical decomposition of the additive genetic variance. [file 12711_2022_709_MOESM3_ESM.docx]

**Additional file 3: Decomposition of additive genetic variance**

The total additive genetic variance ($\sigma_{A,genetic}^{2}$) in a population is the variance of the additive genetic values, also known as true breeding values, of all individuals in that population. The additive genetic value of an individual can be calculated based on its genotype and the additive genetic effects (α) in that population.

The additive genic variance in a population can be calculated as:

$\sigma_{A,genic}^{2}=\sum_{i=1}^{n} 2p_{i}(1-p_{i})\alpha_{i}^{2}$,

where *n* is the number of causal loci, *p*_i_ the allele frequency of causal locus *i* and $\alpha_{i}$ the additive genetic effect of causal locus *i*.

The additive genic variance is equal to the additive genetic variance when the covariance between the additive genetic value of two causal loci across individuals is zero. This doesn’t require that all causal loci are in linkage equilibrium, but that the sign of linkage disequilibrium is not related to the sign of the statistical additive effects. Generally, this is the case in a population in the absence of selection, when the Bulmer effect is absent, because selection creates a negative covariance between causal loci under selection [72].

The loss in additive genetic variance due to selection as a result of a negative covariance between loci can be calculated as:

$\sigma_{A,loss}^{2}=\sigma_{A,genetic}^{2}- \sigma_{A,genic}^{2}=\sigma_{A,genetic}^{2}- \sum_{i=1}^{n} 2p_{i}\left( 1-p_{i} \right)\alpha_{i}^{2}$.

The additive genic variance is depending on the number of segregating causal loci, the average heterozygosity ($\bar{H_{E}}$) at those loci, the average $\alpha^{2}$ and the covariance between the heterozygosity and $\alpha^{2}$ across causal loci, which follows from:

$$\sigma_{A,genic}^{2}=\sum_{i=1}^{n} 2p_{i}\left( 1-p_{i} \right)\alpha_{i}^{2}=n\bar{2p\left( 1-p \right)\alpha^{2}}=nE\left[ 2p(1-p{)\alpha}^{2} \right]=nE\left[ 2p(1-p) \right]E\left[ \alpha^{2} \right]+cov\left[ 2p\left( 1-p \right),\alpha^{2} \right]=n\left\{ \bar{H_{E}}\bar{\alpha^{2}}+cov\left( H_{E},\alpha^{2} \right) \right\}$$

The average heterozygosity is depending on the average minor allele frequency (MAF) of the causal loci (*p_MAF_*) and the variation in MAF across causal loci, which follows from:

$$\bar{H_{E}}=E\left[ 2p(1-p) \right]=E\left[ 2p_{MAF}(1-p_{MAF}) \right]=2E\left[ p_{MAF}-p_{MAF}^{2} \right]=2\left\{ E\left[ p_{MAF} \right]-E\left[ p_{MAF}^{2} \right] \right\}=2\left\{ \bar{p_{MAF}}-\left[ \bar{p_{MAF}^{2}}+var(p_{MAF}) \right] \right\}=2\bar{p_{MAF}}\left( 1-\bar{p_{MAF}} \right)-2var(\bar{p_{MAF}})$$

The accumulated heterozygosity follows from the average heterozygosity multiplied by the number of segregating causal loci:

$$H_{E,Accumulated}=n\bar{H_{E}}$$
