# Supplementary material for: The long-term effects of genomic selection: 1. Response to selection, additive genetic variance, and genetic architecture
Source: Genet Sel Evol. 2022 Mar 7;54:19. doi: 10.1186/s12711-022-00709-7 (PMC8900405; doi:10.1186/s12711-022-00709-7)
Supplement: Supplementary file 4 — Additional file 4: Figure S1. Scatterplot of allele frequencies of all causal variants in different generations for the genetic model with additive effects (Model A) under RANDOM selection. Figure S2. Scatterplot of allele frequencies of all causal variants in different generations for the genetic model with additive effects (Model A) under MASS selection. Figure S3. Scatterplot of allele frequencies of all causal variants in different generations for the genetic model with additive effects (Model A) under PBLUP selection with own performance (PBLUP_OP). Figure S4. Scatterplot of allele frequencies of all causal variants in different generations for the genetic model with additive effects (Model A) under GBLUP selection without own performance (GBLUP_NoOP). Figure S5. Scatterplot of allele frequencies of all causal variants in different generations for the genetic model with additive effects (Model A) under GBLUP selection with own performance (GBLUP_OP). Figure S6. Scatterplot of allele frequencies of all causal variants in different generations for the genetic model with additive and dominance effects (Model AD) under RANDOM selection. Figure S7. Scatterplot of allele frequencies of all causal variants in different generations for the genetic model with additive and dominance effects (Model AD) under MASS selection. Figure S8. Scatterplot of allele frequencies of all causal variants in different generations for the genetic model with additive and dominance effects (Model AD) under PBLUP selection with own performance (PBLUP_OP). Figure S9. Scatterplot of allele frequencies of all causal variants in different generations for the genetic model with additive and dominance effects (Model AD) under GBLUP selection without own performance (GBLUP_NoOP). Figure S10. Scatterplot of allele frequencies of all causal variants in different generations for the genetic model with additive and dominance effects (Model AD) under GBLUP selection with own performance (GBLUP_OP). Fig [file 12711_2022_709_MOESM4_ESM.docx]

**Additional file 4: Scatterplot of allele frequencies of all causal variants in different generations**


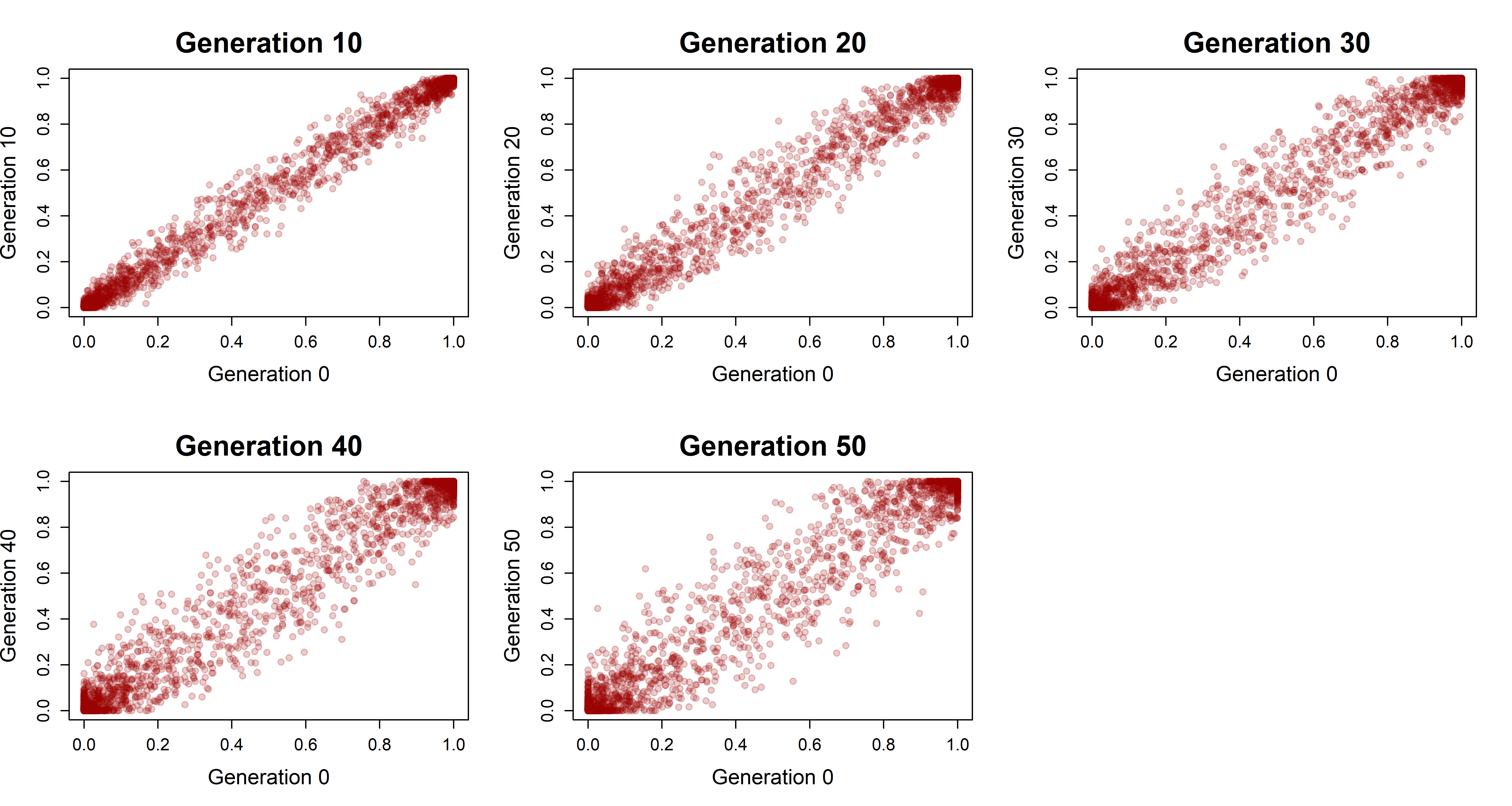


**FIGURE S1**

Scatterplot of allele frequencies of all causal variants in different generations for the genetic model with additive effects (Model A) under RANDOM selection.


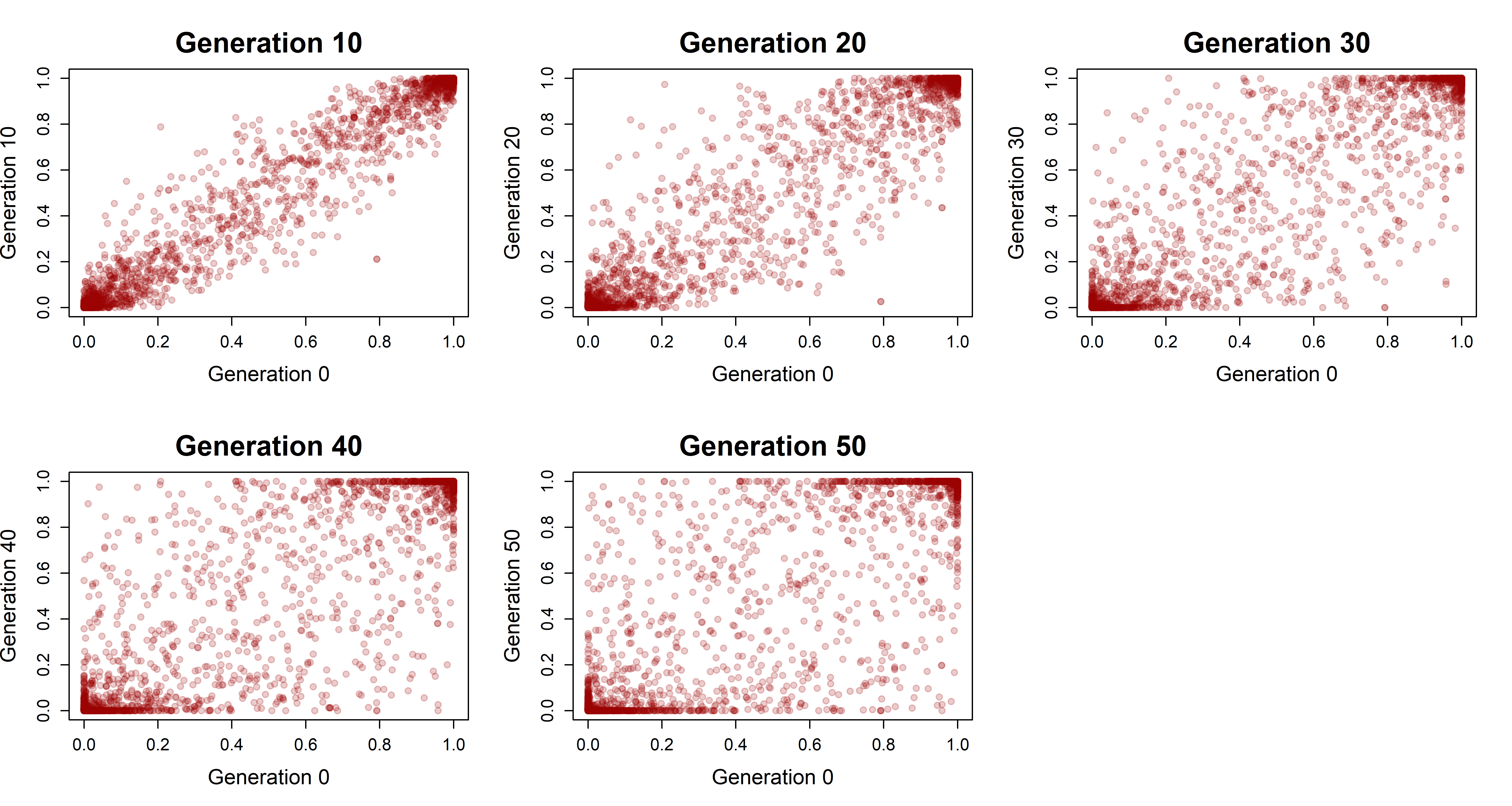


**FIGURE S2**

Scatterplot of allele frequencies of all causal variants in different generations for the genetic model with additive effects (Model A) under MASS selection.


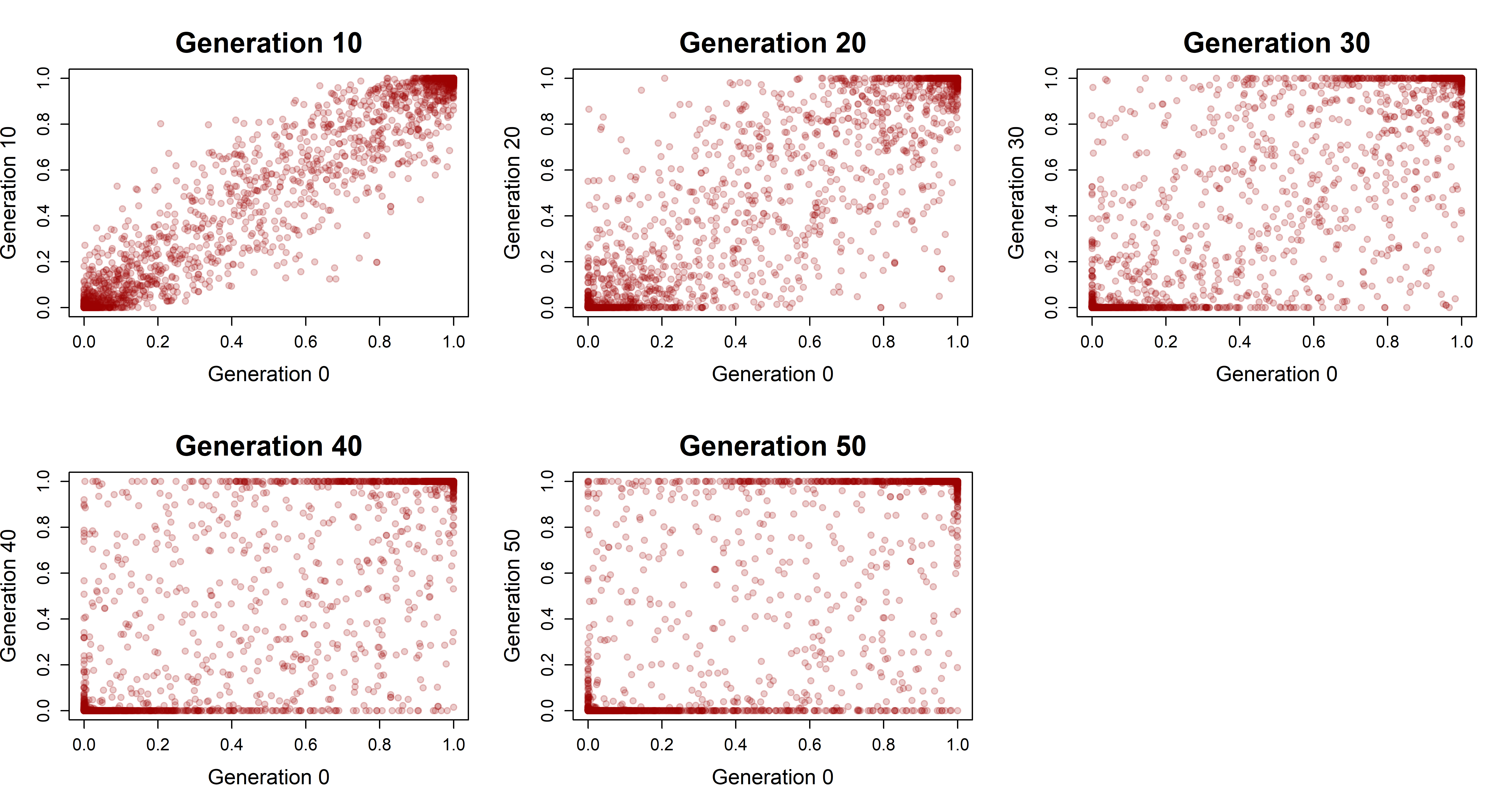


**FIGURE S3**

Scatterplot of allele frequencies of all causal variants in different generations for the genetic model with additive effects (Model A) under PBLUP selection with own performance (PBLUP_OP).


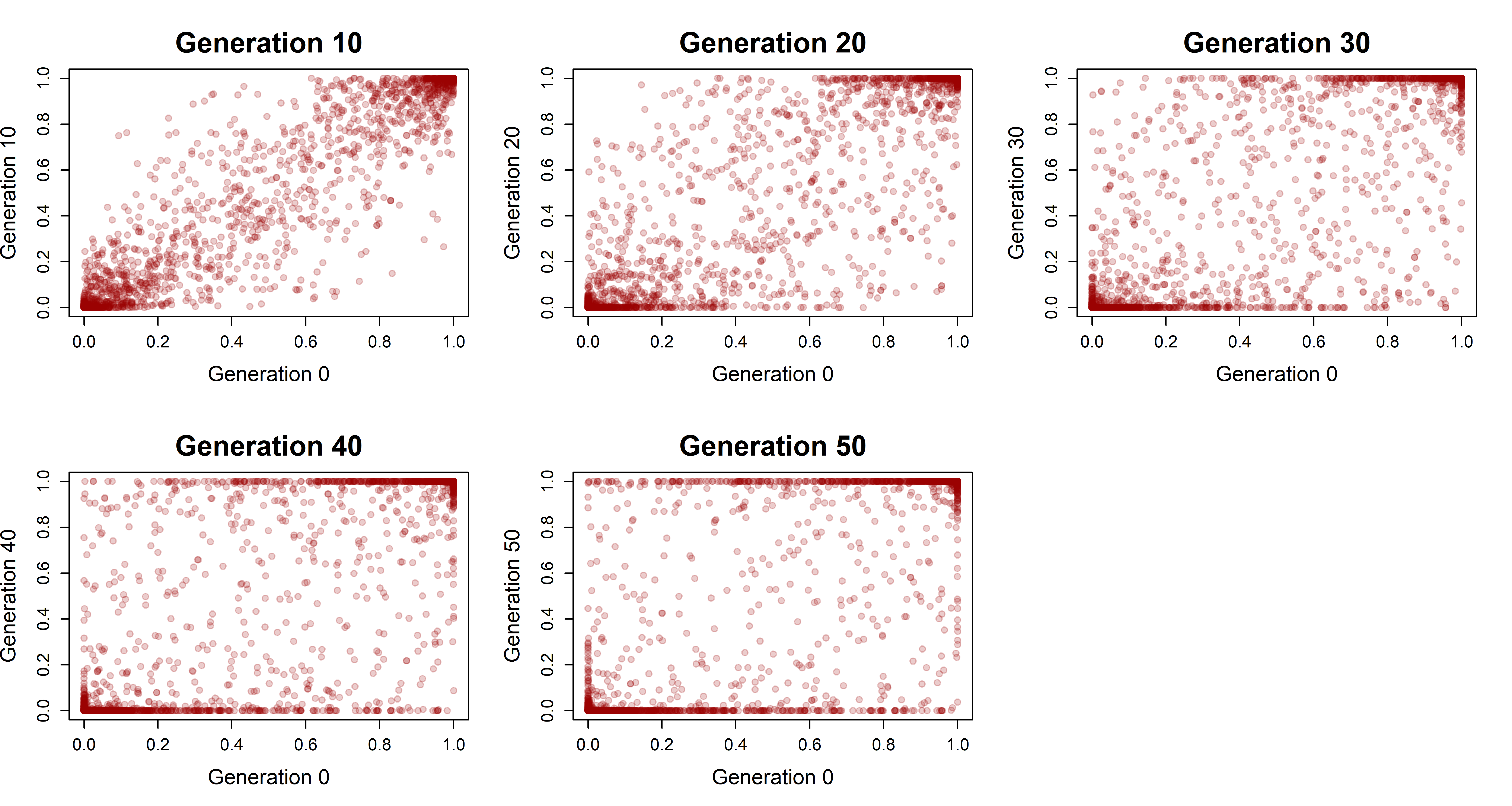


**FIGURE S4**

Scatterplot of allele frequencies of all causal variants in different generations for the genetic model with additive effects (Model A) under GBLUP selection without own performance (GBLUP_NoOP).


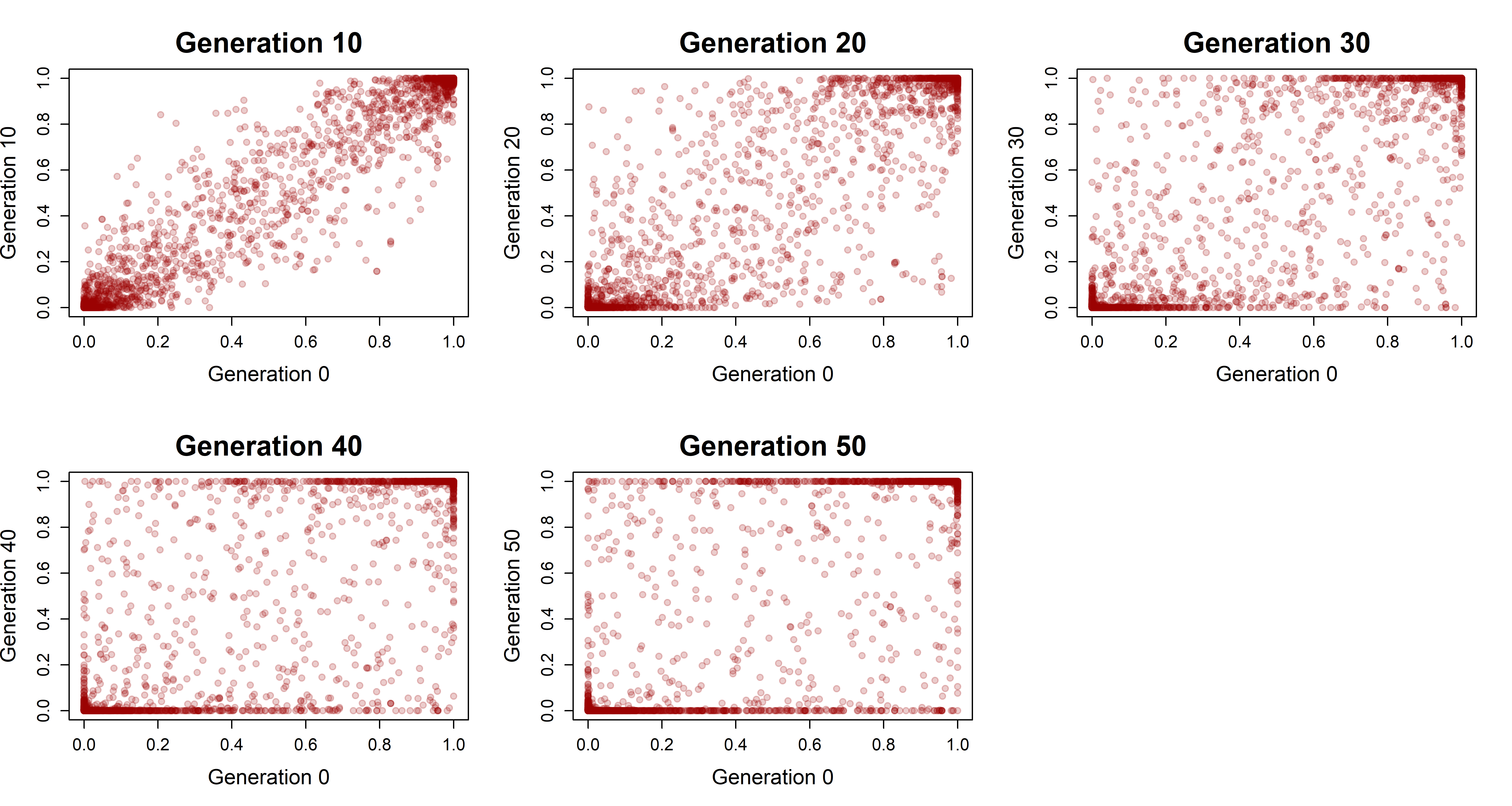


**FIGURE S5**

Scatterplot of allele frequencies of all causal variants in different generations for the genetic model with additive effects (Model A) under GBLUP selection with own performance (GBLUP_OP).


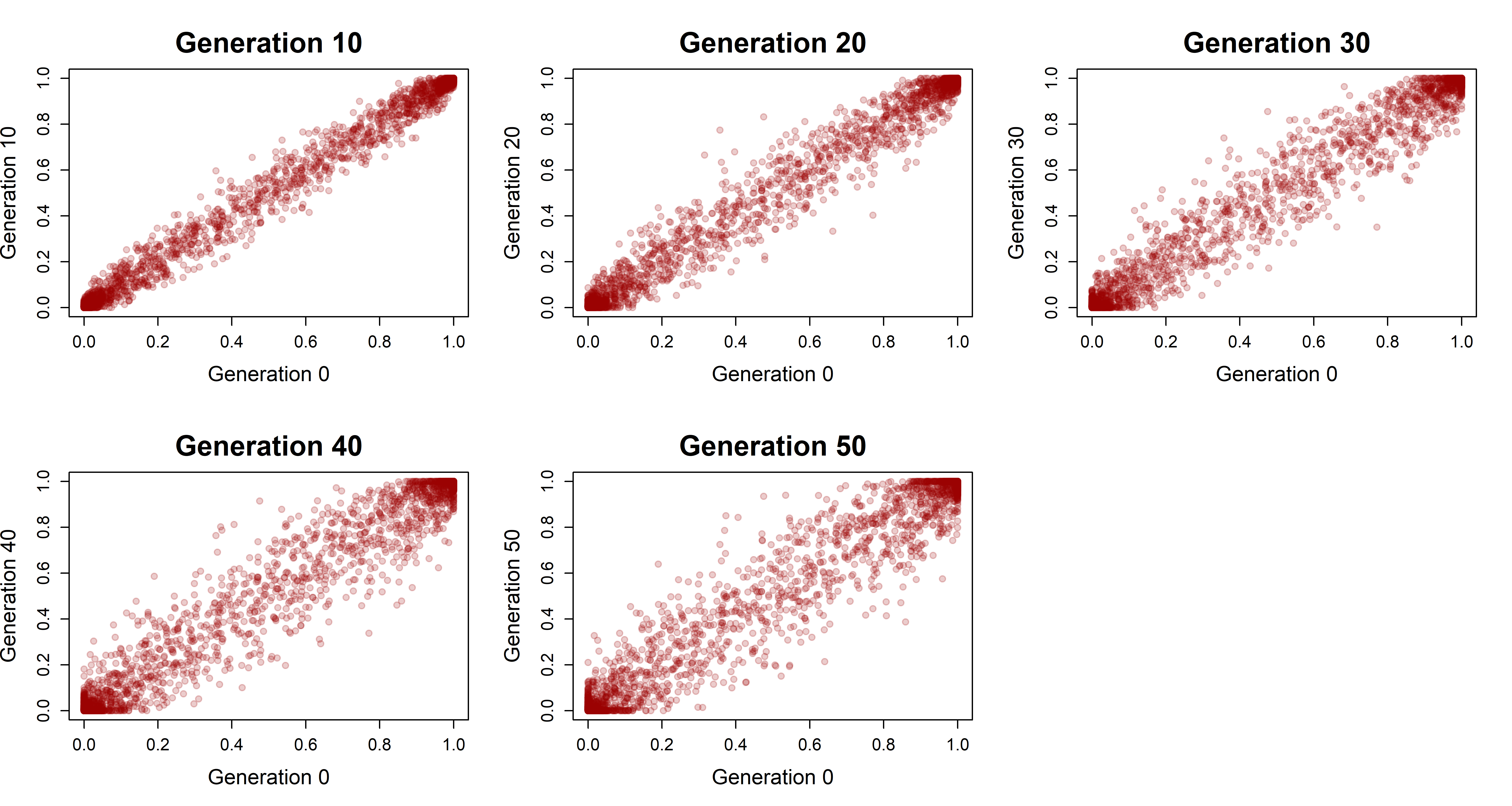


**FIGURE S6**

Scatterplot of allele frequencies of all causal variants in different generations for the genetic model with additive and dominance effects (Model AD) under RANDOM selection.


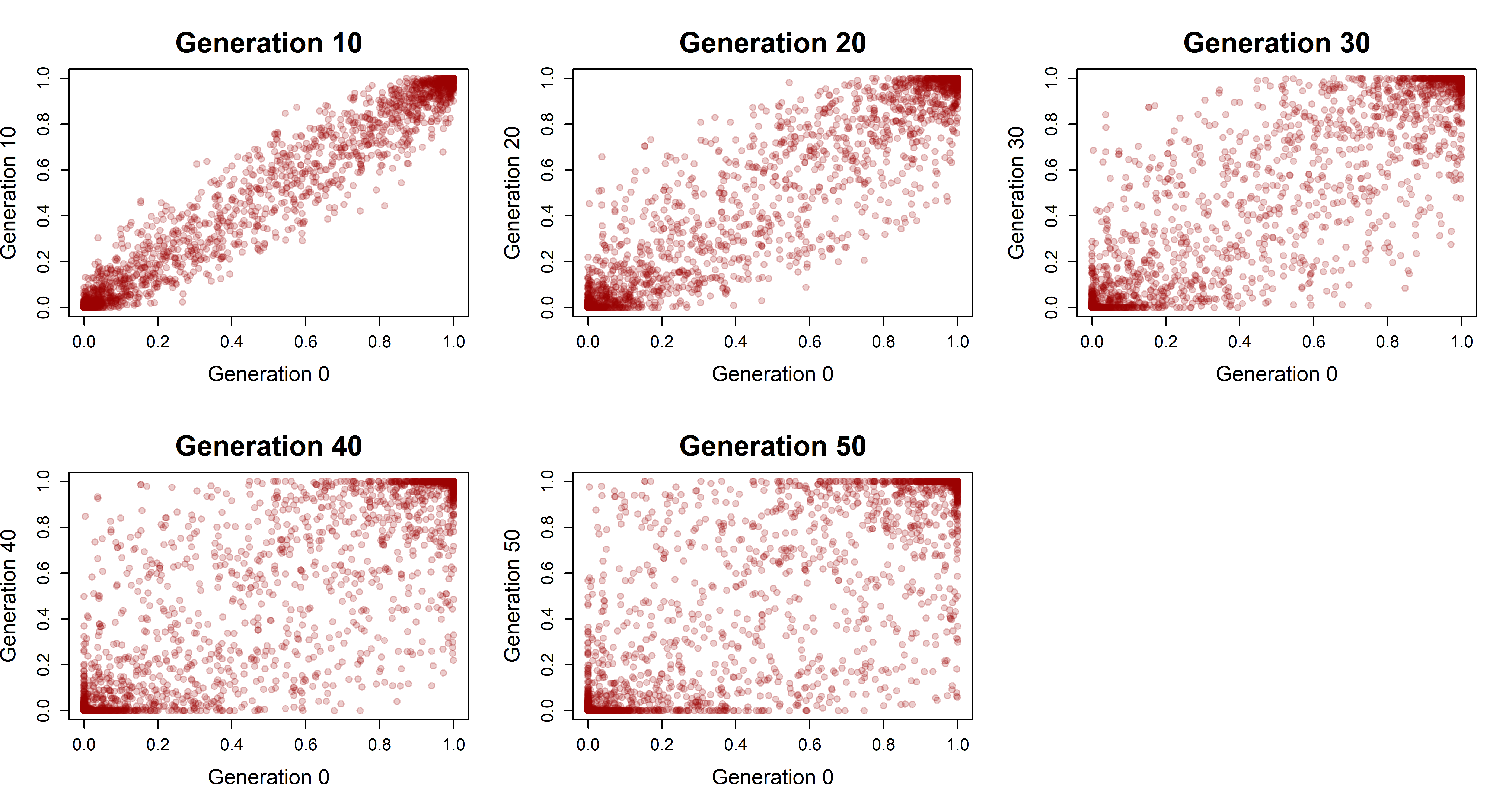


**FIGURE S7**

Scatterplot of allele frequencies of all causal variants in different generations for the genetic model with additive and dominance effects (Model AD) under MASS selection.


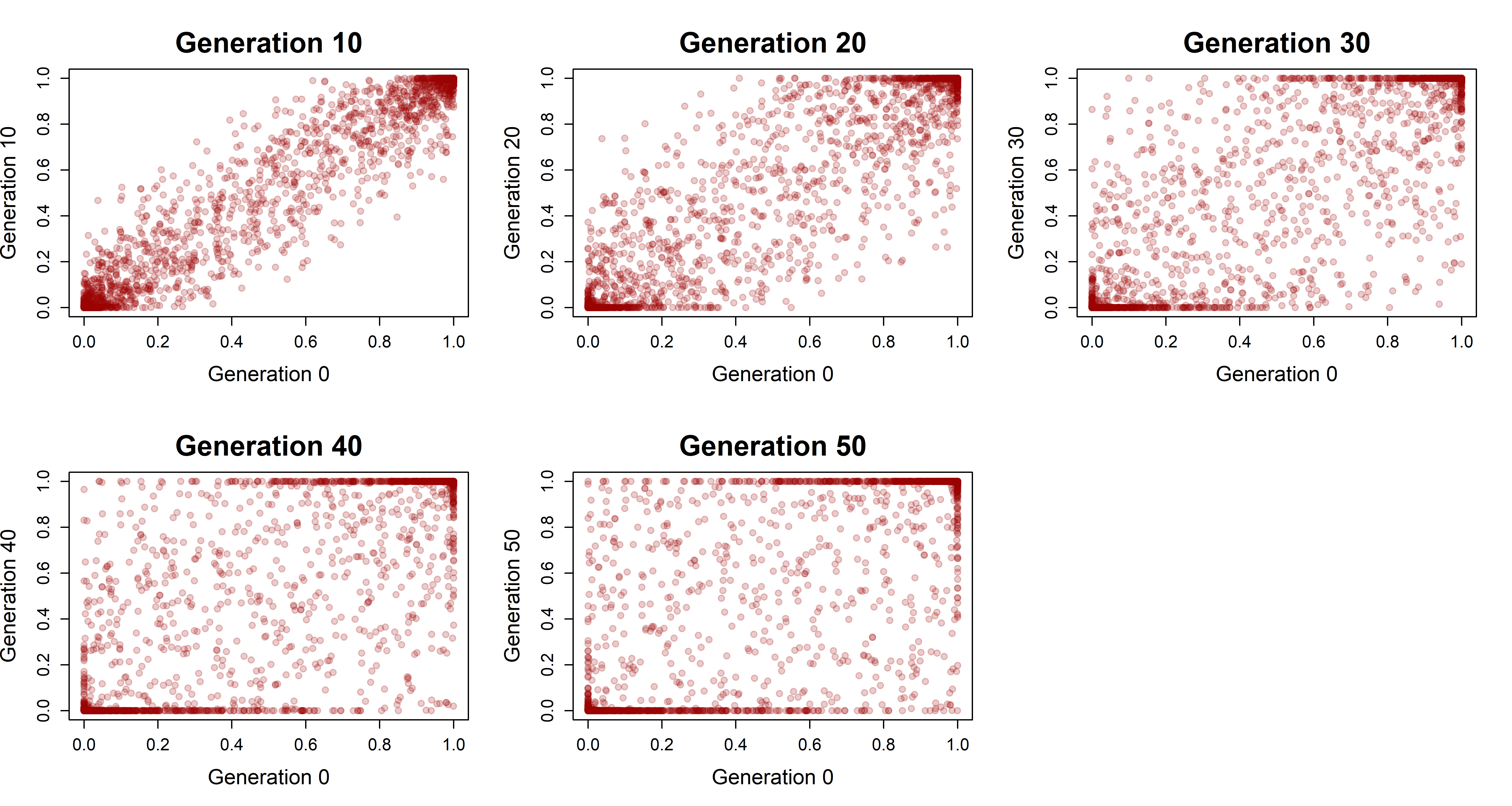


**FIGURE S8**

Scatterplot of allele frequencies of all causal variants in different generations for the genetic model with additive and dominance effects (Model AD) under PBLUP selection with own performance (PBLUP_OP).


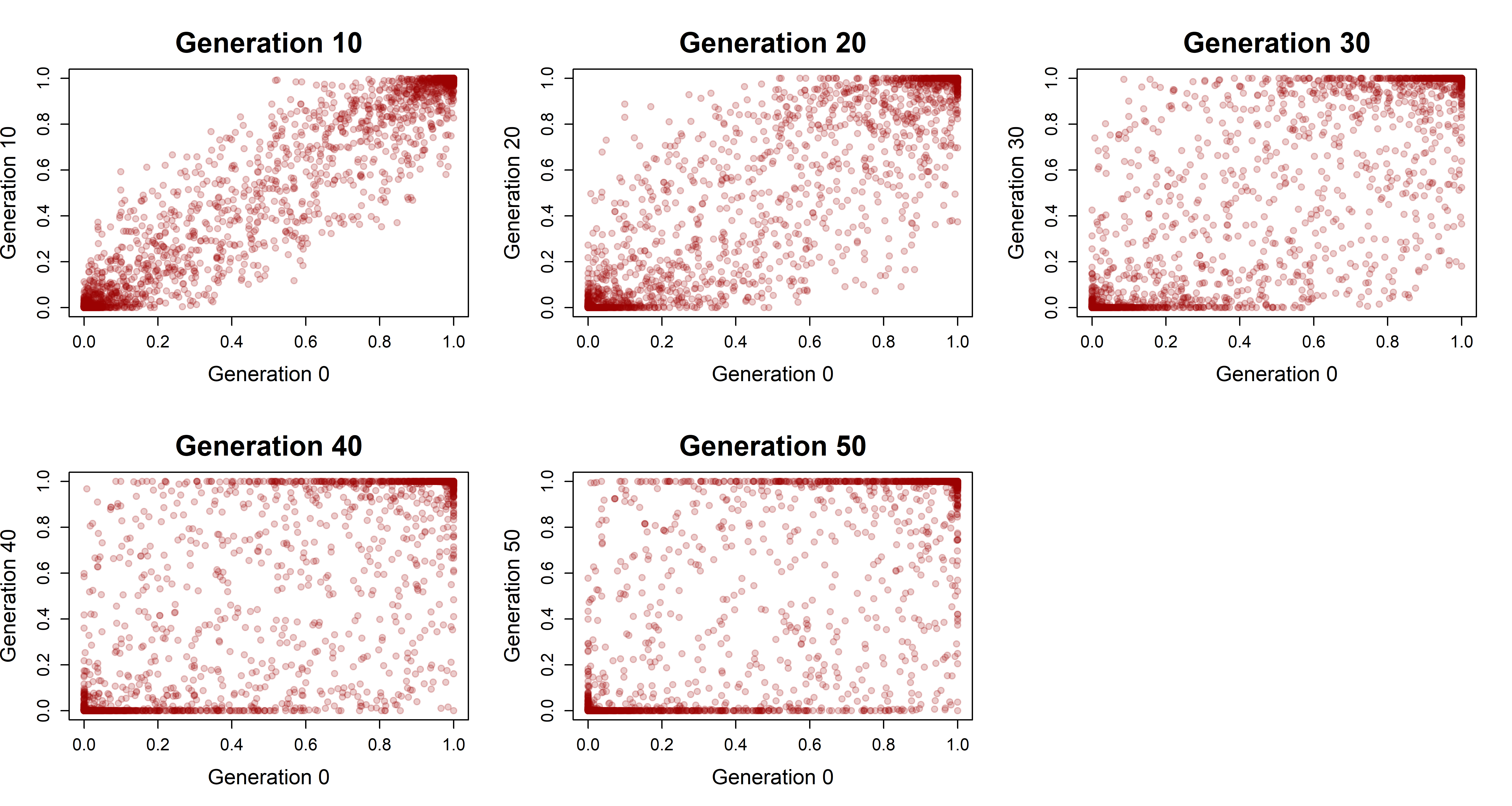


**FIGURE S9**

Scatterplot of allele frequencies of all causal variants in different generations for the genetic model with additive and dominance effects (Model AD) under GBLUP selection without own performance (GBLUP_NoOP).


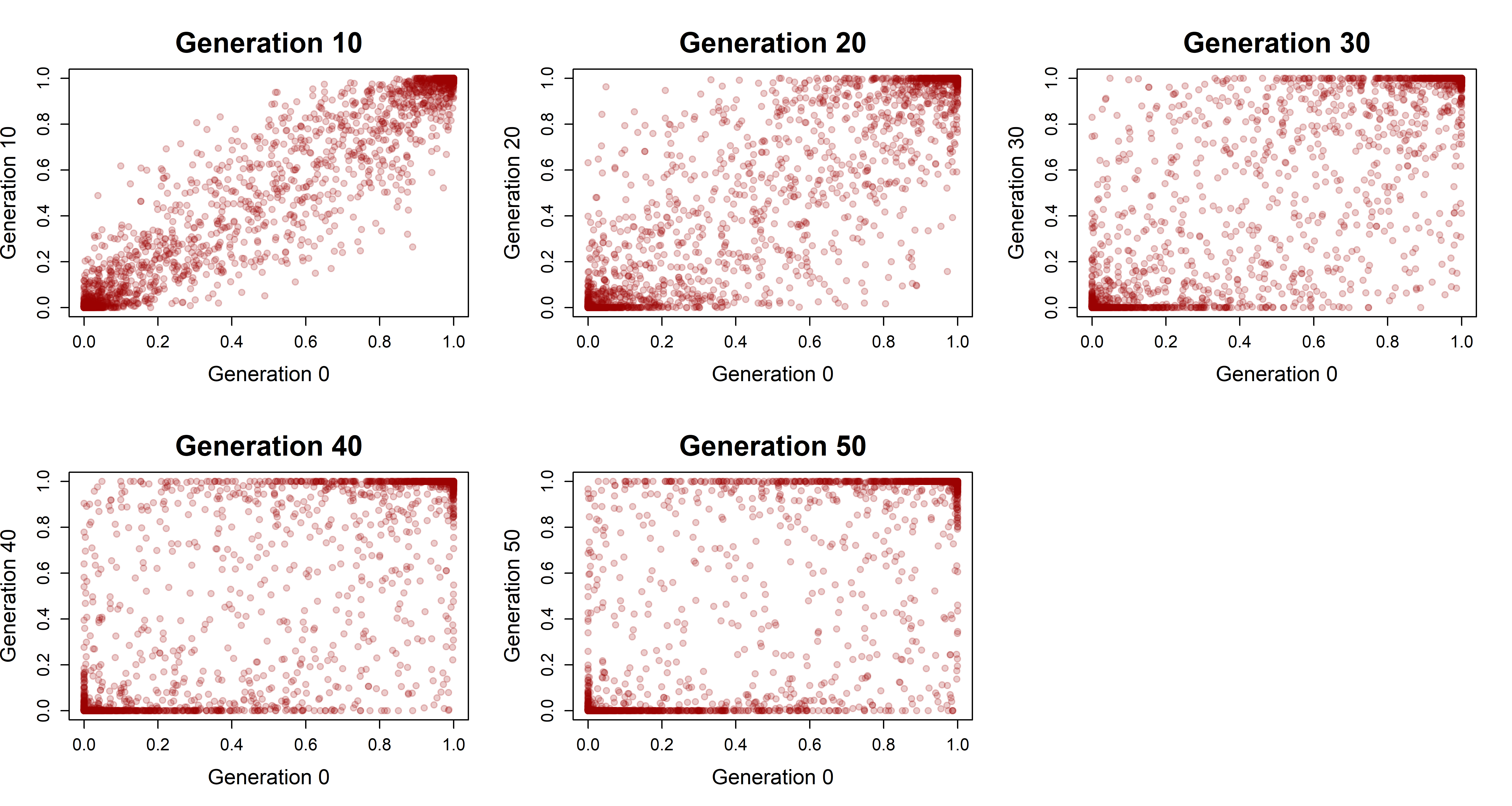


**FIGURE S10**

Scatterplot of allele frequencies of all causal variants in different generations for the genetic model with additive and dominance effects (Model AD) under GBLUP selection with own performance (GBLUP_OP).


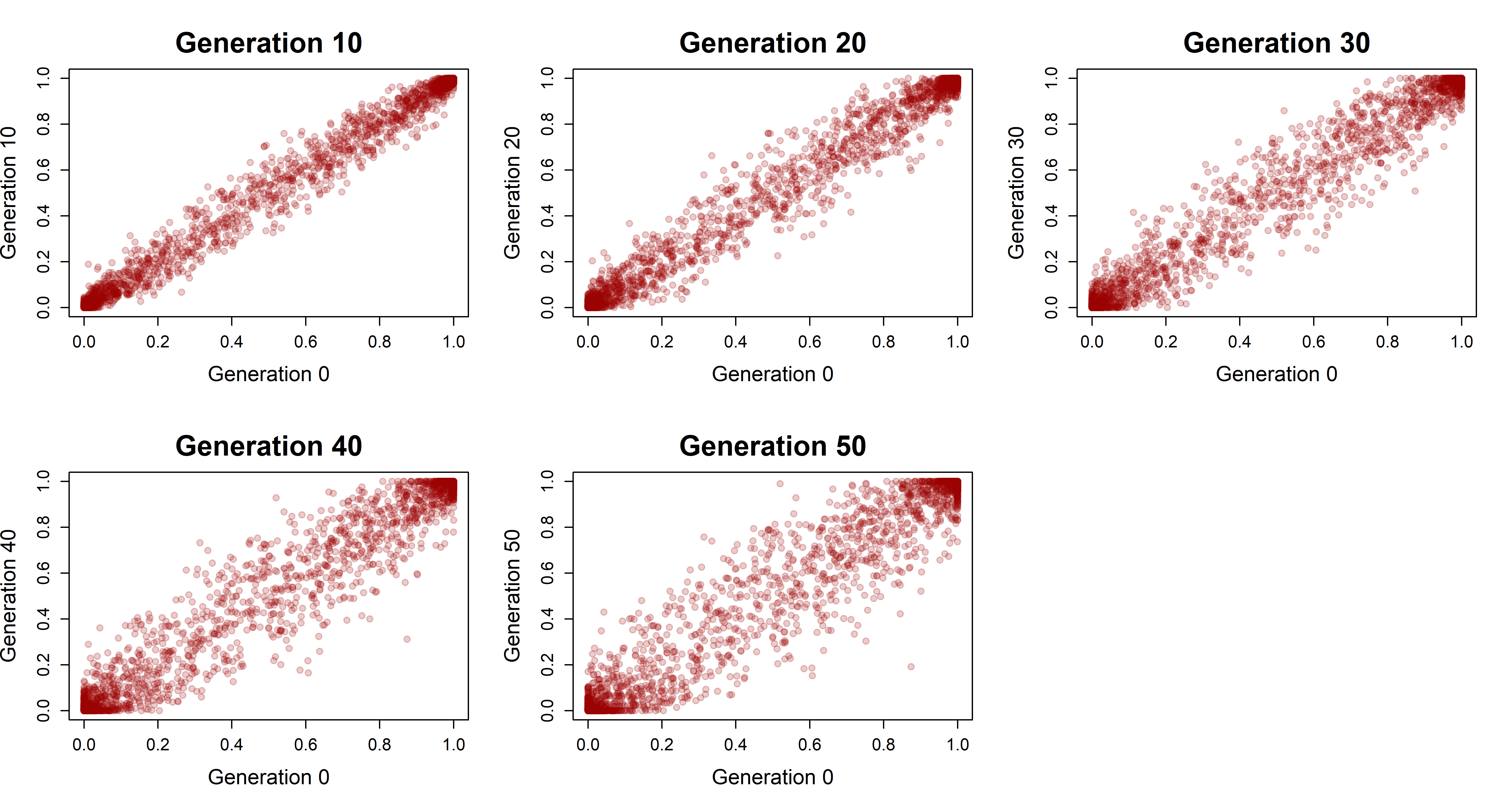


**FIGURE S11**

Scatterplot of allele frequencies of all causal variants in different generations for the genetic model with additive, dominance and epistatic effects (Model ADE) under RANDOM selection.


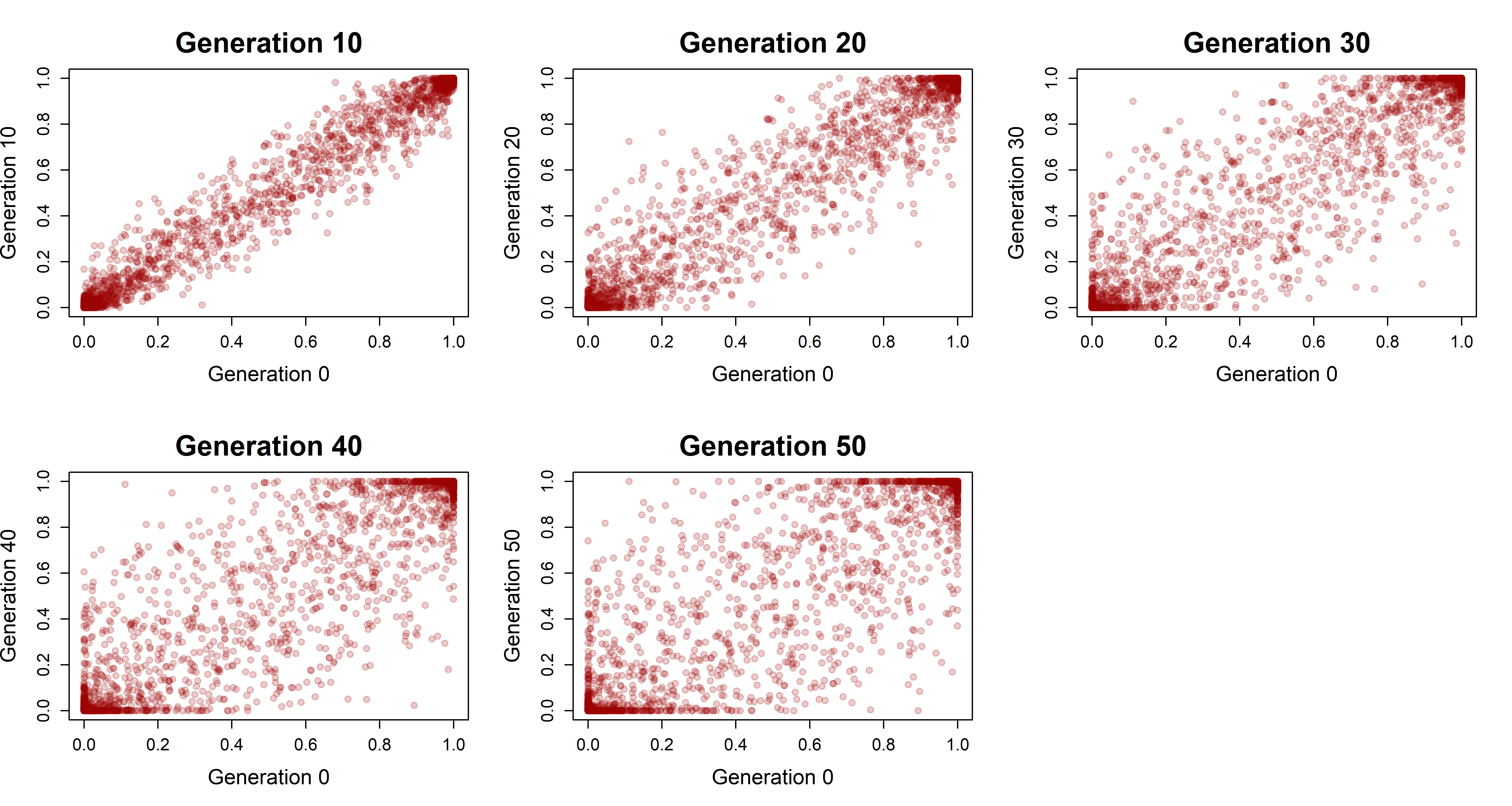


**FIGURE S12**

Scatterplot of allele frequencies of all causal variants in different generations for the genetic model with additive, dominance and epistatic effects (Model ADE) under MASS selection.


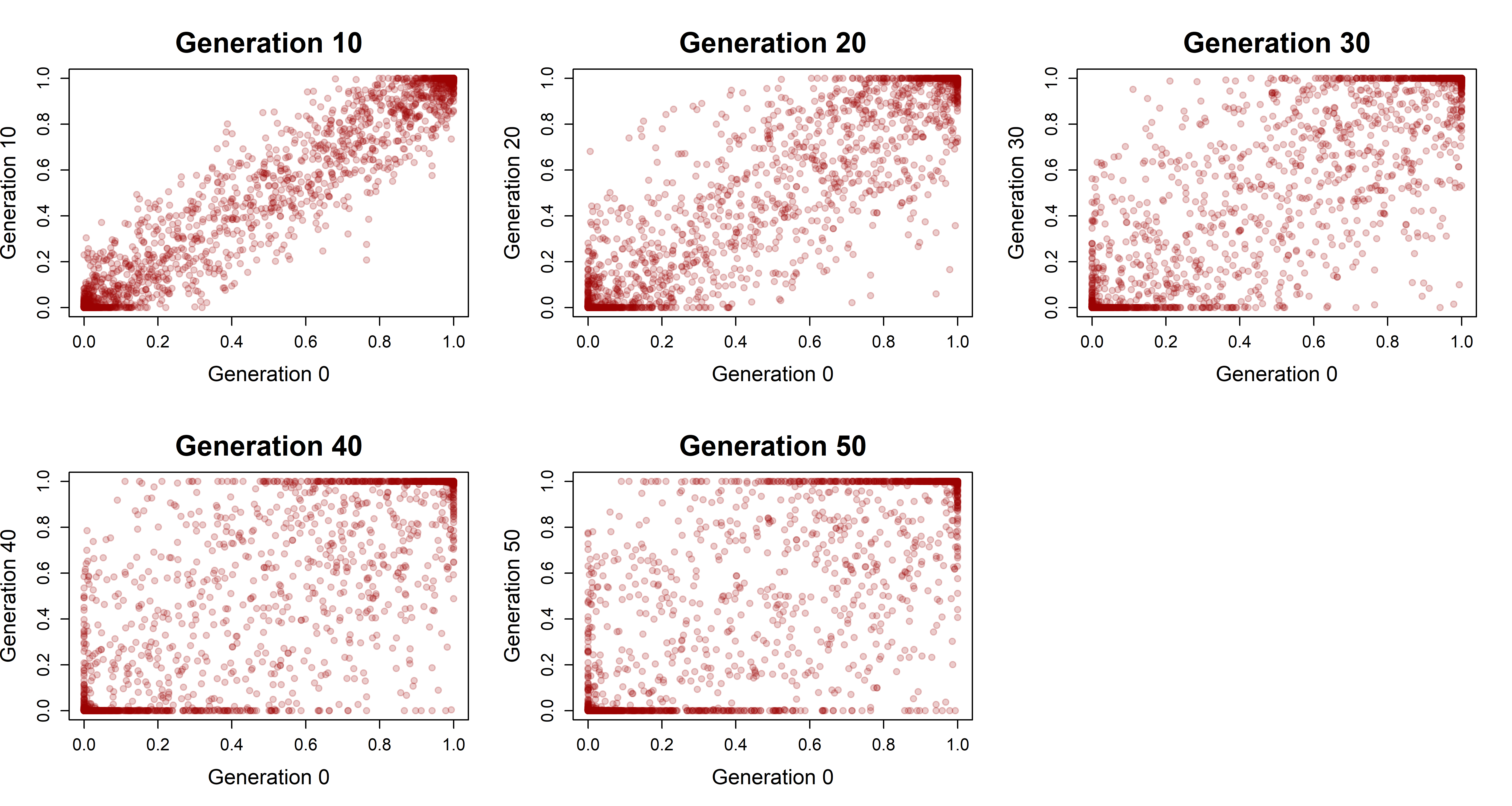


**FIGURE S13**

Scatterplot of allele frequencies of all causal variants in different generations for the genetic model with additive, dominance and epistatic effects (Model ADE) under PBLUP selection with own performance (PBLUP_OP).


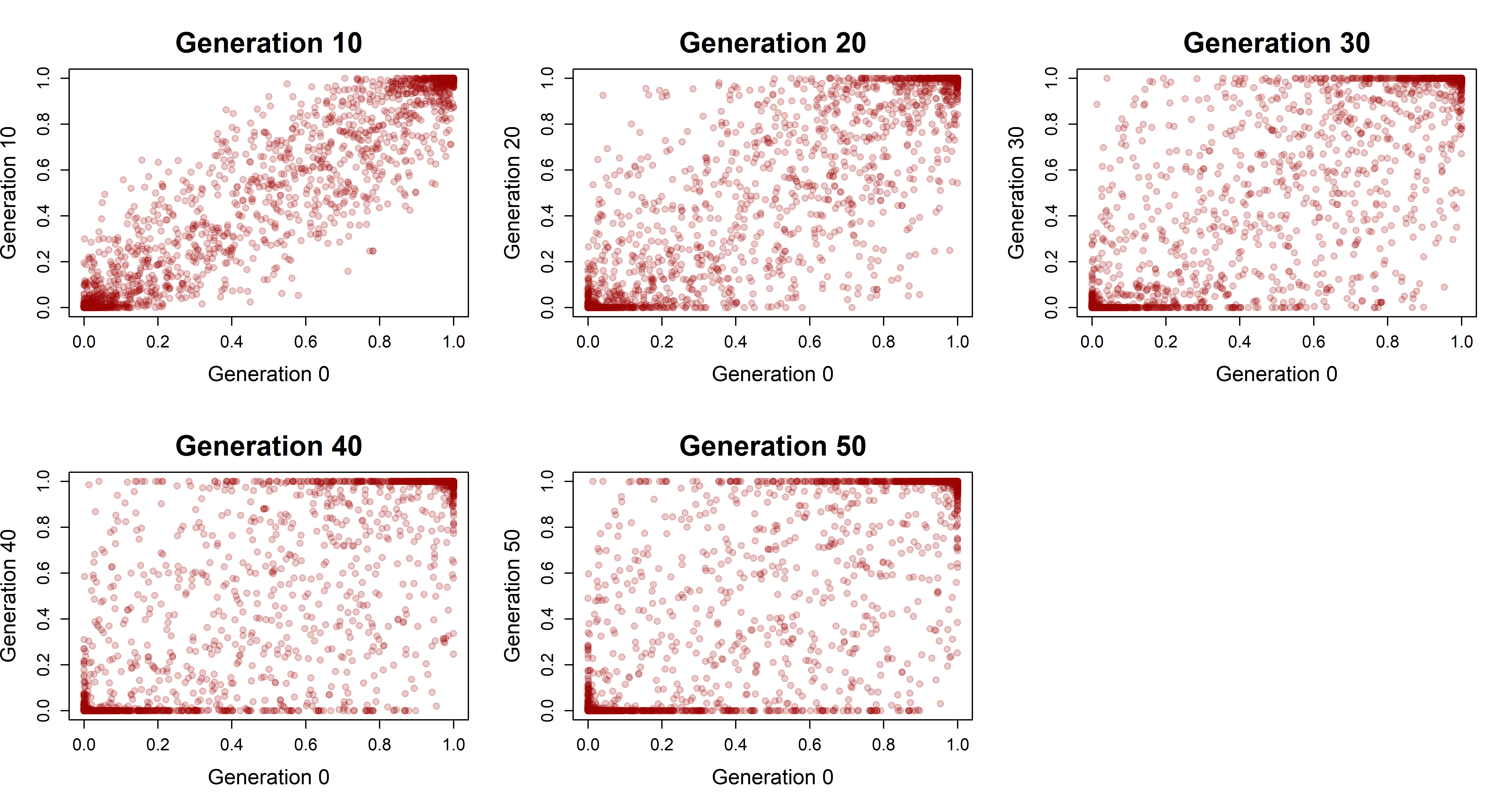


**FIGURE S14**

Scatterplot of allele frequencies of all causal variants in different generations for the genetic model with additive, dominance and epistatic effects (Model ADE) under GBLUP selection without own performance (GBLUP_NoOP).


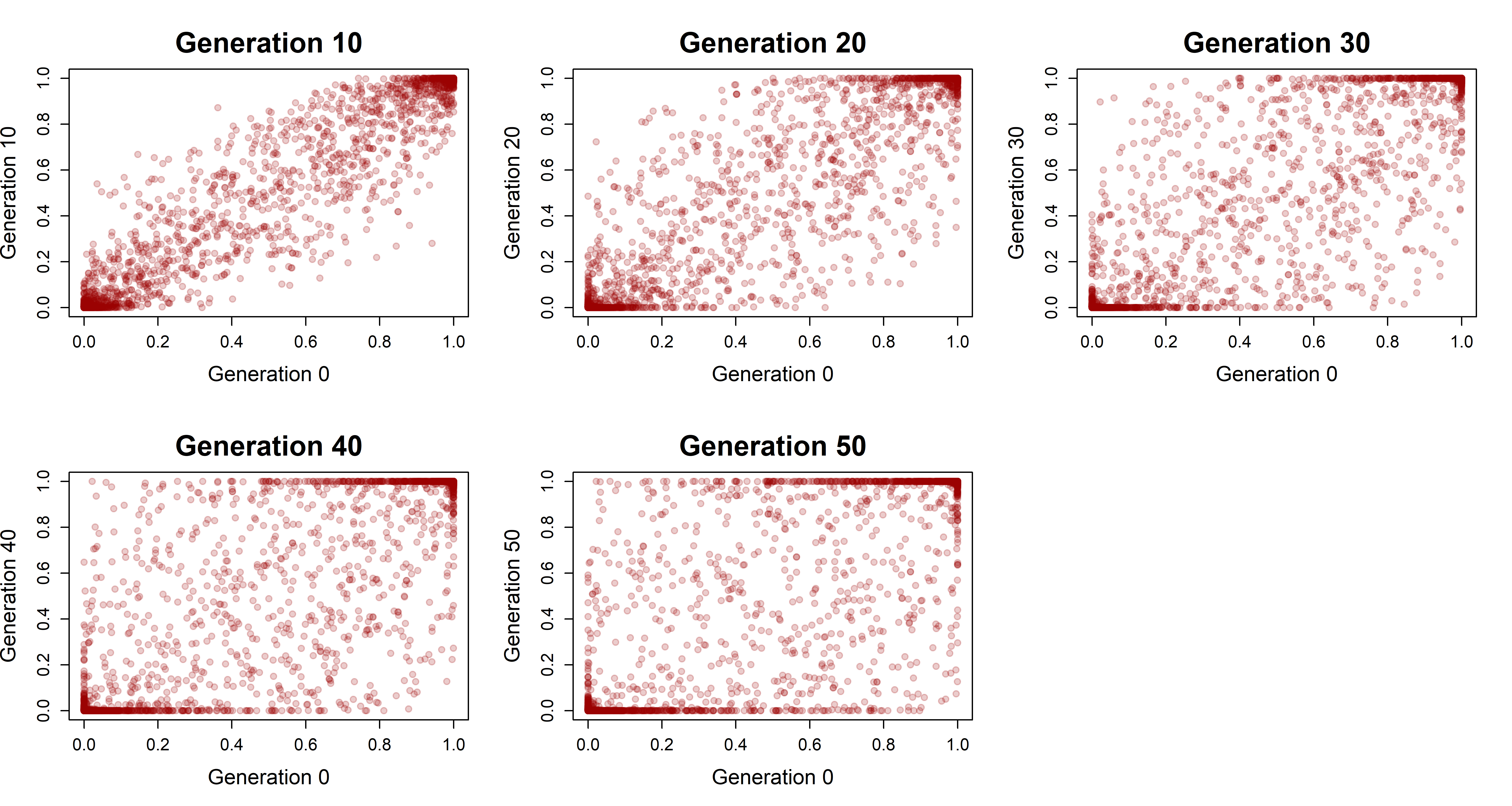


**FIGURE S15**

Scatterplot of allele frequencies of all causal variants in different generations for the genetic model with additive, dominance and epistatic effects (Model ADE) under GBLUP selection with own performance (GBLUP_OP).
