# Supplementary material for: The long-term effects of genomic selection: 1. Response to selection, additive genetic variance, and genetic architecture
Source: Genet Sel Evol. 2022 Mar 7;54:19. doi: 10.1186/s12711-022-00709-7 (PMC8900405; doi:10.1186/s12711-022-00709-7)
Supplement: Supplementary file 5 — Additional file 5: Figure S1. Scatterplot of statistical additive effects in different generations for the genetic model with additive and dominance effects (Model AD) under RANDOM selection. Figure S2. Scatterplot of statistical additive effects in different generations for the genetic model with additive and dominance effects (Model AD) under MASS selection. Figure S3. Scatterplot of statistical additive effects in different generations for the genetic model with additive and dominance effects (Model AD) under PBLUP selection with own performance (PBLUP_OP). Figure S4. Scatterplot of statistical additive effects in different generations for the genetic model with additive and dominance effects (Model AD) under GBLUP selection without own performance (GBLUP_NoOP). Figure S5. Scatterplot of statistical additive effects in different generations for the genetic model with additive and dominance effects (Model AD) under GBLUP selection with own performance (GBLUP_OP). Figure S6. Scatterplot of statistical additive effects in different generations for the genetic model with additive, dominance and epistatic effects (Model ADE) under RANDOM selection. Figure S7. Scatterplot of statistical additive effects in different generations for the genetic model with additive, dominance and epistatic effects (Model ADE) under MASS selection. Figure S8. Scatterplot of statistical additive effects in different generations for the genetic model with additive, dominance and epistatic effects (Model ADE) under PBLUP selection with own performance (PBLUP_OP). Figure S9. Scatterplot of statistical additive effects in different generations for the genetic model with additive, dominance and epistatic effects (Model ADE) under GBLUP selection without own performance (GBLUP_NoOP). Figure S10. Scatterplot of statistical additive effects in different generations for the genetic model with additive, dominance and epistatic effects (Model ADE) under GBLUP selection with own performance (GBLUP_OP) [file 12711_2022_709_MOESM5_ESM.docx]

**Additional file 5: Scatterplot of statistical additive effects in different generations**


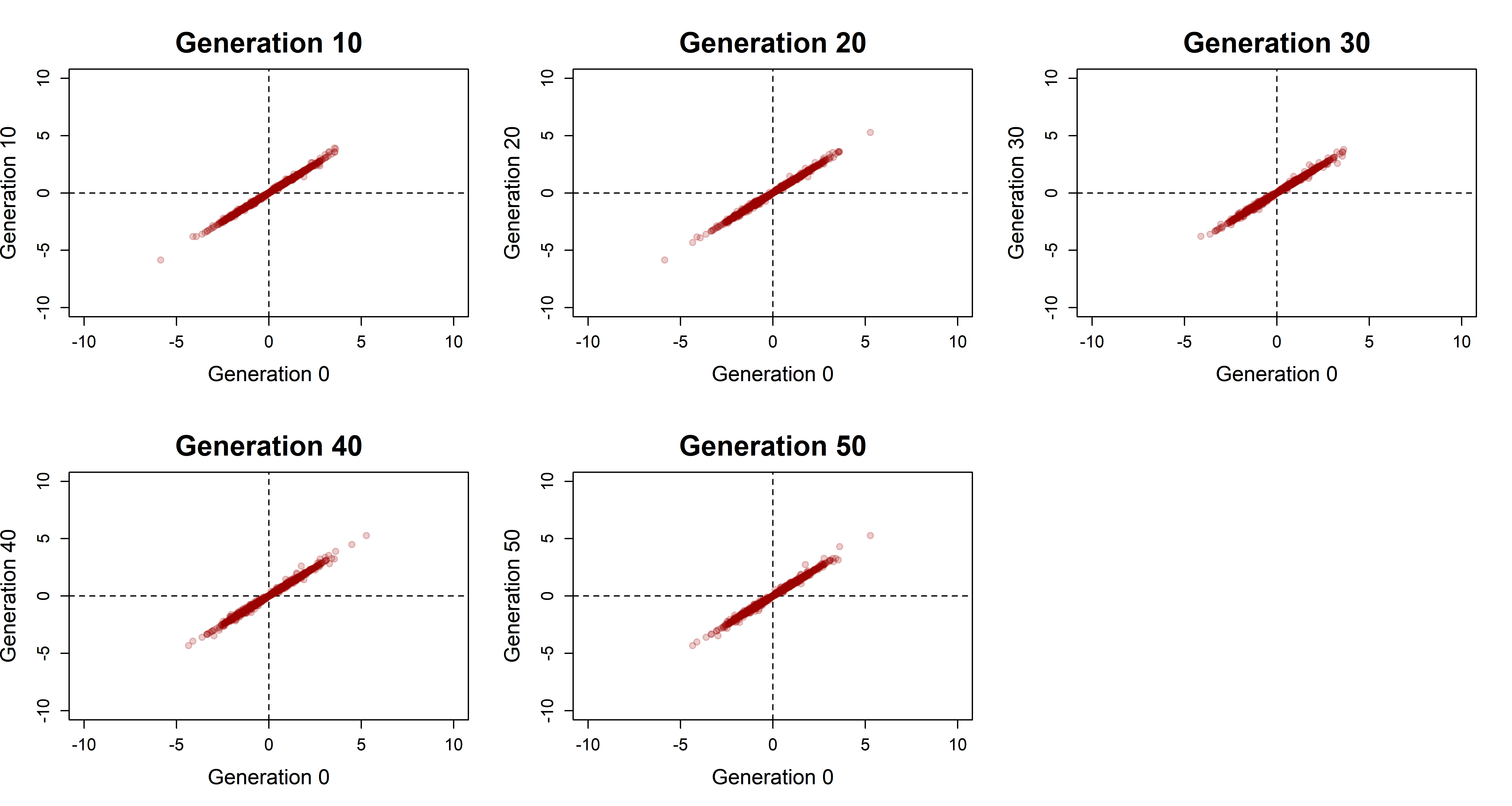


**FIGURE S1**

Scatterplot of statistical additive effects in different generations for the genetic model with additive and dominance effects (Model AD) under RANDOM selection.


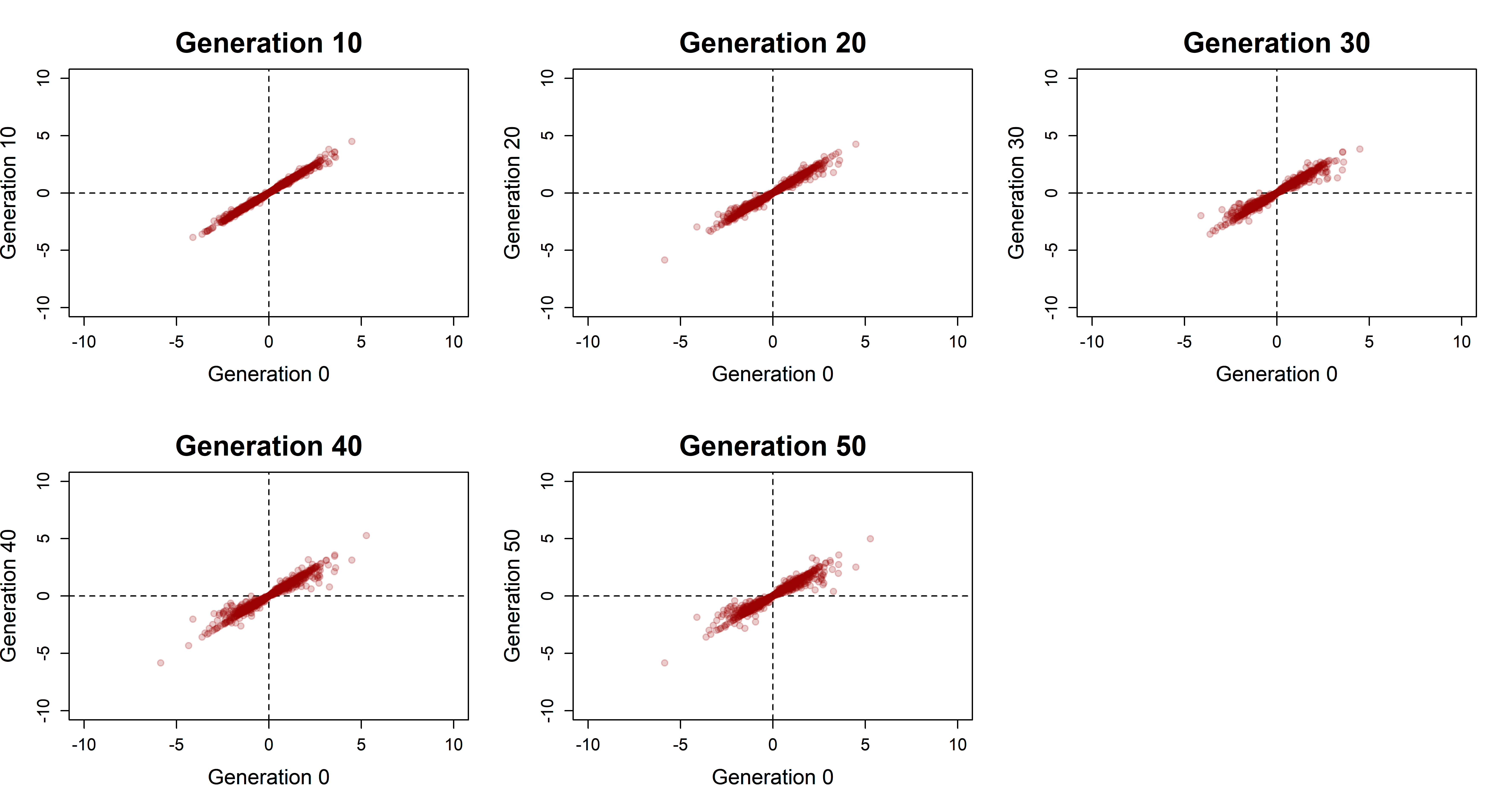


**FIGURE S2**

Scatterplot of statistical additive effects in different generations for the genetic model with additive and dominance effects (Model AD) under MASS selection.


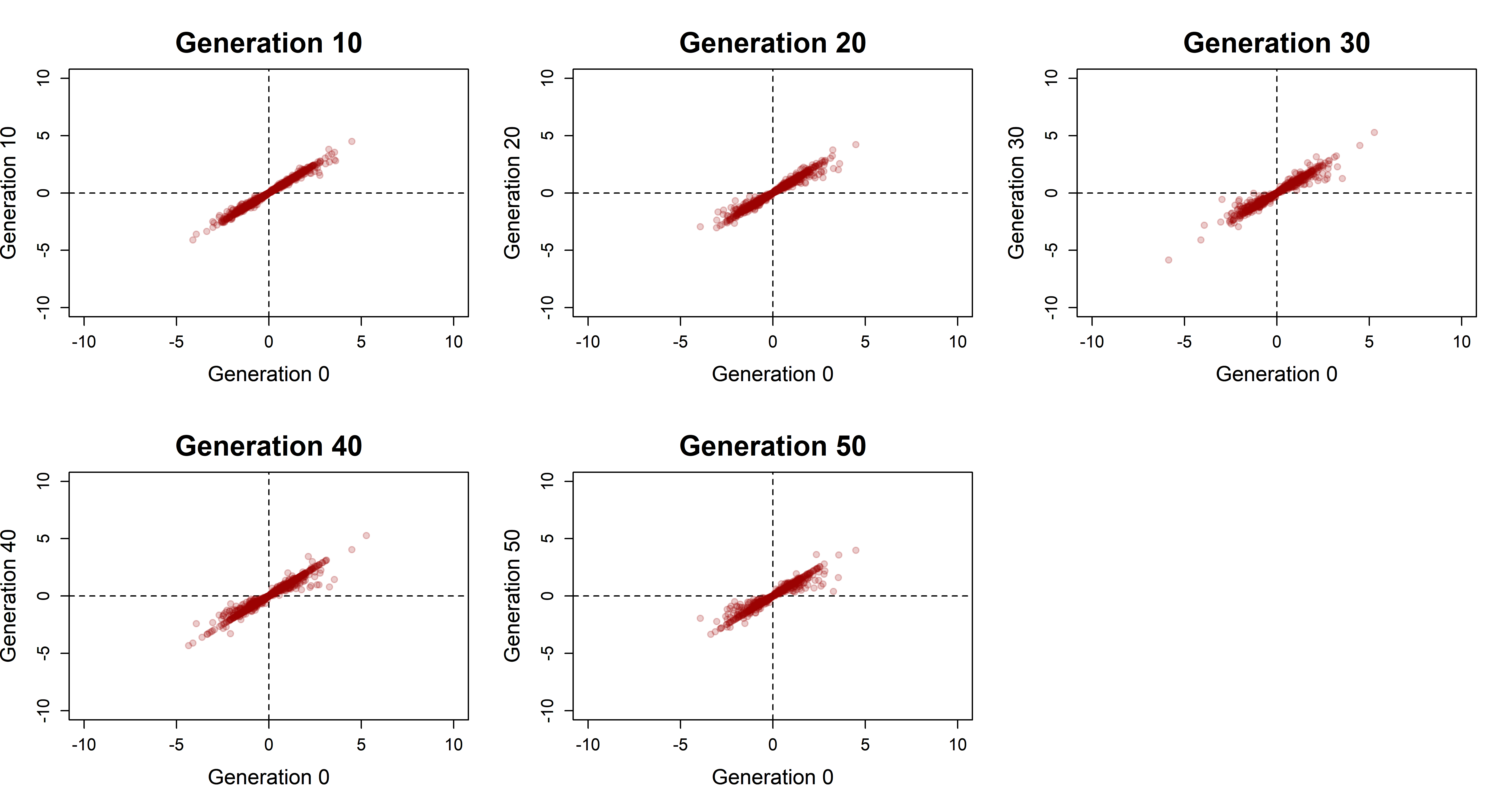


**FIGURE S3**

Scatterplot of statistical additive effects in different generations for the genetic model with additive and dominance effects (Model AD) under PBLUP selection with own performance (PBLUP_OP).


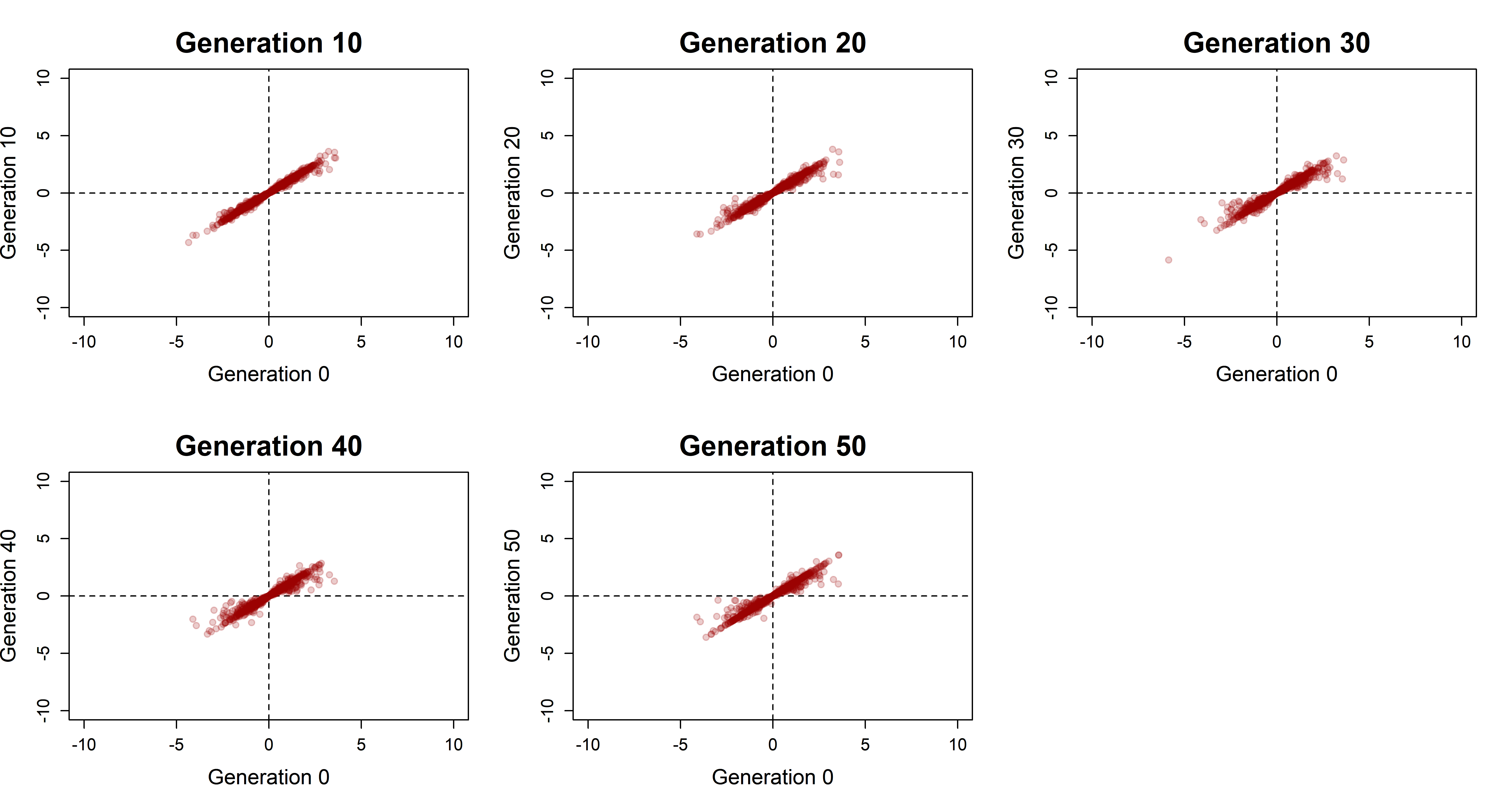


**FIGURE S4**

Scatterplot of statistical additive effects in different generations for the genetic model with additive and dominance effects (Model AD) under GBLUP selection without own performance (GBLUP_NoOP).


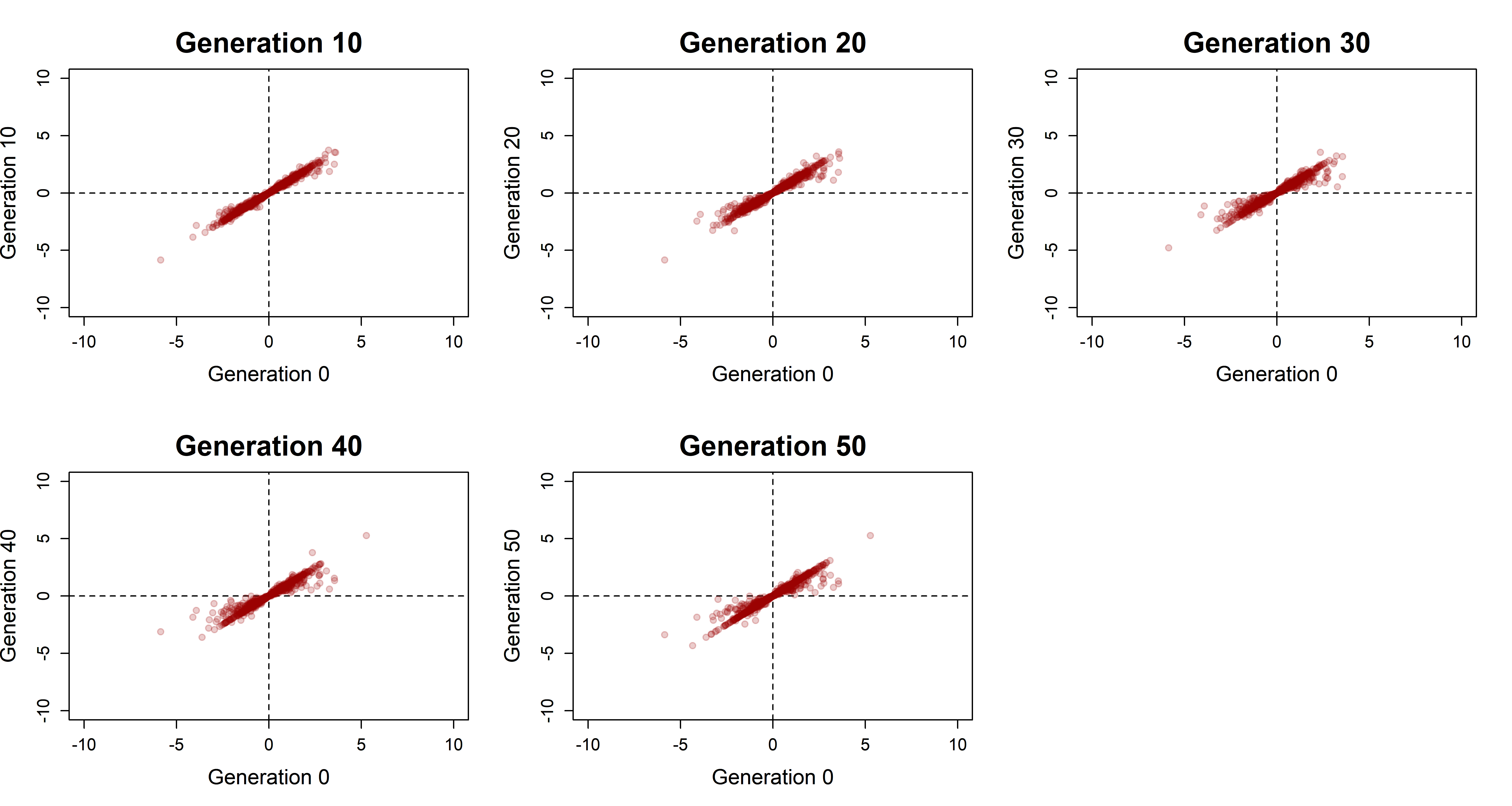


**FIGURE S5**

Scatterplot of statistical additive effects in different generations for the genetic model with additive and dominance effects (Model AD) under GBLUP selection with own performance (GBLUP_OP).


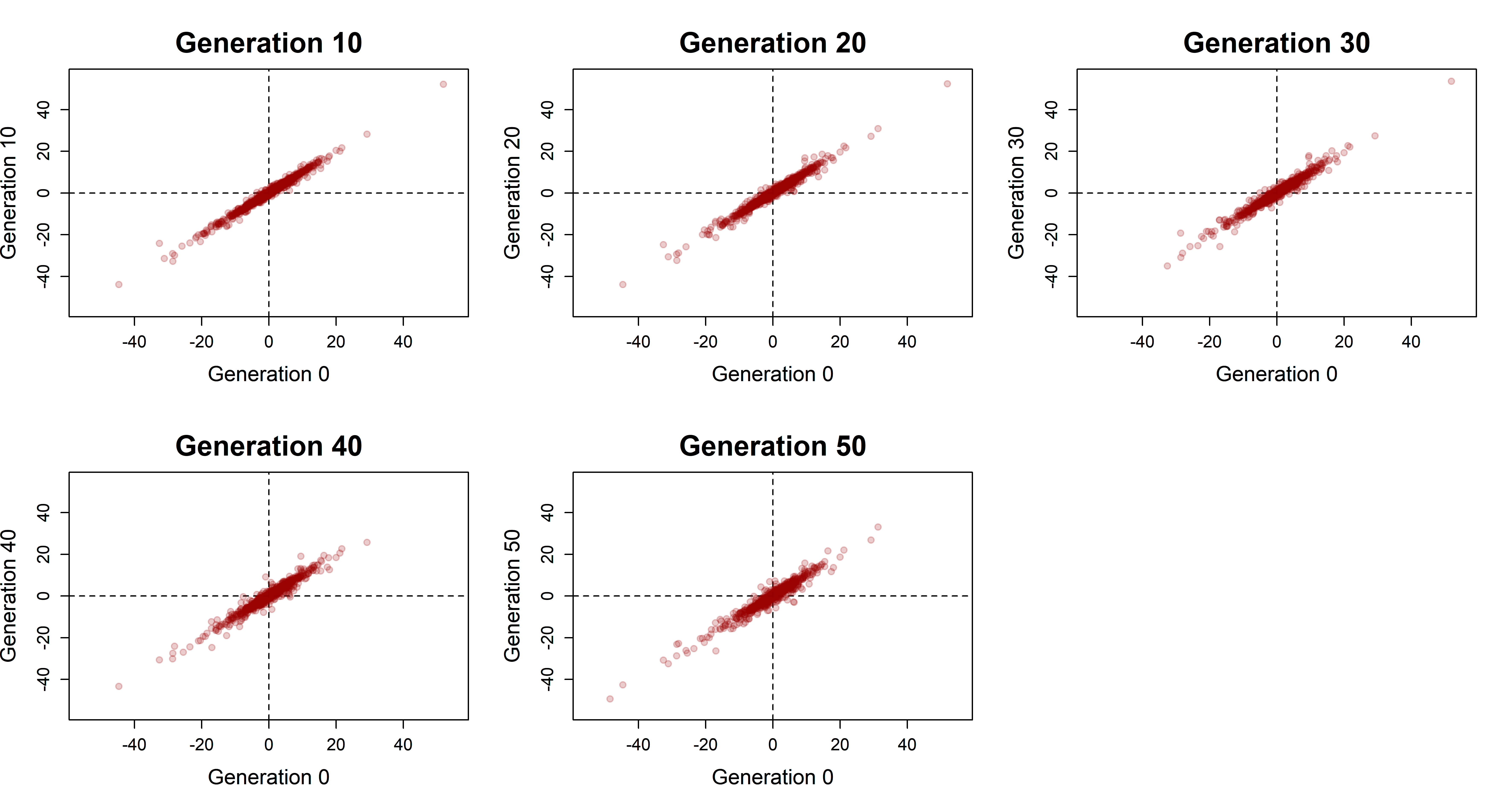


**FIGURE S6**

Scatterplot of statistical additive effects in different generations for the genetic model with additive, dominance and epistatic effects (Model ADE) under RANDOM selection.


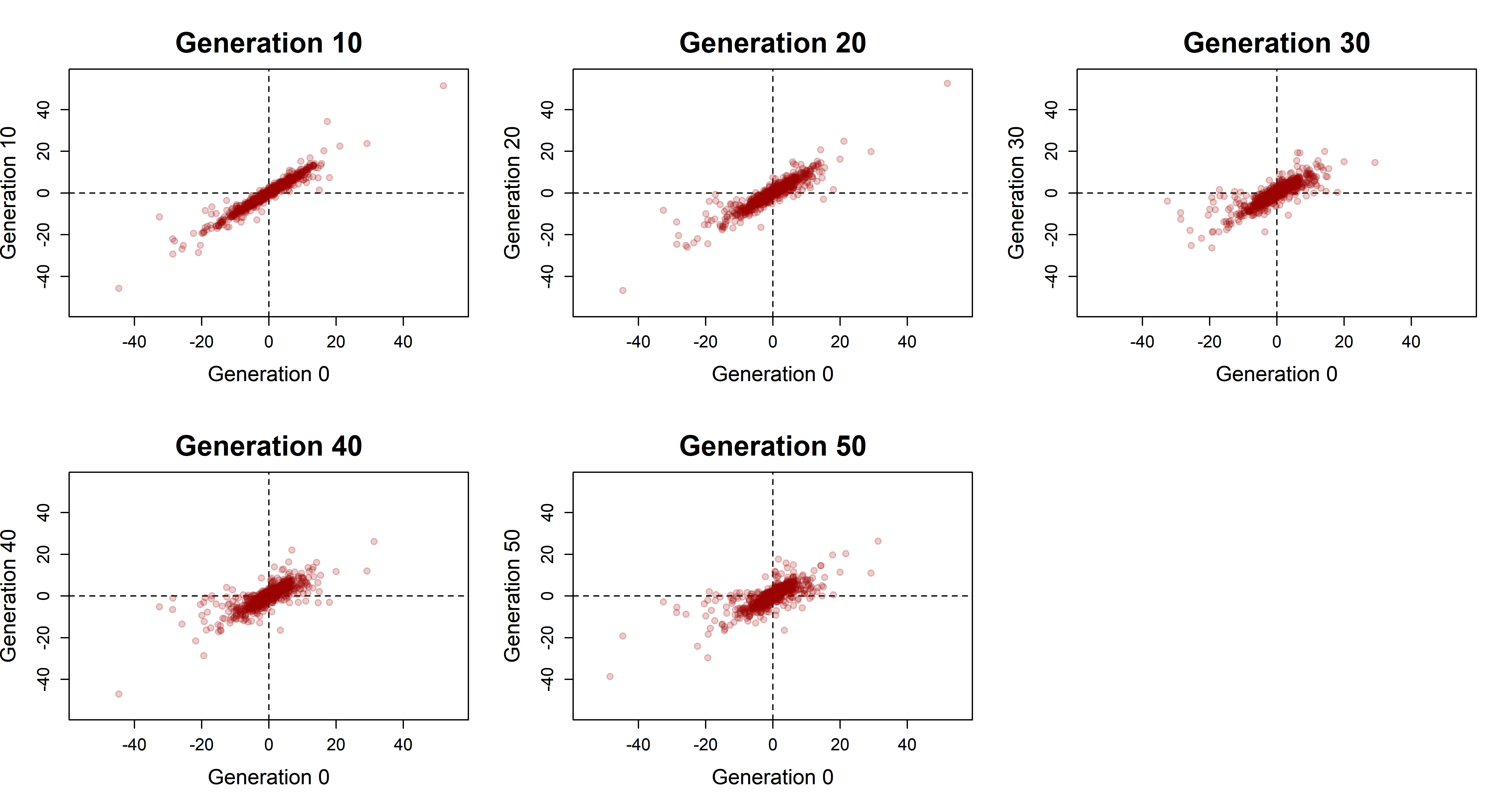


**FIGURE S7**

Scatterplot of statistical additive effects in different generations for the genetic model with additive, dominance and epistatic effects (Model ADE) under MASS selection.


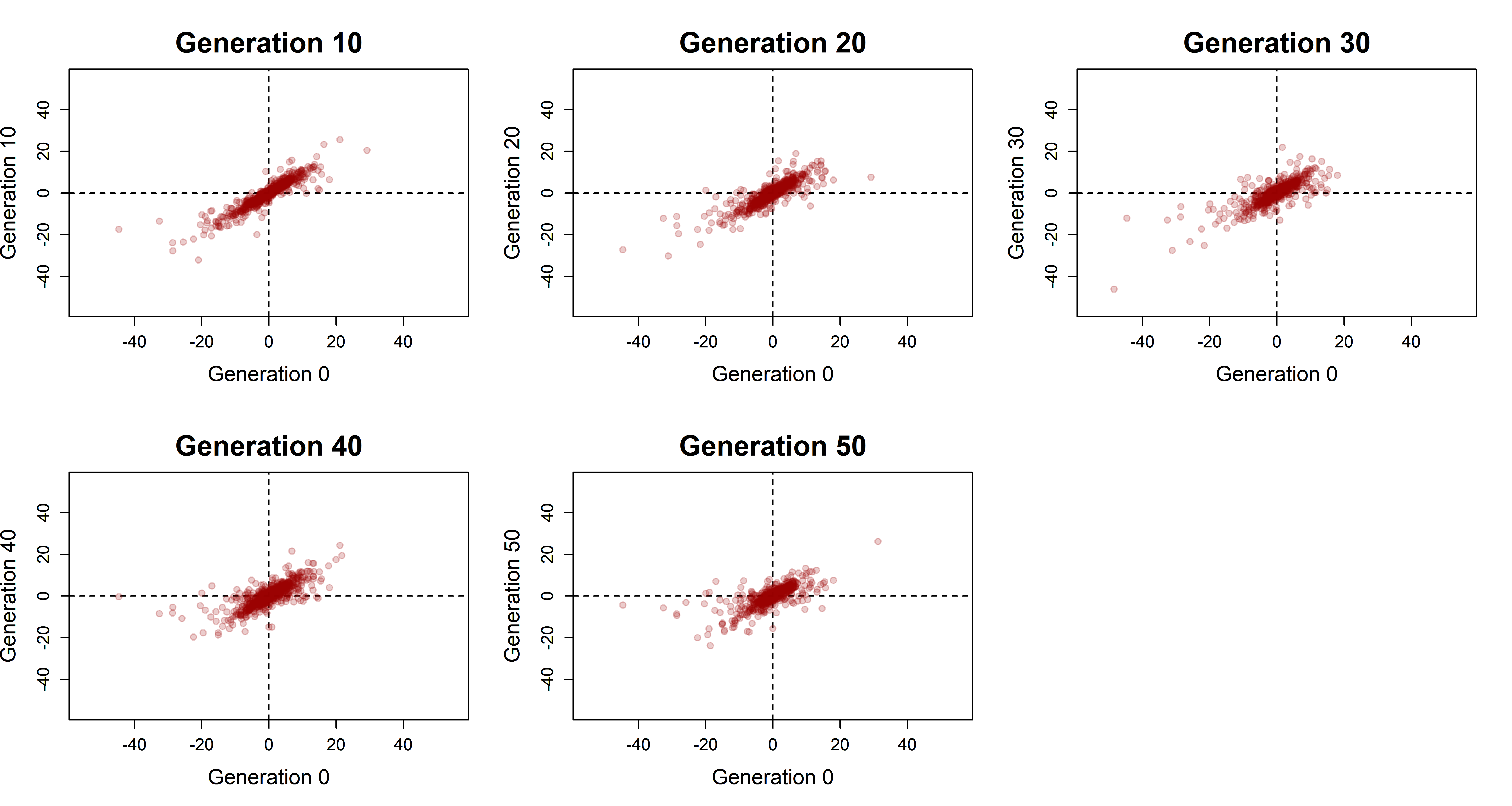
**FIGURE S8**

Scatterplot of statistical additive effects in different generations for the genetic model with additive, dominance and epistatic effects (Model ADE) under PBLUP selection with own performance (PBLUP_OP).


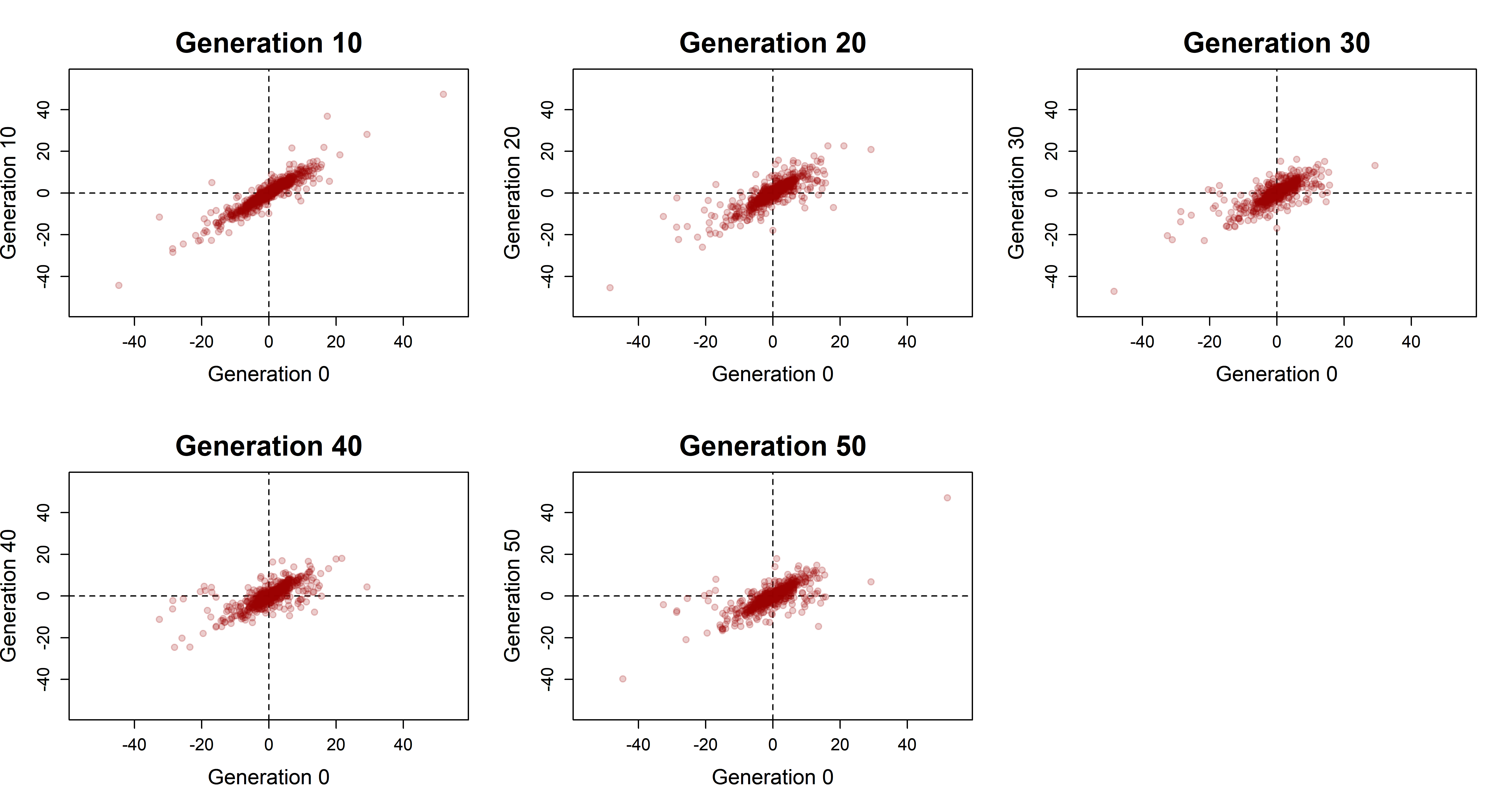


**FIGURE S9**

Scatterplot of statistical additive effects in different generations for the genetic model with additive, dominance and epistatic effects (Model ADE) under GBLUP selection without own performance (GBLUP_NoOP).


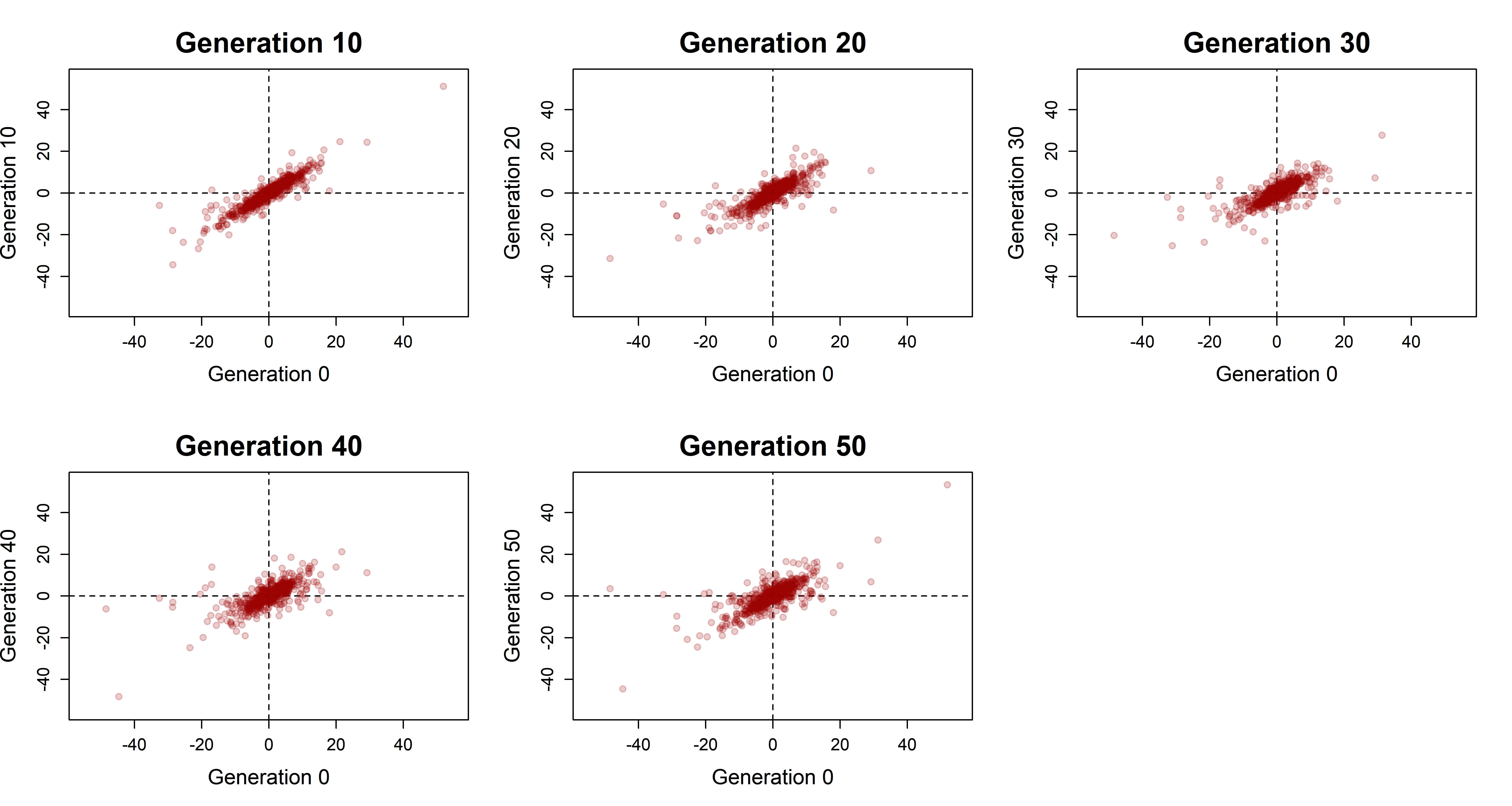


**FIGURE S10**

Scatterplot of statistical additive effects in different generations for the genetic model with additive, dominance and epistatic effects (Model ADE) under GBLUP selection with own performance (GBLUP_OP).
