# Supplementary material for: The long-term effects of genomic selection: 1. Response to selection, additive genetic variance, and genetic architecture
Source: Genet Sel Evol. 2022 Mar 7;54:19. doi: 10.1186/s12711-022-00709-7 (PMC8900405; doi:10.1186/s12711-022-00709-7)
Supplement: Supplementary file 6 — Additional file 6: Table S1. Maximum genetic gain1 that is still possible after 50 generations of selection for the five selection methods and three genetic models2. The five selection methods were: RANDOM selection, MASS selection, PBLUP selection with own performance (PBLUP_OP), GBLUP selection without own performance (GBLUP_NoOP) or with own performance (GBLUP_OP). The three genetic models were a model with only additive effects (A), with additive and dominance effects (AD), or with additive, dominance and epistatic effects (ADE). 1The maximum genetic gain in generation 50 is estimated as the genetic gain when all loci would be fixed for the favourable allele, using the statistical additive effects of generation 50 and neglecting mutations. 2Results are shown as averages across the 20 replicates with their corresponding standard errors of the mean between brackets. Table S2. Percentual change in the components of the genetic variance after 10 and 50 generations of selection for the five selection methods and three genetic models1. The five selection methods were: RANDOM selection, MASS selection, PBLUP selection with own performance (PBLUP_OP), GBLUP selection without own performance (GBLUP_NoOP) or with own performance (GBLUP_OP). The three genetic models were a model with only additive effects (A), with additive and dominance effects (AD), or with additive, dominance and epistatic effects (ADE). 1Results are shown as averages of 20 replicates with their corresponding standard errors of the mean between brackets. Increases in the value of a component are represented in bold. Table S3. Average pedigree inbreeding coefficient after 50 generations of selection for the five selection methods and three genetic models1. The five selection methods were: RANDOM selection, MASS selection, PBLUP selection with own performance (PBLUP_OP), GBLUP selection without own performance (GBLUP_NoOP) or with own performance (GBLUP_OP). The three genetic models were a model with only [file 12711_2022_709_MOESM6_ESM.docx]

**Additional file 6: Additional tables**

**TABLE S1** – Maximum genetic gain^1^ that is still possible after 50 generations of selection for the five selection methods and three genetic models^2^. The five selection methods were: RANDOM selection, MASS selection, PBLUP selection with own performance (PBLUP_OP), GBLUP selection without own performance (GBLUP_NoOP) or with own performance (GBLUP_OP). The three genetic models were a model with only additive effects (A), with additive and dominance effects (AD), or with additive, dominance and epistatic effects (ADE).

|  |  |  | **Model** |  |  |  |
| --- | --- | --- | --- | --- | --- | --- |
|  | **A** | | **AD** | | **ADE** | |
| **RANDOM** | 2243 | (13.8) | 2582 | (18.5) | 8851 | (100.0) |
| **MASS** | 1651 | (17.0) | 1992 | (16.7) | 6643 | (76.0) |
| **PBLUP_OP** | 1060 | (11.9) | 1336 | (15.4) | 4446 | (65.5) |
| **GBLUP_NoOP** | 1134 | (9.9) | 1346 | (15.5) | 4910 | (60.0) |
| **GBLUP_OP** | 1151 | (13.3) | 1399 | (12.1) | 4874 | (65.5) |

^1^ The maximum genetic gain in generation 50 is estimated as the genetic gain when all loci would be fixed for the favourable allele, using the statistical additive effects of generation 50 and neglecting mutations.

^2^ Results are shown as averages across the 20 replicates with their corresponding standard errors of the mean between brackets.

**TABLE S2** – Percentual change in the components of the genetic variance after 10 and 50 generations of selection for the five selection methods and three genetic models^1^. The five selection methods were: RANDOM selection, MASS selection, PBLUP selection with own performance (PBLUP_OP), GBLUP selection without own performance (GBLUP_NoOP) or with own performance (GBLUP_OP). The three genetic models were a model with only additive effects (A), with additive and dominance effects (AD), or with additive, dominance and epistatic effects (ADE).

|  | **Generation 10** | | | | | | | | | | | |  | **Generation 50** | | | | | | | | | | | |
| --- | --- | --- | --- | --- | --- | --- | --- | --- | --- | --- | --- | --- | --- | --- | --- | --- | --- | --- | --- | --- | --- | --- | --- | --- | --- |
|  | $\sigma_{A,genetic}^{2}$ | | $\sigma_{A,genic}^{2}$ | | $n$ | | $\bar{H_{E}}$ | | $\bar{\alpha^{2}}$ | | $Cov(H_{E},\alpha^{2})$ | |  | $\sigma_{A,genetic}^{2}$ | | $\sigma_{A,genic}^{2}$ | | $n$ | | $\bar{H_{E}}$ | | $\bar{\alpha^{2}}$ | | $Cov(H_{E},\alpha^{2})$ | |
| ***Model A*** |  |  |  |  |  |  |  |  |  |  |  |  |  |  |  |  |  |  |  |  |  |  |  |  |  |
| **RANDOM** | -3 | (3.4) | -1 | (0.3) | 0 | (0.3) | -1 | (0.2) | 0 | (0.4) | -67 | (50.7) |  | -3 | (3.5) | -3 | (0.5) | -1 | (0.3) | -2 | (0.4) | 0 | (0.6) | -91 | (45.1) |
| **MASS** | -31 | (2.2) | -5 | (0.5) | -8 | (0.3) | **3** | (0.4) | 0 | (0.5) | **26** | (58.1) |  | -60 | (1.4) | -37 | (1.3) | -21 | (0.6) | -16 | (0.8) | -4 | (0.6) | -81 | (85.3) |
| **PBLUP_OP** | -37 | (2.4) | -11 | (0.7) | -21 | (0.5) | **13** | (0.5) | 0 | (0.5) | **66** | (71.2) |  | -79 | (0.9) | -65 | (1.0) | -49 | (0.4) | -24 | (1.1) | -3 | (0.7) | **3** | (187.9) |
| **GBLUP_NoOP** | -39 | (2.0) | -14 | (0.5) | -17 | (0.3) | **6** | (0.4) | 0 | (0.5) | **60** | (89.9) |  | -82 | (0.7) | -73 | (0.6) | -44 | (0.4) | -41 | (0.8) | -5 | (0.8) | -75 | (171.9) |
| **GBLUP_OP** | -46 | (1.5) | -12 | (0.5) | -15 | (0.4) | **5** | (0.4) | 0 | (0.5) | **43** | (63.6) |  | -83 | (0.4) | -70 | (0.7) | -43 | (0.4) | -38 | (0.8) | -5 | (0.5) | -15 | (105.1) |
|  |  |  |  |  |  |  |  |  |  |  |  |  |  |  |  |  |  |  |  |  |  |  |  |  |  |
| ***Model AD*** |  |  |  |  |  |  |  |  |  |  |  |  |  |  |  |  |  |  |  |  |  |  |  |  |  |
| **RANDOM** | **4** | (3.7) | 0 | (0.3) | 0 | (0.3) | 0 | (0.3) | **1** | (0.5) | **38** | (29.0) |  | -2 | (3.5) | -2 | (0.4) | 0 | (0.3) | -2 | (0.4) | **1** | (0.7) | **3** | (26.2) |
| **MASS** | -30 | (2.1) | -4 | (0.7) | -7 | (0.4) | **5** | (0.5) | 0 | (0.5) | **63** | (83.5) |  | -56 | (1.7) | -28 | (1.5) | -16 | (0.4) | -9 | (0.9) | -5 | (0.9) | -140 | (96.4) |
| **PBLUP_OP** | -36 | (1.7) | -10 | (0.7) | -20 | (0.3) | **15** | (0.5) | -1 | (0.8) | -13 | (90.1) |  | -74 | (0.8) | -54 | (1.2) | -43 | (0.5) | -9 | (1.1) | -5 | (1.1) | -110 | (162.8) |
| **GBLUP_NoOP** | -37 | (2.2) | -14 | (0.8) | -17 | (0.4) | **8** | (0.5) | -2 | (0.8) | -26 | (78.0) |  | -81 | (0.9) | -68 | (1.0) | -42 | (0.3) | -32 | (0.9) | -7 | (0.8) | -125 | (202.8) |
| **GBLUP_OP** | -44 | (1.5) | -11 | (0.7) | -14 | (0.3) | **7** | (0.5) | -2 | (0.8) | -13 | (93.6) |  | -81 | (0.7) | -64 | (0.9) | -40 | (0.4) | -29 | (0.8) | -8 | (0.6) | -36 | (138.6) |
|  |  |  |  |  |  |  |  |  |  |  |  |  |  |  |  |  |  |  |  |  |  |  |  |  |  |
| ***Model ADE*** |  |  |  |  |  |  |  |  |  |  |  |  |  |  |  |  |  |  |  |  |  |  |  |  |  |
| **RANDOM** | **2** | (2.5) | **1** | (0.6) | 0 | (0.3) | 0 | (0.3) | 0 | (1.0) | -2 | (2.4) |  | 0 | (2.8) | -1 | (1.0) | -1 | (0.4) | -2 | (0.3) | 0 | (1.6) | -1 | (4.1) |
| **MASS** | -21 | (2.9) | -6 | (1.9) | -7 | (0.3) | **5** | (0.4) | -3 | (1.3) | **5** | (4.6) |  | -56 | (1.8) | -37 | (1.5) | -15 | (0.4) | **1** | (0.7) | -15 | (1.7) | **8** | (5.9) |
| **PBLUP_OP** | -38 | (2.6) | -19 | (1.7) | -23 | (0.4) | **17** | (0.5) | -8 | (2.0) | **15** | (6.9) |  | -74 | (1.4) | -64 | (1.0) | -44 | (0.4) | **1** | (1.2) | -14 | (1.8) | **23** | (6.4) |
| **GBLUP_NoOP** | -40 | (1.6) | -21 | (1.1) | -19 | (0.5) | **8** | (0.5) | -3 | (1.4) | **17** | (4.4) |  | -75 | (0.9) | -66 | (0.9) | -40 | (0.4) | -15 | (0.9) | -5 | (1.8) | **16** | (4.0) |
| **GBLUP_OP** | -45 | (1.8) | -21 | (1.8) | -17 | (0.4) | **8** | (0.5) | -5 | (1.7) | **17** | (4.8) |  | -76 | (1.1) | -66 | (0.9) | -39 | (0.5) | -12 | (1.1) | -11 | (1.7) | **12** | (3.1) |

^1^ Results are shown as averages of 20 replicates with their corresponding standard errors of the mean between brackets. Increases in the value of a component are represented in bold.

**TABLE S3** – Average pedigree inbreeding coefficient after 50 generations of selection for the five selection methods and three genetic models^1^. The five selection methods were: RANDOM selection, MASS selection, PBLUP selection with own performance (PBLUP_OP), GBLUP selection without own performance (GBLUP_NoOP) or with own performance (GBLUP_OP). The three genetic models were a model with only additive effects (A), with additive and dominance effects (AD), or with additive, dominance and epistatic effects (ADE).

|  | **Model** | | | | |  |  |
| --- | --- | --- | --- | --- | --- | --- | --- |
|  | **A** | | **AD** | | **ADE** | | **Average** |
| **RANDOM** | 0.10 | (0.001) | 0.10 | (0.001) | 0.10 | (0.001) | 0.06 |
| **MASS** | 0.16 | (0.002) | 0.16 | (0.002) | 0.15 | (0.002) | 0.10 |
| **PBLUP_OP** | 0.41 | (0.006) | 0.38 | (0.006) | 0.43 | (0.007) | 0.25 |
| **GBLUP_NoOP** | 0.28 | (0.005) | 0.28 | (0.004) | 0.30 | (0.004) | 0.18 |
| **GBLUP_OP** | 0.26 | (0.004) | 0.25 | (0.003) | 0.28 | (0.003) | 0.16 |

^1^ Results are shown as averages of 20 replicates with their corresponding standard errors of the mean between brackets.

**TABLE S4** – Average and variance of change in allele frequency of causal loci^1^ across 50 generations of selection for the five selection methods and three genetic models^2^. The five selection methods were: RANDOM selection, MASS selection, PBLUP selection with own performance (PBLUP_OP), GBLUP selection without own performance (GBLUP_NoOP) or with own performance (GBLUP_OP). The three genetic models were a model with only additive effects (A), with additive and dominance effects (AD), or with additive, dominance and epistatic effects (ADE).

|  | **Average change in allele frequency** | | **Variance of change in allele frequency** | |
| --- | --- | --- | --- | --- |
| ***Model A*** |  |  |  |  |
| **Random** | 0.023 | (0.000) | 0.005 | (0.000) |
| **MASS** | 0.054 | (0.001) | 0.034 | (0.001) |
| **PBLUP_OP** | 0.062 | (0.000) | 0.045 | (0.001) |
| **GBLUP_NoOP** | 0.063 | (0.001) | 0.048 | (0.001) |
| **GBLUP_OP** | 0.065 | (0.001) | 0.050 | (0.001) |
|  |  |  |  |  |
| ***Model AD*** |  |  |  |  |
| **Random** | 0.023 | (0.000) | 0.005 | (0.000) |
| **MASS** | 0.053 | (0.000) | 0.032 | (0.001) |
| **PBLUP_OP** | 0.061 | (0.000) | 0.042 | (0.001) |
| **GBLUP_NoOP** | 0.064 | (0.000) | 0.047 | (0.000) |
| **GBLUP_OP** | 0.065 | (0.000) | 0.048 | (0.000) |
|  |  |  |  |  |
| ***Model ADE*** |  |  |  |  |
| **Random** | 0.023 | (0.000) | 0.005 | (0.000) |
| **MASS** | 0.042 | (0.000) | 0.020 | (0.000) |
| **PBLUP_OP** | 0.053 | (0.000) | 0.032 | (0.000) |
| **GBLUP_NoOP** | 0.054 | (0.000) | 0.034 | (0.001) |
| **GBLUP_OP** | 0.055 | (0.000) | 0.035 | (0.000) |

^1^ Causal loci included only the causal loci segregating in generation 0.

^2^ Results are shown as averages of 20 replicates with their corresponding standard errors of the mean between brackets.
